# Challenges of Robust RNAi-Mediated Gene Silencing in *Aedes* Mosquitoes

**DOI:** 10.3390/ijms25105218

**Published:** 2024-05-10

**Authors:** Lucas Henrique Figueiredo Prates, Jakob Fiebig, Henrik Schlosser, Eleni Liapi, Tanja Rehling, Célia Lutrat, Jeremy Bouyer, Qiang Sun, Han Wen, Zhiyong Xi, Marc F. Schetelig, Irina Häcker

**Affiliations:** 1 Department of Insect Biotechnology in Plant Protection, Justus Liebig University Giessen, 35394 Giessen, Germany; lucas.prates@agrar.uni-giessen.de (L.H.F.P.); jakob.fiebig@umwelt.uni-giessen.de (J.F.); h.schlosser@uq.edu.au (H.S.); tanja.rehling@agrar.uni-giessen.de (T.R.); irina.haecker@agrar.uni-giessen.de (I.H.); 2 Department of Biochemistry and Biotechnology, University of Thessaly, 41500 Larissa, Greece; eliapi@outlook.com; 3 ASTRE, CIRAD, 34398 Montpellier, Francejeremy.bouyer@cirad.fr (J.B.); 4 ASTRE, CIRAD, INRAE, Univ. Montpellier, Plateforme Technologique CYROI, 97491 Sainte-Clotilde, La Réunion, France; 5 Department of Microbiology and Molecular Genetics, Michigan State University, East Lansing, MI 48824, USA; sqiang617@gmail.com (Q.S.); wenhan@msu.edu (H.W.); xizy@msu.edu (Z.X.)

**Keywords:** RNA interference, *Aedes aegypti*, *Aedes albopictus*, RNAi-based pest control, RNAi delivery methods, reproducibility of RNAi protocols

## Abstract

In this study, we report the complexities and challenges associated with achieving robust RNA interference (RNAi)-mediated gene knockdown in the mosquitoes *Aedes aegypti* and *Aedes albopictus*, a pivotal approach for genetic analysis and vector control. Despite RNAi’s potential for species-specific gene targeting, our independent efforts to establish oral delivery of RNAi for identifying genes critical for mosquito development and fitness encountered significant challenges, failing to reproduce previously reported potent RNAi effects. We independently evaluated a range of RNAi-inducing molecules (siRNAs, shRNAs, and dsRNAs) and administration methods (oral delivery, immersion, and microinjection) in three different laboratories. We also tested various mosquito strains and utilized microorganisms for RNA delivery. Our results reveal a pronounced inconsistency in RNAi efficacy, characterized by minimal effects on larval survival and gene expression levels in most instances despite strong published effects for the tested targets. One or multiple factors, including RNase activity in the gut, the cellular internalization and processing of RNA molecules, and the systemic dissemination of the RNAi signal, could be involved in this variability, all of which are barely understood in mosquitoes. The challenges identified in this study highlight the necessity for additional research into the underlying mechanisms of mosquito RNAi to develop more robust RNAi-based methodologies. Our findings emphasize the intricacies of RNAi application in mosquitoes, which present a substantial barrier to its utilization in genetic control strategies.

## 1. Introduction

RNA interference (RNAi) is a naturally occurring defense mechanism against foreign genetic material that has been exploited as a molecular technique primarily for reverse genetics studies by post-transcriptional gene silencing (PTGS) [1,2,3,4,5]. RNAi-based gene silencing can be triggered by double-stranded RNA (dsRNA), which can occur naturally or be supplied by the user, and that causes the degradation of the complementary gene transcript. Once incorporated, the dsRNA is first processed into smaller sequences of approximately 19 to 25 nucleotides, called small interfering RNAs (siRNAs). Then, one strand of the siRNA is loaded into the RNA-induced silencing complex (RISC), which localizes the complementary messenger RNA (mRNA), leading to its degradation and gene silencing [3,5,6,7,8,9,10]. Instead of dsRNAs, which are usually hundreds of base pairs long, two complementary short RNA sequences of approximately 19 to 22 nucleotides linked by a short loop ranging from 4 to 11 nucleotides (short hairpin RNA, shRNA) could also be used to generate siRNA molecules to trigger RNAi [4,6,11]. Thus, different RNA architectures, such as dsRNAs, shRNAs, and siRNAs, can be used to induce RNAi. Herein, we refer to these molecules collectively as interfering RNAs (iRNAs).

RNAi has been successfully applied to reverse genetics studies on insects, including mosquitoes [12,13,14]. More recently, this technology has gained much attention as a promising new tool for insect pest control [4,15,16,17,18]. The potential of RNAi is based on the possibility of designing iRNA sequences to precisely target only the transcript of a particular species, avoiding off-targets in related species. Targeting genes essential for development or fundamental physiological or metabolic processes aims to kill pest species during developmental stages without affecting other species. Thus, iRNAs could act as a species-specific insecticides. A different approach targets genes involved in sex determination [19,20,21,22] or male or female fertility [23,24,25] to reduce the population size of the next generation by producing a sex bias, thereby reducing the availability of mating partners or the number of offspring per individual, respectively. An RNAi-based sexing approach could also be used for genetic control methods based on the release of males [26], as there is currently no perfect genetic sexing strain available [27], and most of the programs upscaling mosquito genetic control are based on automated sorters using the phenotypical differences between pupae [28,29] or adults [30].

For mosquitoes, several encouraging studies have been published over the past ten years presenting a strong sex bias or high sterility or larval mortality upon RNAi-mediated gene knockdown [19,31,32,33,34,35,36]. Generally, iRNA molecules that knocked down mosquito transcript levels were delivered as siRNAs [22,31,35,37], shRNAs [31,35,37,38,39], or >200 bp dsRNAs [19,31,36,40,41,42,43,44,45]. Application strategies for the iRNA molecules included soaking of larvae in iRNA solutions, feeding of adults with iRNA–sugar solutions, feeding of iRNA-expressing microorganisms (bacteria, yeast, and microalgae) to mosquito larvae, and injections of iRNA during different developmental stages [46]. Moreover, nanoparticles were used for protection and the better delivery of iRNA [32,45,47,48,49,50,51,52]. The oral delivery of iRNA molecules to larvae or adults would be the method of choice for mosquito control applications.

However, the level of iRNA-mediated gene silencing can vary strongly across insect orders, families, or species, and even within one species. Variance in RNAi effects has also been observed in *Aedes* mosquitoes [36,47,51,53,54,55]. While the reasons are not well understood in many cases, several factors have been associated with the success, failure, or variability of RNAi in insects and have been reviewed extensively [4,18,56]. Among these are the presence of gut RNases decreasing the amount of bioavailable iRNA molecules, the efficiency of iRNA uptake from the gut lumen during oral application, the accessibility of the iRNA to the intracellular RNAi machinery, and the amplification and spread of the RNAi signal from the cells that initially take up the molecules (systemic RNAi). Also, the conformation and length of the iRNA and the targeted region have been associated with RNAi efficiency [4,18,56]. Overall, the mechanisms involved in insect RNAi, especially mosquito RNAi, are poorly understood. The successful application of RNAi as a tool for mosquito control, however, will require a robust RNAi response, independent of external and internal variables like temperature or humidity, the availability of other food sources, the genetic background of the targeted mosquito populations, or factors like the nutritional condition, development stage, or overall fitness of the targeted individuals.

In three different laboratories, we initially and independently aimed to establish RNAi by oral delivery in the mosquitoes *Aedes aegypti* and *Ae. albopictus*. The goal was to identify genes that are essential for the development and fitness of the insects and that produce strong phenotypes upon knockdown. During this process, we collectively noticed that oral delivery of iRNAs failed to reproduce previously reported strong RNAi effects, causing us to troubleshoot the possible reasons extensively and systematically. This included different iRNA delivery methods besides oral application, in combination with various iRNA architectures, such as siRNAs, shRNAs, and dsRNAs. Surprisingly, also with other delivery strategies the published results for the tested positive target genes could not be replicated. This raises doubts about the robustness of RNAi as a methodology in *Aedes* and implies that the complex underlying mechanisms are not yet understood well enough to make it a reliable method, which would also have implications for the use of RNAi for mosquito control.

## 2. Results

### 2.1. Variable RNAi Effects in Ae. aegypti upon Oral Delivery of iRNA-Expressing Microorganisms

#### 2.1.1. Feeding Larvae with shRNA-Producing Yeast Strains

Based on several publications in recent years, the use of shRNA-expressing yeasts delivered orally to *Ae. aegypti* larvae seems to be one of the most reliable and effective ways to knock down gene expression via RNAi in this mosquito species [31,33,34,38]. To establish this oral delivery method in our lab, we set out to replicate the published results, following the detailed information provided in the literature for the design and cloning of shRNA sequences [35] and for the execution of yeast transformation, yeast culturing, and larval feeding assays [38].

**Figure 1:**
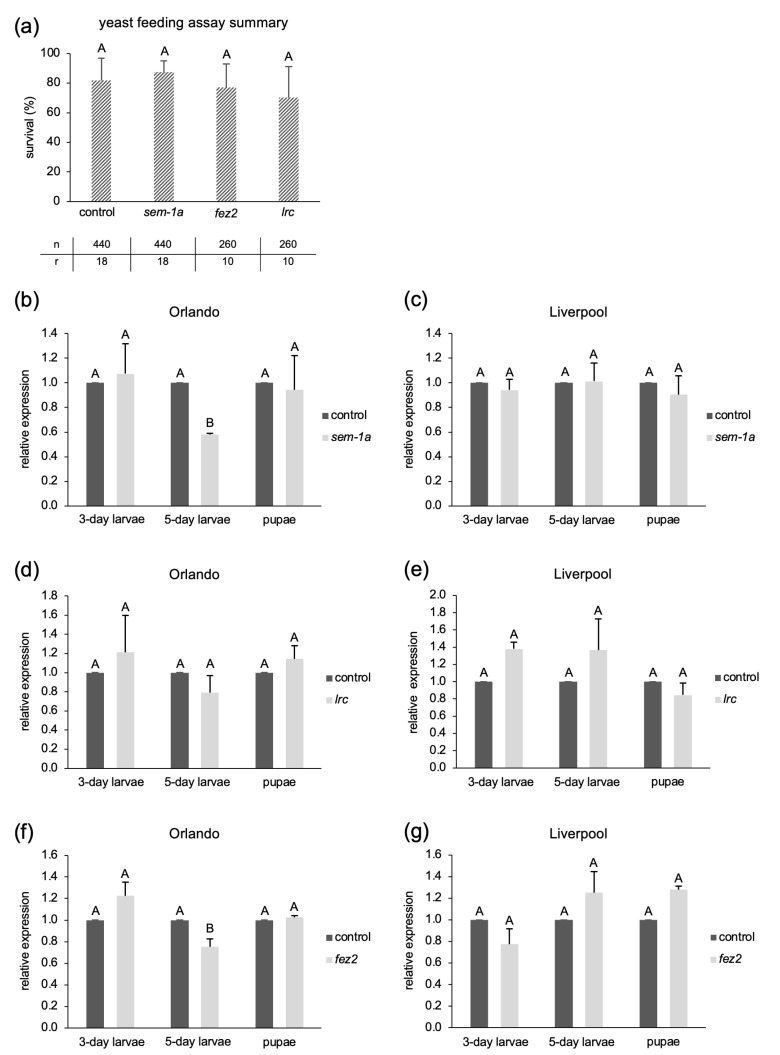
Results of oral delivery of shRNA-expressing yeast to *Ae. aegypti* larvae of the Orlando or Liverpool strains. Larvae were fed with the yeast 16–20 h after hatching until pupation. (**a**) Average survival rate to pupal stage (in percent) across all feeding assays performed per target gene, including eight biological replicates for *sem-1a* and the control shRNA, and four biological replicates for *lrc* and *fez2*. All biological replicates were performed with 2–3 technical replicates (20 to 30 individuals per replicate); n = total number of individuals used in all combined replicates, r = total number of replicates performed. Panels (**b**–**g**) show target gene mRNA levels in larvae sampled after 3 or 5 days of yeast feeding or after pupation, determined by RT-qPCR, calculated by the Pfaffl method [57], using *rps17* as reference gene. Data shown are based on three technical replicates. Five larvae were pooled from each replicate. In panels (**a**–**g**), “control” is the feeding with the unspecific shRNA, *sem-1a* = *semaphorin-1a*, *fez2* = *fasciculation and elongation protein zeta2*, *lrc* = *leukocyte receptor cluster*. Error bars indicate standard deviation, and different letters above the bars indicate statistically significant differences between gene-specific shRNA treatments and the unspecific control with *p*-value < 0.05 (one-way ANOVA, Bonferroni *t*-test in (**a**), and one-sample *t*-test in (**b**–**g**)).

Yeast shRNA feeding assays were performed with *semaphorin-1a* (*sem-1a*) [35], *fasciculation and elongation protein zeta2* (*fez2*), and *leukocyte receptor cluster member 8 homolog* (*lrc*) as target genes and with a scrambled shRNA as a negative control [31]. All three genes have been reported to cause up to 90% larval mortality until the L4 larval stage. The shRNA was provided to the larvae as dried yeast pellets, which were replaced with fresh ones as needed or, at the latest, every second day. *Sem-1a* and control shRNA assays were performed in eight biological replicates, and *lrc* and *fez2* assays were performed in four biological replicates, all distributed across two wild-type laboratory strains, namely, Orlando and Liverpool. The event of successful pupation was counted as survival. A significant reduction in larval survival or pupal transcript levels was observed in single experiments compared to the controls (Figure S1). Still, these reductions did not occur in the other replicates and were much lower than the published effects. Across all biological replicates, there was no significant difference in the survival rates between the control and the target genes for all three targets (*p*-values = 1.00 (*sem-1a*), 1.00 (*fez2*), 0.088 (*lrc*), one-way ANOVA, Bonferroni *t*-test) (Figure 1a).

In two large-scale feeding experiments with three technical replicates, performed once with Liverpool and once with Orlando larvae, we included sampling of batches of five larvae (on days 3 and 5 of the feeding) and five pupae for an RT-qPCR analysis. This analysis showed a moderate but significant reduction in *sem-1a* (*p*-value = 0.0141, one-sample *t*-test) and *fez2* (*p*-value = 0.0290, one-sample *t*-test) transcript levels in the 5-day larvae with the Orlando strain but not in the 3-day larvae or the pupae. The *lrc* transcript levels in Orlando did not differ from those in the controls. No effect was observed in the identical assay with the Liverpool lab strain (Figure 1b–g). The individual survival rates for these two experiments are shown in Figure S1, and the survival numbers and relative transcript ratios of all replicates performed are listed in Table S1.

#### 2.1.2. Feeding Larvae with Different Concentrations of dsRNA-Producing Bacteria

Other published oral delivery strategies that yielded sound RNAi effects are soaking early larvae in concentrated dsRNA or siRNA solutions [31,36] and feeding dsRNA-expressing bacterial strains to early larvae. In the latter assay, pelleted bacterial cells are mixed with LB agar and ground fish food to produce food pellets that are provided to the larvae daily until pupation [19]. The bacterial expression system is the widely used HT115 DE3 RNase III-deficient strain with the inducible L4440 expression vector [58,59,60].

One target gene, *beta-tubulin* (*βtub*, AAEL002851), was reported to yield high rates of *Ae. aegypti* late larval lethality by repeated soaking of L1 larvae in a 500 ng/µL solution of an 800 bp in vitro transcribed dsRNA [36]. We cloned the *βtub* dsRNA sequence from Singh et al. [36] into the L4440 expression vector and confirmed dsRNA expression by extraction from the bacterial cells [61].

**Figure 2:**
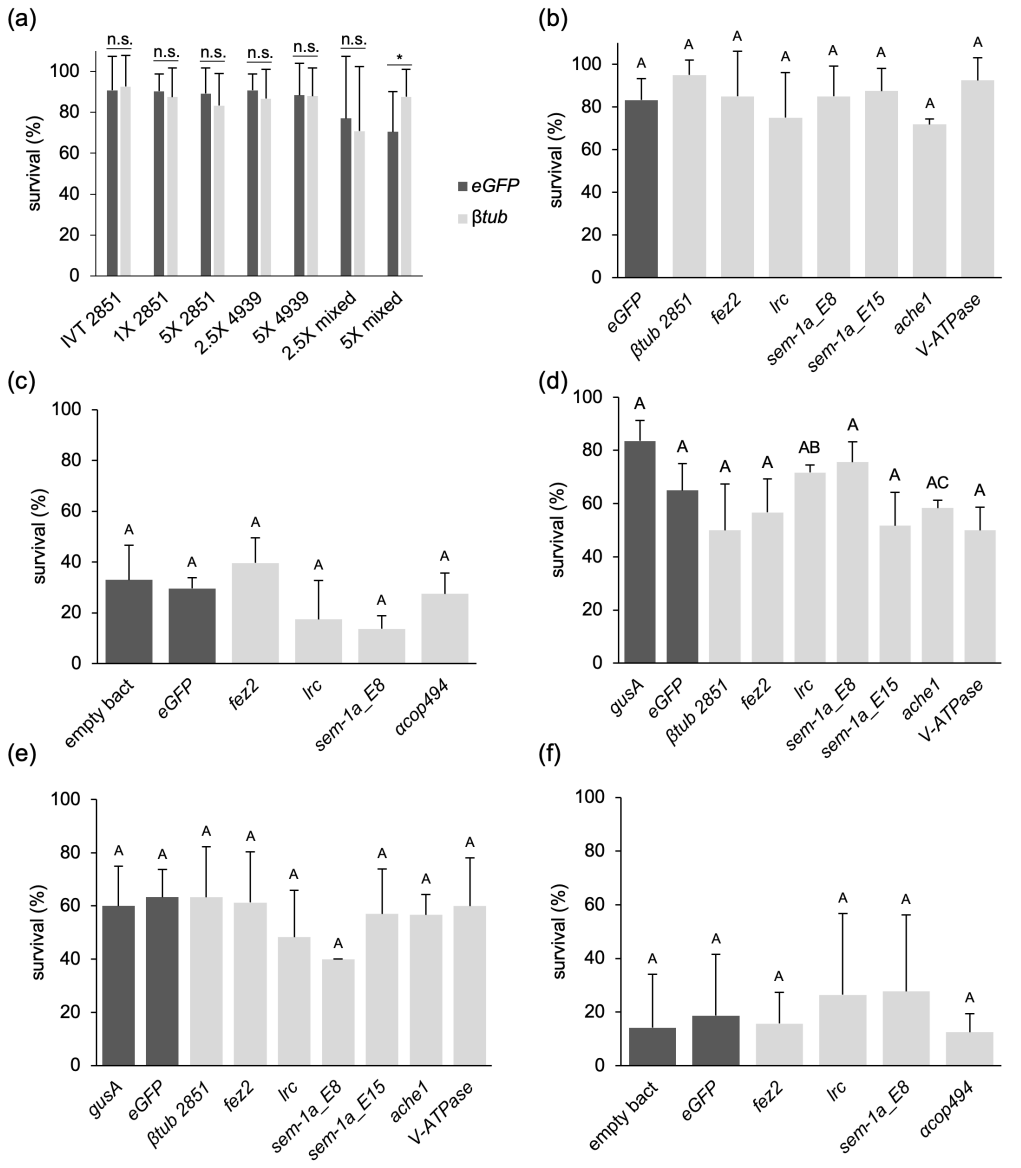
Survival rates of *Ae. aegypti* larvae fed with dsRNA-expressing bacteria. Panels (**a**,**b**): bacterial pellets with high nutrient content. (**a**) Food variant 1, containing different bacterial doses (1X, 2.5X, 5X). All results represent one biological replicate with 12 technical replicates each (except for *βtub 4939* 5X, which has two biological replicates). IVT represents the result of larval soaking in 500 ng/µL of in vitro transcribed AAEL002851 dsRNA (one biological replicate with 12 technical replicates) based on the protocol in [36]. “mixed” means that larvae were fed with a mixture of bacteria expressing dsRNAs against AAEL002851 and AAEL004939. (**b**) Food variant 2, data presented are based on two biological replicates with 20 larvae each. The bacteria used per food volume was 15X, i.e., 3-fold higher than the highest in (**a**). Panels (**c**–**f**) show feeding with reduced nutrient content. (**c**) Food variant 3, two biological replicates with 30 and 40 larvae each. (**d**) Food variant 4, addition of fish food on day 11; three biological replicates with 20 larvae each. (**e**) Food variant 4, addition of fish food on days 11, 13, 15; three biological replicates with 20 larvae each. (**f**) Food variant 5, two biological replicates with 40 larvae each. Average survival rates are shown (in percent), and error bars represent standard deviation. n.s. indicates no statistical significance, * indicates a significant difference with *p*-value < 0.05 (Welch’s *t*-test in (**a**)). Different letters above the bars indicate statistically significant differences with *p*-value < 0.05 (one-way ANOVA, Welch’s test in (**b**,**c**,**d**,**f**); one-way ANOVA in (**e**)). Additionally, 2851 = *βtub* gene AAEL002851, 4939 = *βtub* gene AAEL004939, *ache1* = *acetylcholine esterase 1*, *acop494 = coat protein alpha* (494 bp dsRNA), *eGFP* = *enhanced green fluorescent protein*, empty bact = bacteria not expressing any dsRNA, *fez2* = *fasciculation and elongation protein zeta2*, *gusA* = *E. coli beta-glucuronidase*, *lrc* = *leukocyte receptor cluster member 8 homolog, sem-1a* = *semaphorin-1a*; *sem-1a-E8* and *sem-1a-E15* are two different dsRNAs, targeting *sem-1a* exon 8 and exon 15, respectively. In exon 8, the siRNA target sequence is from [63]; in exon 15, the siRNA target sequence is from [35]. *V-ATPase* = *vacuolar-type ATPase*.

Following the outline given in the bacterial feeding protocol [19], we produced food pellets by mixing bacterial cells from a 100 mL culture with 5 mL of LB-agar and ground fish food (food variant 1, 1X bacterial concentration) and fed them to *Ae. aegypti* larvae until pupation, starting with L1 larvae hatched overnight, and replacing food pellets as needed or, at the latest, after 48 h. This procedure did not yield significantly higher mortality than the bacteria expressing a 400 bp *eGFP* control dsRNA (Figure 2a). Therefore, we increased the amount of bacterial culture per food volume by 2.5- and 5-fold and used another version of the *βtub* gene (AAEL004939) [19], as well as the combination of both *βtub* versions at different bacterial concentrations. However, none of the experiments yielded a significantly higher larval death compared to the *eGFP* dsRNA control treatments (Figure 2a, *p*-values = 0.630, 0.882, 0.303, 0.568, 0.738, 0.597, 0.0107, Welch’s *t*-test).

We finally increased the amount of bacterial cells by 15-fold compared to the starting amount (food variant 2). To exclude RNAi inefficiency due to the refractoriness of the *βtub* target gene in our strain, we also included more positive control target genes, *fez2*, *lrc*, and *sem-1a*, from the yeast shRNA feeding publications [31,35]. The corresponding dsRNA sequences were designed to include the published siRNA target regions for these genes (see Table S2H). Moreover, additional targets, *acetylcholine esterase 1* (*ache1*) and *vacuolar-type ATPase* (*V-ATPase*), which showed potent lethal effects in other insects when knocked down by RNAi [62], were included. Despite the increased amount of bacteria per food volume, there was no significant difference in the larval survival rates between the *eGFP* control and any of the target genes (*p*-value = 0.324, one-way ANOVA, Welch’s test, Figure 2b).

**Figure 3:**
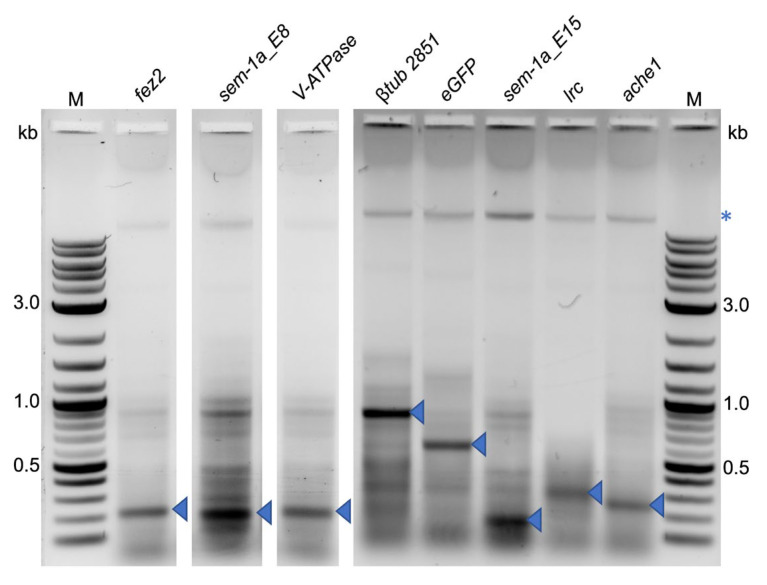
Gel electrophoresis of bacterially produced dsRNAs after phenol/chloroform/isoamyl alcohol extraction from bacterial cells [61]. Blue arrows indicate the expected size of the dsRNA. The blue asterisk indicates bacterial chromosomal DNA. M = 1 kb plus ladder (New England Biolabs Inc., Ipswich, MA, USA); kb = kilobases; for target gene name abbreviations, see Figure 2.

We extracted dsRNAs from the bacterial cells to exclude the lack of RNAi-induced mortality due to the lack of bacterial expression of the corresponding dsRNA. Gel electrophoresis revealed strong bands corresponding to the expected sizes for each dsRNA that was unique to the respective bacterial strain, indicating that all strains correctly expressed the dsRNAs (Figure 3).

#### 2.1.3. Further Enhancement of Bacteria Ingestion Does Not Improve the RNAi Effect

We hypothesized that fast larval growth, promoted by the high nutrient content in the bacterial food pellets used so far, limits the volume of food (i.e., bacteria) intake, thereby keeping the amount of ingested dsRNA below a biologically relevant threshold. If this is correct, a lower nutrient content in the bacterial feeding pellets could enhance the consumption of the bacterial cells, i.e., dsRNA. Moreover, a slower larval development would increase the dsRNA action time. We therefore decided to evaluate food formulations with a reduced nutrient content for RNAi efficiency. These experiments included an additional target gene, *coat protein (coatomer) alpha* (*αcop*), and bacteria transformed with the empty expression plasmid L4440 as an additional negative control.

Food variant 3 consisted of bacteria mixed with LB-agar, corresponding to a 5X bacterial dose per food volume. To support larval development towards the end of the experiment, the larvae were supplied with baker’s yeast after the end of the bacterial pellet feeding. We also performed a parallel feeding assay with fish food to obtain a standard for development time and survival rate under normal rearing conditions. The reduced nutrients resulted in a slower development time—from 13 days for the first pupation to up to 33 days until the last pupation or the death of the larvae. In contrast, the larvae fed with fish food had all pupated between 5 and 8 days and showed a >90% pupation rate. While an overall high lethality was observed in all feedings with the bacteria–LB-agar pellets, it could not be assigned to the dsRNA treatment (*p*-value = 0.355, one-way ANOVA, Welch’s test, Figure 2c).

With food variant 4, the last extra nutrients besides the bacteria, i.e., the salts from the LB medium, were omitted. Thus, the food consisted only of bacteria and agar. Moreover, the relative amount of bacteria per food volume was increased again to 15X. Two experiments with small differences in the feeding regime were performed (Table S3). In experiment 1, the larval age at feeding start was ~20 h; in experiment 2, the larvae were less than one hour old to test for the effect of very early exposure to the dsRNA.

In both experiments, we again observed delayed development (pupation onset 13–15 days after feeding start) and overall increased mortality, which was not different between the targets and the control dsRNAs *gusA* or *eGFP* (experiment 1: *p*-value = 0.017, one-way ANOVA, Welch’s test, Figure 2d; experiment 2: *p*-value = 0.612, one-way ANOVA, Figure 2e). The observed *p*-value < 0.05 for experiment 1 was due to differences between two of the treatment conditions (*lrc* and *ache1*, *p*-value = 0.042) and not to the control dsRNAs *gusA* and *eGFP* (Table S4, p. 31).

In a final iteration, we decided to follow the yeast feeding protocol, which uses dried tablets of pure yeast [38], and correspondingly produced pure bacterial tablets by drying *E. coli* pellets at 30 °C for 48 h. As these experimental conditions were prone to low water quality, the larvae were counted and transferred to clean water with a fresh bacterial tablet every 24 h for eight consecutive days, starting with approximately 20 h old L1 larvae. While 97.3 ± 2.1% (n = 200) of the larvae treated under standard rearing conditions pupated by the end of the eighth day, all larvae treated with the bacterial tablets were still in the larval stage. Starting from day 9, we added baking yeast to each container to support development. Under these conditions, pupation typically started after 14 days, and no significant difference was observed in the survival rates between the transcript-specific dsRNAs and the controls (*p*-value = 0.985, one-way ANOVA, Welch’s test, Figure 2f).

**Figure 4:**
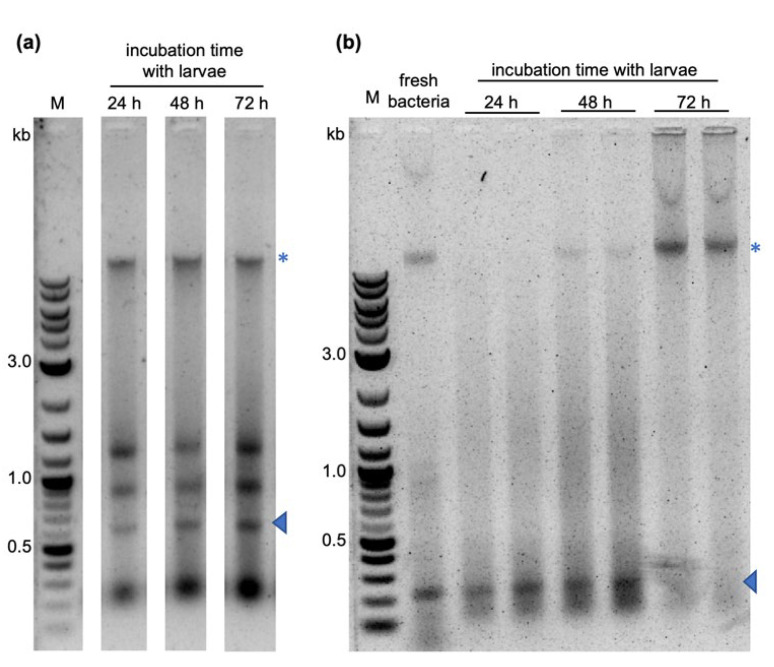
Gel electrophoresis of dsRNA recovered from bacteria–agar pellets (**a**) and pure dried bacteria tablets (**b**) after incubation in water with *Ae. aegypti* larvae. Each lane represents RNA extracted from one food pellet after incubation with 40 (**a**) or 20 (**b**) L1 to L2 larvae for the indicated periods; “fresh bacteria” represents dsRNA extracted from bacteria without larval incubation; blue arrows indicate the expected size of the dsRNA (*eGFP* in (**a**) and *fez2* in (**b**)). The blue asterisk indicates bacterial chromosomal DNA. M = 1 kb plus ladder (New England Biolabs Inc., Ipswich, MA, USA); kb = kilobases; for target gene name abbreviations, see Figure 2.

Thus, none of the dsRNA feeding strategies yielded larval mortality significantly different from that of the treatments with control RNAs. To exclude that the lack of the RNAi effect was caused by dsRNA degradation under the experimental conditions, we extracted dsRNA from the food pellets after 24, 48, or 72 h of incubation with mosquito larvae in water at 27 °C. Undegraded dsRNA could be recovered from the formulations with agar for up to 72 h of incubation with the larvae. In the feeding assays with the dried bacteria tablets, the dsRNA was stable for up to 48 h (Figure 4). Therefore, dsRNA degradation under the experimental conditions could be excluded as a probable cause for the absence of target gene-specific dsRNA-induced lethality.

### 2.2. Co-Delivery of shRNAs against Gut RNases Does Not Enhance Target Gene-Specific RNAi Effects

A major obstacle to the oral delivery of iRNAs in insects can be the presence of RNases in the gut, which degrade the biologically active RNA molecules before they can be taken up into the gut epithelial cells. One possible solution is the co-delivery of iRNAs targeting the RNase transcripts [64,65,66]. We therefore performed feeding assays with bacteria expressing shRNAs against *fez2* or *lrc* with or without gut RNase-specific shRNAs in two *Aedes* species, *Ae. aegypti* and *Ae. albopictus*. These experiments were conducted independently from the above feeding experiments in a different laboratory.

In *Ae. aegypti*, one nuclease gene was targeted, XM_001653429.2, while three putative gut nucleases were targeted in *Ae. albopictus*, XM_019679594.1 (referred to as “nuclease 1”), XM_019701402.1 (“nuclease 2”), and XM_019683641.1 (“nuclease 3”; for more detailed information, see Table S2). shRNA sequences against the target genes were the ones published previously (*Aae fez2* v2 and *Aae lrc* [31]) or designed de novo (*Aae fez2* v1, *Aae* nuclease, *Aal fez2*, and *Aal nucleases 1-3*). The shRNA design followed the information and loop sequence published by Mysore et al. [35]. The experiments were performed with the *Ae. aegypti* Liverpool and La Réunion strains and an *Ae. albopictus* strain collected in Montpellier, France. Control experiments were performed with bacteria that did not express shRNA (“none”) or that expressed an unspecific shRNA (“scramble”).

**Figure 5:**
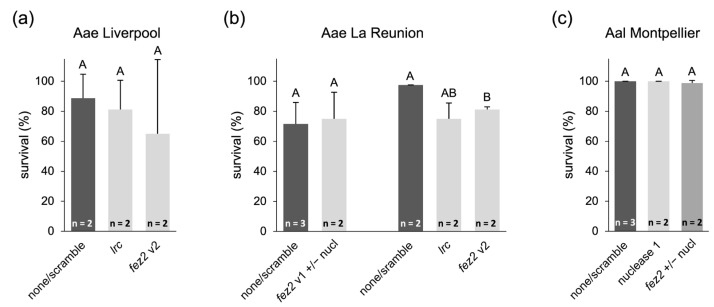
Survival rates of *Ae. albopictus* and *Ae. aegypti* following larval feeding with bacteria producing shRNAs against *fasciculation and elongation protein zeta2 (fez2)*, *leukocyte receptor cluster member 8 homolog* (*lrc)*, or nuclease-encoding transcripts (nucl). Each replicate contained 40 neonate larvae. Two types of negative controls were used: “none” means that no bacteria-expressed shRNA was added to the agar pellet; “scramble” means that bacteria expressing an unspecific shRNA were added to the agar pellet. Shown are average survival rates (in percent); error bars indicate standard deviation; different letters above the bars indicate statistically significant differences between gene-specific shRNA treatment and the unspecific control with *p*-value < 0.05 (one-way ANOVA, Welch’s test in (**a**), Student’s *t*-test and Welch’s *t*-test in (**b**), Student’s *t*-test in (**c**)); the numbers inside each bar indicate the number of replicates for each condition. Aae = *Ae. aegypti*, Aal = *Ae. albopictus; fez2* v1 is a newly designed shRNA sequence against *fez2*; *fez2* v2 and *lrc* correspond to the published siRNA sequences [31].

While not all conditions were tested in multiple replicates, these tests also lacked the previously reported strong lethal effect of *fez2* or *lrc* knockdown [31,35], and the presence of RNase-specific shRNAs did not improve the RNAi effect. Moreover, as also observed in the bacterial feeding assays described above, the treatment resulted in overall increased development times, and control survival numbers varied within and between experiments, partially due to limiting larvae feeding on the pellets (Table S5). For statistical analysis, the controls or the assays for the same target gene with or without RNase-targeting shRNA were combined (Figure 5). No significant lethal effects were observed in the feeding assays with the Liverpool strain (Figure 5a, *p*-value = 0.844, one-way ANOVA, Welch’s test), the La Réunion strain when targeting *fez2* v1 (Figure 5b, *p*-value = 0.828, Student’s *t*-test), or the *Ae. albopictus* Montpellier strain (Figure 5c, *p*-value = 0.272, Student’s *t*-test). Only when targeting *fez2* v2 in the La Réunion strain was a significant reduction in survival observed (Figure 5b, *p*-value = 0.0489, Welch’s *t*-test), but it was about five times lower than previously reported [31].

In another series of feeding experiments with the parallel knockdown of gut RNases, a study in *Ae. aegypti*, in which the knockdown of the female-specific isoform of *doublesex* (*dsx*) by RNAi caused a sex ratio distortion resulting in more than 90% male development [19], was replicated in *Ae. albopictus*. Here, two female-specific isoforms of the *dsx* transcript were targeted.

**Figure 6:**
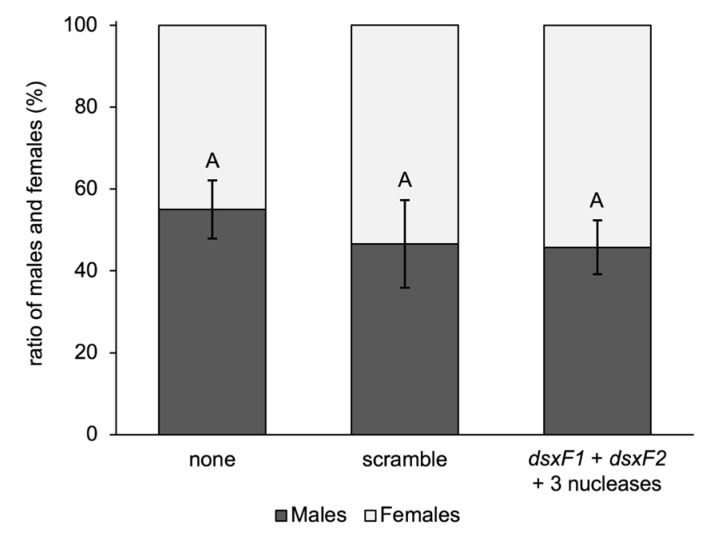
The sex ratio of *Ae. albopictus* adults (Montpellier strain) fed with bacteria producing shRNAs targeted against the two female-specific isoforms of *doublesex* (*dsxF1* and *dsxF2*). Two rounds of experiments were carried out, with 2–3 replicates for each condition. Each replicate contained 50 neonate larvae at the start of feeding. Two types of negative controls were used: “none” means that no bacteria-expressed shRNA was added to the agar pellet, and “scramble” means that bacteria expressing an unspecific shRNA were added to the agar pellet. The average male and female ratios (in percent) of both experiments combined are shown. Error bars indicate standard deviation; bars with common letters are not significantly different at 95% confidence level (one-way ANOVA).

*Ae. albopictus* larvae were fed a mixture of bacteria expressing shRNAs against the female-specific isoforms of *dsx* and all three RNase genes in multiple replicates. In some replicates (treatment and control), the agar pellets used for dsRNA feeding were barely consumed over time. Since larval development was much slower in such replicates, counting was stopped on day 30, meaning that it was not possible to determine the sex of the remaining underdeveloped larvae (Table S6), and the male–female ratio was evaluated based on the obtained pupae. The combined feeding of female *dsx*- and nuclease-targeting shRNAs did not result in any significant sex bias (*p*-value = 0.415, one-way ANOVA) in adult mosquitoes (Figure 6 and Table S6).

In summary, these feeding experiments independently replicated the above results and did not show an increased RNAi effect when co-feeding with shRNAs targeting gut RNases. Possibly, insufficient amounts of RNase-targeting shRNAs reached the gut epithelial cells, either because gut RNases also degraded them before cellular uptake, or because the uptake of the RNA into the gut epithelial cells or the release from the bacterial cells in the gut are limiting steps.

### 2.3. Soaking of Ae. aegypti Larvae in Concentrated dsRNA or siRNA Solutions Does Not Improve RNAi Efficiency

**Figure 7:**
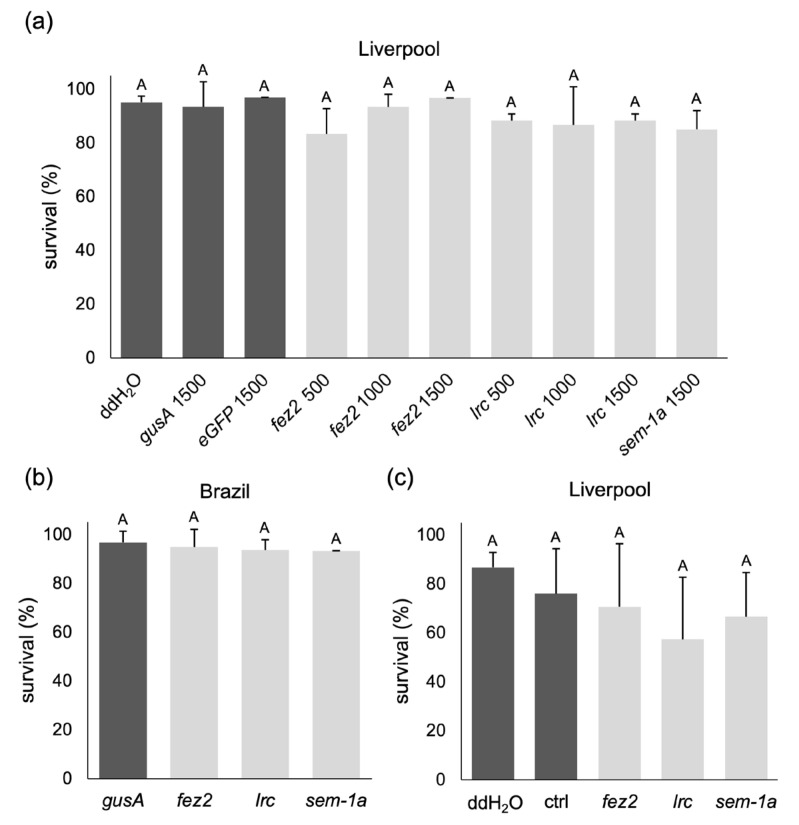
Survival rates after soaking *Ae. aegypti* early L1 larvae in concentrated dsRNA or siRNA solutions. (**a**) Liverpool wild-type strain larvae soaked in three different dsRNA concentrations, 500, 1000, and 1500 ng/µL; (**b**) Brazil wild-type strain larvae soaked in 1500 ng/µL dsRNA; (**c**) Liverpool larvae soaked in 1000 ng/µL siRNA solutions, with sequences from the literature [31,35]. Data in (**a**,**b**) are based on two biological replicates with 30 neonate L1 larvae each. Data in (**c**) are based on three biological replicates with 25 neonate L1 larvae each. Bars represent average survival rates in percent, error bars represent standard deviation, and bars with common letters are not significantly different at 95% confidence level (one-way ANOVA, Welch’s test in (**a**,**b**), one-way ANOVA in (**c**)). For abbreviations of target gene names, see Figure 2; ddH_2_O = double-distilled water; ctrl = unspecific siRNA sequence [31].

If the release of dsRNA molecules from the producing microbial cells was a limiting factor, the exposure of the larvae to concentrated dsRNA solutions could result in the desired effect. The so-called soaking, i.e., the incubation of mosquito larvae in concentrated dsRNA or siRNA solutions, has often been reported [19,34,35,36,41,43] and used, for example, to screen for potential RNAi targets [31]. We tested soaking with freshly hatched Liverpool L1 larvae (not older than 90 min) using concentrations from 500 to 1500 ng/µL of bacterially produced and extracted dsRNA targeting *fez2*, *lrc*, and *sem-1a_E8* for 4 h, and we subsequently monitored larval and pupal survival under standard rearing conditions. Control experiments used either only water, *gusA*, or *eGFP* dsRNA. All treatments, on average, yielded survival rates to pupal stage higher than 80% (Figure 7a), with no significant difference between treatments and the controls (*p*-value = 0.154, one-way ANOVA, Welch’s test).

The soaking experiment was repeated at the highest dsRNA concentration with an *Ae. aegypti* wild-type strain from Brazil to exclude a strain-specific lack of the RNAi effect (e.g., insensitivity to RNAi). Again, the survival rates for all treatments were at least 90% (Figure 7b), with no significant difference between the target gene treatments and the control targeting *gusA* (*p*-value = 0.857, one-way ANOVA, Welch’s test).

To test whether the absence of dsRNA-induced lethality was due to the failed processing of the dsRNA by the RNAi machinery after uptake into the gut cells, we next tried soaking the larvae in in vitro-synthesized siRNAs (Integrated DNA Technologies Inc., Coralville, IA, USA). The target and control siRNA sequences were taken from the literature [31,35], and an additional control with water was performed. Despite increasing the siRNA concentration by two- to four-fold compared to published assays, we did not observe any statistically significant differences in survival rates (Figure 7c, *p*-value = 0.223, one-way ANOVA).

**Figure 8:**
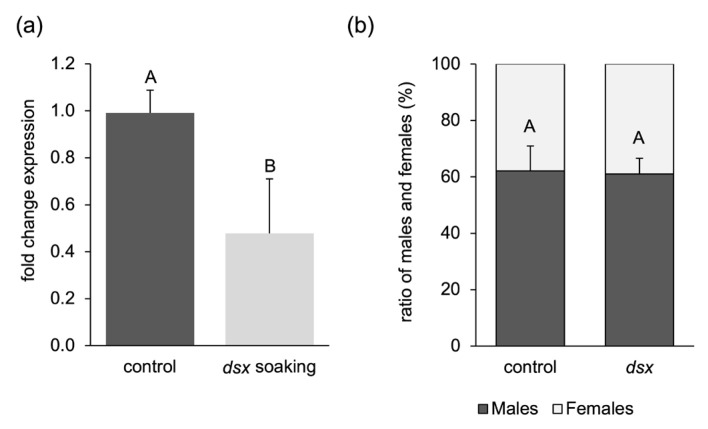
Targeting the female-specific transcript of the *doublesex* gene (*dsx*) by soaking *Ae. aegypti* larvae in *dsx*-dsRNA. Data are based on five to seven replicates with 50 to 100 neonate larvae each. Shown is the average fold change in *dsx* transcript levels after repeated soaking of larvae in *dsx*-dsRNA compared to the control (=*GFP*-dsRNA) in (**a**), as determined by RT-qPCR and calculated with the ΔΔCt method [67]; 8 × 3 larvae were pooled and analyzed for the control, and 11 × 3 larvae for the *dsx*-dsRNA. Panel (**b**) shows the male-to-female ratio (in percent) of adult mosquitoes after larval soaking in *dsx* or control (=*GFP*) dsRNA (**b**). Error bars represent standard deviation, and different letters above the bars indicate statistically significant differences with *p*-value < 0.05 (Welch’s *t*-test in (**a**), Student’s *t*-test in (**b**)).

Independent dsRNA soaking experiments were performed in another laboratory, again targeting the female-specific transcript of *dsx*. While exposure to the *dsx* dsRNA resulted in about a 50% reduction in the levels of the female-specific *dsx* transcript (Figure 8a, *p*-value = 0.0000334, Welch’s *t*-test), there was no difference in the male-to-female ratio between the control and *dsx* dsRNA treatments (Figure 8b, *p*-value = 0.815, Student’s *t*-test), which is in contrast to the published sex bias [19]. Overall, the lack of an RNAi response in the soaking experiments indicates that releasing the RNA molecules from the producing cells is not the limiting step. This still leaves multiple reasons for the failure to produce an RNAi response, including degradation by gut RNases, failed uptake into the gut epithelial cells, or the inability of the RNAi machinery to process the dsRNAs or shRNAs.

### 2.4. siRNA or dsRNA Injections of Ae. aegypti Larvae Do Not Lead to Gene Knockdown

**Figure 9:**
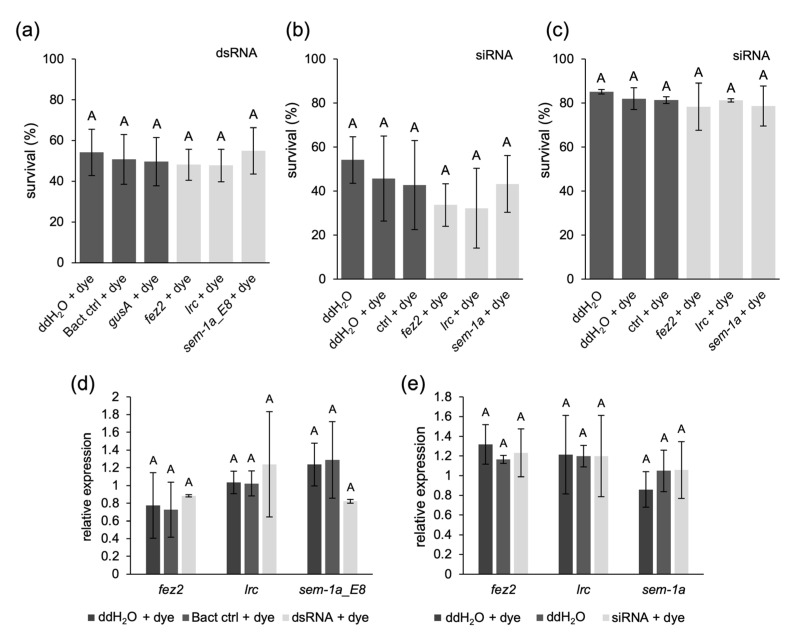
Effects of siRNA and dsRNA injections into *Ae. aegypti* L2 or L4 larvae. Average survival rates (in percent) to pupal stage (**a**–**c**) and average target gene transcript levels 24 h after injection (**d**,**e**) are shown. (**a**) dsRNA injections into L2 larvae; (**b**) siRNA injections into L2 larvae and (**c**) into L4 larvae; the *sem-1a* target sequence corresponds to the one published in [35]; data shown in (**a**–**c**) are based on three biological replicates, with 100 individuals per replicate. Three larvae in (**d**) and five larvae in (**e**) were pooled for RNA extraction and RT-qPCR 24 h post-injection. Relative expression was calculated following the Pfaffl method [57], using *rps17* as a reference gene. Data shown are based on two technical replicates from two biological replicates. For abbreviations of target gene names, see Figure 2; “dye” is food color mixed with ddH_2_O or with siRNA or dsRNA solutions to visualize the success of injection, and “bact ctrl” is total RNA extracted from bacteria transformed only with the empty expression vector L4440. Error bars indicate standard deviation, and bars with common letters are not significantly different at a 95% confidence level (one-way ANOVA in (**a**–**c**), and one-way ANOVA and Welch’s test in (**d**,**e**)).

Delivering dsRNA by injection into larvae circumvents the gut RNases and a possible gut epithelial barrier. Injection of siRNAs would avoid issues with the intracellular processing of the dsRNAs. We started with injections of the bacterially produced and extracted anti-*fez2*, -*lrc*, and -*sem-1a* dsRNAs into L2 larvae. dsRNA solutions were mixed with a food dye to track the injection and ensure the uniformity of the injected volume. The *E. coli*-specific *gusA* dsRNA, total RNA extract from bacteria transformed with the empty L4440 expression plasmid (“bact ctrl”), and water with food dye served as negative controls. One replicate without dye was also included to verify the non-toxicity of the food dye. After injection, the larvae were allowed to recover, moved to normal rearing conditions, and monitored for pupation rate. No significant lethality was observed in the injections with target dsRNAs compared to the controls (*p*-value = 0.499, one-way ANOVA, Figure 9a). To assess the mRNA levels of the targeted genes, pools of three to five larvae from each injection were sampled 24 h after the injection and analyzed by RT-qPCR. However, also at the transcript level, no effect of the dsRNA injections could be observed (*fez2*: *p*-value = 0.824, *lrc*: *p*-value = 0.917, and *sem-1a_E8*: *p*-value = 0.337, one-way ANOVA, Welch’s test, Figure 9d).

To assess the possible bottleneck of dsRNA processing, we injected siRNAs into L2 and L4 larvae, using the same siRNA sequences as for the soaking experiments. Water, water plus food dye, and an unspecific siRNA were used as controls. Neither in L2 nor in L4 larvae did we observe significant lethality with the target gene siRNAs (Figure 9b,c; L2 larvae: *p*-value = 0.127, L4 larvae: *p*-value = 0.363, one-way ANOVA). The general lower survival rates of the L2 larvae compared to L4 larvae are likely due to increased sensitivity to the injection procedure at a younger age. As the expression of *fez2* and *lrc* was detected in the fourth larval instar brain [31], we assessed target gene transcript levels by RT-qPCR in L4 as described above. We did not detect a significant reduction compared to the control injections at the transcript level (Figure 9e; *fez2*: *p*-value = 0.719, *lrc: p*-value = 0.999, *sem-1a*: *p*-value = 0.690, one-way ANOVA, Welch’s test).

### 2.5. Embryonic Injection with eGFP dsRNA Reduces eGFP mRNA and Protein Levels in a Transgenic Line

**Figure 10:**
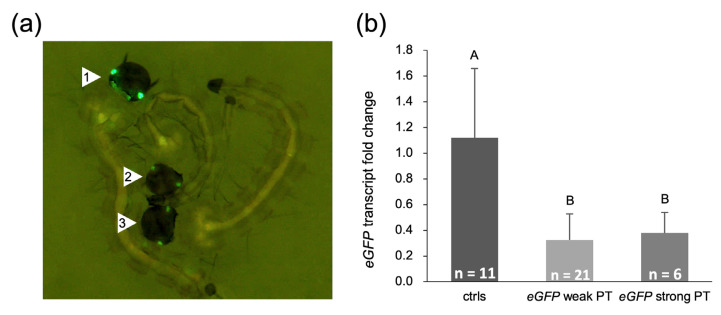
Targeting of *eGFP* transcript in early *Ae. aegypti* transgenic embryos via *eGFP* dsRNA injection. (**a**) Exemplary fluorescence image of larvae injected with bacterial extract as control (animal 1) or dsRNA targeting the *eGFP* transcript (animals 2, 3). (**b**) Relative mRNA levels were assessed in larvae injected with an extract from bacteria transformed with the empty expression plasmid or with ddH_2_O (ctrls), or with bacterially produced *eGFP* dsRNA (*eGFP* weak PT = weak phenotype and *eGFP* strong PT = stronger phenotype). Shown is the average fold change in transcript levels for the respective groups measured by RT-qPCR and calculated with the ΔΔCt method [67]. For the bacterial extract control, five individuals from a total of 20 injection survivors from two independent experiments were analyzed; for the ddH_2_O control, six out of seven injection survivors; and for the *eGFP* dsRNA, 24 out of 32 survivors from two independent injections; n = number of analyzed individuals (also see Table S7). Error bars indicate standard deviation, and different letters above the bars indicate statistically significant differences with *p*-value < 0.05 (one-way ANOVA, Welch’s test).

We finally evaluated the RNAi sensitivity in very early *Ae. aegypti* embryos. For this, preblastodermal embryos of an *eGFP*-expressing transgenic line were injected with bacterially produced and extracted *eGFP* dsRNA (same sequence as used in the experiments described above as a negative control) in two independent experiments (Table S7). Injection survivors were screened in the late L2 to L4 larval stages for *eGFP* fluorescence intensity compared to individuals injected with water or total RNA extract from wild-type bacterial cells. While all injection survivors from the control injections showed bright *eGFP* expression in the eyes, most *eGFP*-injected individuals showed a clearly reduced, sometimes very weak *eGFP* fluorescence intensity (Figure 10a). After fluorescence screening, each individual was quickly frozen at −80 °C for total RNA extraction and RT-qPCR analysis of *eGFP* transcript levels. RT-qPCR confirmed a significant downregulation of *eGFP* transcript levels in the individuals with weak eGFP expression, but also in the individuals with a stronger eGFP phenotype (Figure 10b, *p*-value = 0.002, one-way ANOVA, Welch’s test). These results show that the bacterially produced dsRNA is biologically active.

## 3. Discussion

Gene knockdown by RNAi has been used to study gene function in mosquitoes and has gained a lot of attention regarding the development of RNAi-based vector control. For the success of this approach, the RNAi response should be robust and insensitive to potential external and internal variables. When we, the authors, independently of each other, started to set up RNAi as a tool in our laboratories, we chose protocols and target sequences reported to result in a high larval lethality or sex bias [19,31,35,36] for assay establishment. Surprisingly, however, we collectively failed to reproduce the published effects, although these well-designed studies provided exact information on the iRNA structure and sequences and detailed protocols for producing and delivering the iRNAs.

Several factors are critical for the successful induction of an RNAi response, including the stability of the RNA molecules in the environment and during delivery, the uptake from the gut lumen and intracellular release, the processing of the iRNA by the RNAi machinery, and the induction of systemic RNAi [4,17,18,56,62,68]. To troubleshoot the reasons for our failure to reproduce the published results, we independently started to test different RNAi-triggering molecules (siRNA, shRNA, and dsRNA) in combination with different delivery pathways (oral delivery via iRNA-producing microorganisms provided to the larvae in various formulations, soaking in pure iRNA solutions, and microinjections). None of the different combined strategies resulted in strong or consistent phenotypic effects. This also applied to the respective target gene transcript levels. Only in two assays could a consistent effect be observed: (1) *eGFP* knockdown in embryos of a transgenic line by *eGFP*-dsRNA injections, resulting in significant transcript level reductions combined with a clearly reduced fluorescence phenotype, and (2) knockdown of the female-specific *dsx* transcript in *Ae. aegypti* (approximately 50% across all replicates), but without the published sex bias phenotype [19]. Otherwise, only moderate reductions occurred randomly in single experiments or at a specific sampled stage.

Five different *Ae. aegypti* laboratory strains were used in total for all the assays performed in the three labs, which excludes the possibility of RNAi resistance in a specific strain.

An often-described bottleneck in oral RNAi is the presence of RNases in the insect gut that potentially degrade the iRNAs before they can be taken up by the gut epithelial cells [18,56]. One solution is the co-delivery of iRNA(s) that target the RNase transcripts [64,65,66]. In our experiments, however, the oral delivery of RNase-targeting shRNAs, together with shRNAs against *fez2*, *lrc*, or *dsx*, did not result in a target-specific phenotype in *Ae. aegypti* or *Ae. albopictus*.

The delivery of the iRNA molecules by microorganisms would also protect the RNAs from the nucleases during passage through the gut. However, if RNAi is to be triggered via the ingestion of iRNA-producing microorganisms, these need to be lysed in the gut to set the iRNA free. Coon et al. [69] reported that bacterial cells are required for normal larval development and can be later found dead in the gut of the mosquito larvae. We therefore assume that at least part of the bacterially produced dsRNA was released into the gut lumen upon death of the microorganisms. However, the amount could have been below a biologically meaningful threshold. This would be supported by observations made by Romoli et al. [68], who could not detect siRNA enrichment in the tissues of *Ae. aegypti* females whose gut had been colonized by dsRNA-producing bacteria. Attempts to detect siRNA enrichment following oral administration of heat-killed *E. coli* also yielded no positive results. However, upon oral administration of naked dsRNA or dsRNA injection into adult females, siRNAs were enriched, indicating the delivery and uptake of sufficient amounts with these strategies, but not with the microorganisms.

To address the possibility that the microorganisms’ iRNA molecules were not expressed or degraded in the formulated food pellets during the assay, we purified dsRNAs from fresh cells or from food pellets after extended exposure to larval feeding, showing that dsRNAs were produced and stable. However, it is impossible to compare our RNA yield to that of other studies, as, overall, the amount of iRNA produced by and released from microorganisms in the published assays is a black box. Therefore, there could be a strong inherent variance between the used iRNA-producing strains, which could be a major factor in the observed lack of reproducibility.

Another possibility for the lack of RNAi effects is the failed processing of the delivered dsRNA into siRNAs by the cellular RNAi machinery. While we did not experimentally assess this step, the assays described above from Romoli et al. [68] show the processing of dsRNAs by the RNAi machinery. Based on this, we would have expected better RNAi efficiency when delivering naked dsRNA or chemically synthesized siRNAs against *fez2*, *lrc*, and *sem-1a*, via larval soaking or larval injections, using the published highly successful target sequences. However, except for the approximately 50% *dsx* transcript level knockdown after the soaking of larvae in dsRNA (see above), none of the experiments resulted in a measurable phenotypic or transcript level effect. Conversely, McFarlane et al. [70] showed that, despite successful in vitro siRNA-mediated gene silencing in *Ae. aegypti*-derived Aag2 cells, the same effect was not observed upon injection of *Ae. aegypti* female adults with siRNAs. However, strong knockdown was observed when the mosquitoes were injected with dsRNA targeting the same gene.

So far, the mechanisms involved in RNAi in mosquitoes have been barely studied and need to be better understood to be able to explain the observed variances. The effect of RNases in the gut and hemolymph remains a major mystery in mosquito RNAi. While dsRNA could be reextracted from mosquitoes at least in part up to two days post-oral delivery [25,68] and up to 7 days post-injection [71], the incubation of dsRNA with serial dilutions of gut juice led to the fast degradation of the dsRNA (Figure S2). Also, the pathway of dsRNA upon ingestion or injection is barely known. Fluorescently labeled dsRNA could be detected in some gut epithelial cells after oral delivery, but was not followed further [68]. Labeled dsRNA injected into females was found enriched mainly in hemocytes and ovaries [71], but could not be detected after oral administration. On the contrary, many solid studies show high RNAi efficiency upon the oral administration of naked iRNAs [31,33,43,72,73].

Other studies have tested different nanomaterials for the packaging of dsRNA and the effect on the extent of gene silencing upon oral administration of such nanoparticles. Nanoparticles provide protection from gut RNases and are also used for the delivery of therapeutics to target cells in medical research. In several studies in *Aedes*, the RNAi effect was stronger when using packaged compared to naked dsRNA. Interestingly, the extent of gene silencing was dependent on the nanomaterial used [45,47,51]. But also here, the results are not consistent, and the effects can be gene-specific [47]. The potential of nanoparticles for RNAi-based insect management, including different nanomaterials, nanoparticle formulations, and their effect on different insect species, has recently been reviewed elsewhere [74,75,76]. One should consider that RNAi technology, when incorporating additional components formulated with the RNA, might be subject to additional regulatory frameworks akin to those for chemicals, potentially losing the regulatory advantages typically associated with RNA-only biologicals.

The apparent limitations of RNAi are not unique to mosquitoes and have been observed in several species over the past decade. The research performed so far clearly shows that not all insects are equally susceptible to dsRNA and/or RNAi in general. While Coleopterans are considered very RNAi-susceptible, Hemipterans show varying effects, and Dipterans and Lepidopterans show overall low RNAi efficiencies. The Coleopteran *Tribolium castaneum* is the model insect for RNAi, in which a comprehensive gene function study by systematic RNAi-mediated gene knockdown has been performed [77]. Even parental RNAi works very well in these beetles. In contrast, lepidopteran species are often considered RNA recalcitrant. One of the main reasons for that seems to be the presence of dsRNases in the saliva or the midgut, which quickly digest the ingested dsRNA in recalcitrant but not in susceptible species [78,79,80,81,82,83]. 

Overall, the studies performed over the past 10–15 years have also helped to uncover several barriers to RNAi in insects besides gut RNases, such as dsRNA uptake into cells, intracellular release, or the spread of the RNAi signal in the insect body. A lot of our understanding of the RNAi mechanism and the involved genes and processes comes from model organisms such as *C. elegans* or from human studies. Core RNAi enzymes like Dicer and Ago2 have been found in many insects. However, other mechanisms and factors involved in the RNAi process are less conserved and seem to differ between orders, families, species, and even tissues or stages. Thus, there is not “one mechanism of insect RNAi” but many varying factors, which probably contribute to the high variability in RNAi effects across and within insect species. It is beyond the scope of this manuscript to go into further mechanistic details, but current knowledge has been summarized comprehensively before [4,5,18,56]. However, it is essential to further investigate the molecular mechanisms of RNAi across different species.

For *Aedes* mosquitoes, the inconsistent and sometimes contradictory RNAi effects in the literature, in combination with our failed attempts to reproduce published data independently in three independent laboratories, strongly suggest a high complexity of the RNAi mechanisms in *Aedes* mosquitoes and of the factors influencing these mechanisms, which are both insufficiently understood and produce unexpected and currently unexplainable variability in experiments. It also shows that RNAi application in *Aedes* mosquitoes is less robust and straightforward than the successful studies imply. This is important information that needs to be shared to raise awareness and open the discussion about the challenges involved, with the goal to further study and improve RNAi in *Aedes* species.

## 4. Materials and Methods

The data presented in this study were generated in three different laboratories independently and without knowledge of each other; therefore, the methods presented in this section are subdivided by laboratory. All primer and interfering RNA sequences used in this study are provided in the Supplementary Material (Table S2).

### 4.1. Protocols from Department of Insect Biotechnology in Plant Protection, Justus Liebig University Giessen, Germany

#### 4.1.1. Mosquito Rearing

*Ae. aegypti* wild-type strains and the transgenic line V19 were reared in an insectary at 27 °C with 70% relative humidity (RH) and a 12:12 h light–dark cycle. Larvae were fed on Tetra TabiMin fish food pellets (Tetra GmbH, Melle, Germany), and adults were fed on sterile-filtered 10% (w/v) sucrose solution. Adult females were fed once per week with pig blood purchased from a butcher shop.

The *Ae. aegypti* laboratory strains used in the following experiments were the Orlando (collected from Orlando, FL, USA, in 1952) [84], Liverpool (the Liverpool reference strain, originating from West Africa in 1936) [85,86,87,88], and Brazil (collected in Juazeiro, Carnaiba do Sertão, Brazil, between 2012 and 2014) wild-type strains.

The transgenic *Ae. aegypti* line V19 contains one copy of the *piggyBac* construct pB_*attp*_*3xP3-eGFP_attPrev.* When homozygous, it shows strong *eGFP* expression in the eyes and in the ventral nerve cord, which has been published previously [89].

#### 4.1.2. Cloning of dsRNA Expression Vectors for Bacterial Expression

The dsRNA sequences for the *beta-tubulin* (*βtub*) genes AAEL002851 and AAEL004939, as well as for the *E. coli beta-glucuronidase* (*gusA*) control, were identical to the ones published [19,36], using the primer sequences provided in these publications, with overhangs for ApaI-NotI cloning. PCRs were performed on *Ae. aegypti* complementary DNA (cDNA) and *E. coli* XL1-Blue genomic DNA (gDNA) with Platinum Tag polymerase (Invitrogen, Waltham, MA, USA). A 20 µL reaction contained 1X Platinum Taq buffer, 2.5 mM MgCl_2_, 200 µM dNTPs, 500 nM forward and reverse primers, and the DNA template. The cycling conditions were 1× 95 °C 90 s, 30× 94 °C 30 s, 56 °C 20 s, 72 °C 1 min/1 kb, and 1× 72 °C 5 min. Products were digested with ApaI and NotI (25 µL PCR reaction, 4 µL CutSmart, 10–20 U enzyme, double-distilled water (ddH_2_O) to 40 µL; 1.5 h, 37 °C), and gel was purified with the Zymoclean Gel DNA Recovery Kit (Zymo Research Europe GmbH, Freiburg, Germany) following the manufacturer’s instructions, but elution was performed with a pre-heated buffer (60 °C), and T4 DNase-ligated (New England BioLabs, Inc., Ipswich, MA, USA) into the ApaI-NotI-digested and gel-purified L4440 expression plasmid (Addgene plasmid # 1654) at 16 °C overnight.

The dsRNA sequences for *acetylcholine esterase 1* (*ache1*, LOC5578456), *vacuolar-type ATPase* (*V-ATPase*, LOC5575718, AAEL012035), *fasciculation and elongation protein zeta-2* (*fez2*, LOC5569012, AAEL007292), *leukocyte receptor cluster member 8 homolog* (*lrc*, LOC5569340, AAEL007548), *semaphorin-1a* (*sem-1a*, LOC5575438, AAEL002653) exon 8 and exon 15, and *coat protein (coatomer) alpha* (αcop) (LOC5577214, AAEL015001) were designed with the online tool eRNAi [90] using the following parameters: |siRNA length for specificity prediction: 17 bp, exclude low complexity regions, exclude > 5× CA[ACGT] repeats, siRNA seed length: 6, efficiency scoring: weighted, minimal siRNA efficiency score: 40, homology e-value cut-off: 1 × 10^−5^, amplicon size range: 150–500|. Only sequences producing zero off-target siRNAs in the *Ae. aegypti* genome were used. For *fez2*, *lrc*, and *sem-1a*, the dsRNA sequences suggested by eRNAi, which contained the published siRNA sequences [31,35,63], were chosen. The primers for *ache1*, *V-ATPase*, *fez2*, *lrc*, and *sem-1a* were designed with overhang for KpnI-SacI restriction cloning into the L4440 plasmid. The primers for αcop were designed with overhang for Gibson Assembly cloning (New England Biolabs, Inc., Ipswich, MA, USA) into the KpnI-EcoRI-digested L4440 plasmid. All PCRs were performed on genomic DNA (gDNA) with Phusion Flash High-Fidelity polymerase (Thermo Fisher Scientific Inc., Waltham, MA, USA). A 20 µL reaction contained 100 ng gDNA, 250 nM forward and reverse primers, and 1X Phusion Flash Master mix. Reactions were performed as 1× 98 °C 10 s, 30× 98 °C 1s, 54 °C 5 s, 72 °C 15 s, 1× 72 °C 1 min. PCR products were gel-purified and extracted with a Zymoclean Gel DNA Recovery Kit (Zymo Research). *ache1*, *V-ATPase*, *fez2*, *lrc*, and *sem-1a* PCR products were digested with KpnI and SacI (50 µL rxn with 20 U each enzyme, 1X CutSmart buffer and the complete purified PCR product) and ligated with the KpnI-SacI-digested plasmid L4440 (2 µg plasmid, 20 U each enzyme, 1X CutSmart buffer in 50 µL) using T4 DNA ligase overnight at 16 °C. The αcop PCR product was cloned into the KpnI-EcoRI-digested L4440 plasmid following the manufacturer’s Gibson Assembly cloning protocol. All ligated plasmids were first transformed into XL1-Blue cells (*E. coli* [recA1 endA1 gyrA96 thi-1 hsdR17 supE44 relA1 lac [F ´ proAB lacI^q^Z∆M15 Tn10 (Tet^r^)]]; Agilent Technologies, Santa Clara, CA, USA) for sequence verification and, after that, into HT115 DE3 (*E. coli* [F-, mcrA, mcrB, IN(rrnD-rrnE)1, rnc14::Tn10(DE3 lysogen: lacUV5 promoter -T7 polymerase]; Caenorhabditis Genetics Center) cells, followed again by plasmid extraction and sequencing.

#### 4.1.3. In Vitro Transcription (IVT) of *βtub* and *gusA* dsRNA

IVT templates were produced by PCR on the respective L4440 plasmid using Q5 High-Fidelity polymerase (New England BioLabs Inc., Ipswich, MA, USA) and the gene-specific primers with a 5′ extension for the T7 promoter adapter. A 50 µL reaction contained 1X Q5 buffer, 100 nM forward and reverse primers, 200 µM dNTPs, and 0.5 µL Q5 polymerase. The cycling conditions were 1× 98 °C 30 s, 30× 98 °C 10 s, 65 °C 20 s, 72 °C 60 s, and 1× 72 °C 2 min. The PCR product was purified by gel electrophoresis and extraction with the Zymoclean Gel DNA Recovery Kit (Zymo Research Europe GmbH, Freiburg, Germany).

IVT was performed using the MEGAscript kit (Ambion/Life Technologies, Thermo Fischer Scientific Inc., Waltham, MA, USA) according to the manufacturer’s instructions, using 500 ng of the PCR product as the template. Reactions were incubated for 4 h at 37 °C. Then, an annealing step was performed: 75 °C for 5 min and cooling down to room temperature, followed by DNase digest at 37 °C for 15 min. The dsRNA was purified using the MEGAclear Transcription clean-up kit (Ambion/Life Technologies, Thermo Fischer Scientific) according to the manufacturer’s instructions. The dsRNA was eluted with 50 µL elution solution incubated on a column at 65 °C for 5 min. The elution was performed twice. The dsRNA quality was analyzed by agarose gel electrophoresis.

#### 4.1.4. Cloning of shRNA Expression Vectors for Expression in the Yeast Strain BY4742

shRNA sequences were deducted from the literature for *fez2* and *lrc* [31] and for *sem-1a* [35]. Sequences were ordered as DNA oligo templates from IDT (Integrated DNA Technologies, Inc., Coralville, IA, USA) containing a 5′ overhang with a BamHI restriction site and a 3′ overhang with an XhoI restriction site for cloning into the pRS426 YE shuttle vector (plasmid #77107, American Type Culture Collection, ATCC).

shRNA templates were amplified using the Phi29 primer extension method [91]. A 20 µL reaction contained 20 pmol oligo template, 20 pmol 3′ primer ATCTCCATGCAGCTCGAG, 1X Phi29 reaction buffer, 8 µg BSA, 50 mM dNTPs, and 10 U Phi29 DNA polymerase (New England BioLabs Inc., Ipswich, MA, USA). The reaction was incubated for 10 min at 30 °C, followed by heat inactivation of the enzyme for 10 min at 65 °C. The gel-purified PCR products (Zymoclean Gel DNA Recovery Kit, Zymo Research Europe GmbH, Freiburg, Germany) were subcloned into the Zero Blunt TOPO vector (Invitrogen, Waltham, MA, USA) following the manufacturer’s instructions and transformed into XL1-Blue cells. Colonies with the correct cloning product were identified by plasmid preparation (NucleoSpin Plasmid Mini kit, Macherey-Nagel GmbH & Co. KG, Düren, Germany) and sequencing. The shRNA sequence was then cut out of the correct plasmids by restriction digest (100 ng plasmid DNA, 20 U XhoI and BamHI (New England BioLabs), 1X CutSmart buffer in 25 µL total volume, 1 h at 37 °C), purified via gel electrophoresis, and ligated into the BamHI- and XhoI-digested and gel-purified pRS426 vector using T4 DNA ligase (New England BioLabs) at 16 °C overnight, followed by transformation of the ligated products into XL1-Blue cells. Colonies with the correct cloning product were again identified by plasmid preparation (NucleoSpin Plasmid Mini kit, Macherey-Nagel) and sequencing and then transformed into the BY4742 *S. cerevisiae* strain following the protocol from Mysore et al. [38] step by step. To confirm correct plasmids, single colonies were grown in 3 mL YPD medium for 24 h at 30 °C, and DNA was extracted using a NucleoSpin Plasmid Mini kit (Macherey-Nagel) with one adaptation of the protocol: cells from 2 mL yeast culture were pelleted and resuspended in 250 µL Buffer A1, transferred to a tube with ceramic beads, and homogenized (Precellys^®^, Bertin Instruments, Montigny-le-Bretonneux, France) at 6000 rpm for 20 s. Then, the protocol was continued according to the manufacturer’s instructions. Plasmid sequence was verified by sequencing.

#### 4.1.5. Culturing of dsRNA-Expressing Bacteria

Bacteria were grown at 37 °C and 150 rpm in LB medium containing 12.5 ng/µL tetracycline (Fisher BioReagents, Geel, Belgium) and 100 ng/µL ampicillin (Carl Roth GmbH + Co. KG, Karlsruhe, Germany) to an optical density per mL measured at a wavelength of 600 nm (OD_600_/mL) of 0.4 to 0.8. dsRNA expression was induced by the addition of 0.4 mM IPTG. Cells were grown for another 4 h at 37 °C and 150 rpm, harvested, and further treated according to the different food preparation protocols.

#### 4.1.6. Feeding of *Ae. aegypti* L1 Larvae with dsRNA-Expressing Bacteria

The dsRNA-expressing HT115 DE3 cells used for these feeding experiments were grown and induced for dsRNA production as described above.

Variant 1: The composition of food pellets was based on the information from Whyard et al. [19]. As no exact amounts were indicated, the following amounts were mixed as starting conditions: bacterial pellet from 100 mL culture grown to an OD_600_/mL of 0.7–1.0, 5 mL of LB-agar cooled to 45–50 °C, and 1.25 g of ground TabiMin fish food (Tetra GmbH, Melle, Germany). After mixing well by pipetting, the solution was aliquoted in ca. 100 µL droplets in a Petri dish, cooled down, and stored at 4 °C in a sealed dish if not used immediately. This food composition contained the equivalent of 20 mL bacterial culture per 1 mL of food = 1X. The amount of bacterial cells was further increased to 50 mL and 100 mL bacterial culture per 1 mL of food (= 2.5X and 5X, respectively).

L1 larvae of the Orlando wild-type strain (ca. 15 h old) were counted in 12 groups of 10 larvae per target, incubated in 6-well plates in ddH_2_O, and fed ad libitum with the food pellets. Larvae were supplied with ca. 50 µL food pellet in the first four to five days of the experiment. Then, feeding increased, with 100 µL food pellet per feeding. Water was exchanged as needed. If food or water needed to be replaced in one well, then it was also replaced in all other wells. The feeding amount across all wells was always consistent. Every individuum that pupated was counted as a survivor.

Variant 2: Bacterial cells from 50 mL culture harvested at OD_600_ of 1.4–1.6 were mixed with 12 mg of finely ground Tetra TabiMin fish food. Then, 55 µL of 6% (w/v) pre-melted agarose solution was added, and the mix was thoroughly homogenized using a pipette tip. Bacterial pellets were allowed to solidify at room temperature for at least 10 min and transferred to 4 °C for at least 20 min before use for larvae feeding, or they were stored at −20 °C for later use within one week. Each pellet was used as one feeding portion to 20 L1 larvae in 50 mL of autoclaved double-distilled water. Fresh feeding portions were provided as needed or, at the latest, every 48 h. This food composition provided the equivalent of 300 mL culture/mL of food (=15X).

Variant 3: Bacterial cells from 400 mL culture were resuspended in 4 mL of pre-melted LB-agar, together with ampicillin (final concentration 100 µg/mL) and tetracycline (final concentration 12.5 µg/mL). This corresponded to the equivalent of 100 mL culture/1 mL of food pellet (=5X). The solution was transferred to a 5 mL syringe with a cut-off tip, allowed to solidify, and slices of 0.5 mL were used to feed 40 neonate larvae in 50 mL autoclaved ddH_2_O. Fresh feeding portions were provided as needed or, at the latest, every 48 h. To support larval development after the end of the bacterial feeding, baker’s yeast was added to the water to a final concentration of 0.08 mg/mL.

Variant 4: Bacterial cells from 50 mL of culture harvested at OD_600_/mL of 1.6–2.2 were mixed with 55–110 µL of 2–6% (w/v) of pre-melted agarose (see Table S3 for specific amounts) in 1.5 mL microfuge tubes. Bacterial pellets were left to cool down and solidify at room temperature for at least 10 min and transferred to 4 °C for at least 20 min before use for larvae feeding, or they were stored at −20 °C for later use within one week. To support larval development towards the end of the bacterial feeding, 12 mg of finely ground fish food was added to the food pellets (Table S3). Each pellet was used as one feeding portion to 20 neonate or 20 h old L1 larvae in 50 mL of autoclaved ddH_2_O. Pellets were replaced when consumed or, at the latest, after 3 days.

Variant 5: Similar to the production of dried tablets of shRNA-expressing yeast [38], 50 mL of bacterial cells cultured and harvested as above was collected in 1.5 mL microfuge tubes and dried at 30 °C and 50% RH for 48 h. Dried pellets were used immediately for feeding assays or stored for up to one week at −20 °C. Each pellet was used as one feeding portion for 40–60 L1 larvae in 50 mL of autoclaved ddH_2_O and replaced daily, including water change. After 8 days, baking yeast was provided at 0.08 mg/mL as only source of nutrition.

#### 4.1.7. Feeding of *Ae. aegypti* L1 Larvae with shRNA-Expressing Yeast

For the preparation of the yeast cultures and dried yeast pellets, as well as the feeding procedure of the *Ae. aegypti* larvae, we closely followed the protocol by Mysore et al. [38] with some small changes: larvae were supplied with yeast on the first day of the experiment (pellet corresponding to 50 mL culture grown to OD_600_/mL of 2.5–3) and again on the fourth day of the experiment, including a water change. In cases where transcript levels were assessed by RT-qPCR, the experiment started with 50 L1 larvae in 50 mL ddH_2_O, and 2 × 5 larvae were sampled from each cup on day 3 and day 5 of the experiment, as well as 2 × 5 pupae, and stored at −80 °C immediately. The amount of yeast added per mL of ddH_2_O was not adjusted, as the water remained cloudy from the yeast, so there was never a shortage in yeast cells, even with the increased number of larvae. Each biological replicate was performed with 2–3 technical replicates.

#### 4.1.8. Soaking of Neonate *Ae. aegypti* Larvae with dsRNA or siRNA

The dsRNAs used in these experiments were extracted from HT115 DE3 cells as previously described [61]. siRNAs were purchased from IDT (Integrated DNA Technologies, Inc., Coralville, IA USA). dsRNAs or siRNAs were diluted in ddH_2_O to 500 ng/µL, 1000 ng/µL, or 1500 ng/µL. *Ae. aegypti* larvae either less than 90 min old or between 20 and 24 h old were gently transferred to 1.5 mL microfuge tubes containing 25 to 100 µL of either the dsRNA or siRNA solutions. Soaked larvae were checked under a microscope to ensure complete immersion. After 4 h of soaking, larvae were gently transferred to autoclaved ddH_2_O and further reared under regular rearing conditions. Development was monitored daily until pupation. Soaking assays were performed with 20 to 30 larvae per replicate.

#### 4.1.9. RNA Extraction and RT-qPCR of Larvae/Pupae Sampled from Yeast shRNA Feeding Assays

Total RNA was extracted from samples stored at −80 °C with a Monarch total RNA Miniprep kit (New England Biolabs Inc., Ipswich, MA, USA) or TRIzol™ Reagent (Invitrogen, Waltham, MA, USA) following the manufacturer’s instructions. Then, 0.5–1 µg of RNA was reverse-transcribed using a QuantiNova Reverse Transcription Kit (QIAGEN, Venlo, The Netherlands) according to the kit’s protocol. The kit uses an integrated gDNA removal step and a mix of oligo-dT and random primers. cDNA was diluted either 1:5 or 1:6 in ddH_2_O, depending on the expected level of gene expression, and 2 µL was used per qPCR reaction. qPCR was performed with the SsoAdvanced Universal SYBR Green Supermix (Bio-Rad, Hercules, CA, USA) in a 10 µL reaction volume with 2 technical replicates for each condition. qPCR primers were evaluated for primer efficiency and primer dimer formation. The reference gene was the ribosomal protein S17 (*rps17*, AAEL004175) [92]. Changes in transcript levels relative to those of the control treatments were calculated using Pfaffl’s mathematical model [57], which incorporates an efficiency correction to account for the real-time PCR efficiency of the individual transcripts with the following formula:

$$ratio=\frac{{\left(E_{target}\right)}^{{\Delta CP}_{target}(control-sample)}}{{\left(E_{ref}\right)}^{{\Delta CP}_{ref}(control-sample)}}$$

Here, “*ratio*” is the relative expression ratio, *E_target_* is the real-time PCR efficiency of the target gene transcript, *E_ref_*is the real-time PCR efficiency of the reference gene transcript, Δ*CP_target_* is the CP difference of the control to the sample of the target gene transcript, and Δ*CP_ref_* is the CP difference of the control to the sample of the reference gene transcript. The ratio was first calculated separately for each experimental replicate by normalizing the target gene CP for each replicate to the reference gene CP averaged across all replicates per treatment or control. Then, the ratios of all experimental replicates of one target gene or control treatment were averaged, and the standard deviation was calculated.

#### 4.1.10. Larval Injections with siRNA or dsRNA Solutions

Following standard rearing conditions, larvae of *Ae. aegypti* at two different development stages (L2 or early L4) were randomly selected to undergo microinjections. Animals were injected in the section between the thorax and abdomen, taking care to avoid the gut. Needles for injection were prepared using siliconized quartz glass capillaries (Science Products for Research in Life Science GmbH, Product Number Q100-70-7.5, Hofheim, Germany) pulled in a P-2000 laser puller (Sutter Instruments, Novato, CA, USA). The injection setup consisted of an MN-151 micromanipulator (Narishige, Tokyo, Japan), a FemtoJet 4i (Eppendorf, Hamburg, Germany), and a SZX16 stereo microscope (Olympus, Tokyo, Japan). The dsRNAs used in these experiments were extracted from the HT115 DE3 cells as previously described [61]. siRNAs were purchased from IDT (Integrated DNA Technologies, Inc., Coralville, IA USA). Larvae were injected with solutions of siRNA or dsRNA at 1 µg/µL in three biological replicates with 100 individuals each. Food dye was mixed into the injection solution to allow for the tracking of the injected volume and ensure the uniformity of the injections throughout the experiments. The food dye was verified to not degrade siRNA or dsRNA during the time of the injection. After the injection, larvae were allowed to recover shortly, gently transferred to normal rearing conditions, and monitored daily. RNA extraction and RT-qPCR of larvae sampled 24 h after injection was performed as described above for larvae and pupae sampled from yeast shRNA feeding assays.

#### 4.1.11. Embryonic Microinjections with *eGFP* dsRNA

Preblastodermal embryos of the V19 *eGFP*-expressing transgenic line (pB_attp_3xP3-eGFP_attprev) of *Ae. aegypti* [89] were collected by allowing females homozygous for the single-copy transgene construct to oviposit for 30 min. Eggs were then rowed on a wet filter paper to ensure that all were in the same anterior–posterior orientation, transferred to double-sided sticky tape on a cover slide, and covered with halocarbon oil 27 (Sigma Aldrich/Merck KG, Darmstadt, Germany). Embryos were injected between 60 and 120 min post-oviposition start into the posterior pole with *eGFP* dsRNA produced by and extracted from *E. coli* HT115 DE3 cells [61] and dissolved in ddH_2_O at 1 µg/µL. Control injections were performed with ddH_2_O or with total RNA extracted from wild-type HT115 DE3 cells not expressing any dsRNA. Surviving G_0_ individuals were reared to the L2 to L4 larval stage under standard rearing conditions and assessed for *eGFP* expression level by fluorescence microscopy. Larvae were then quick-frozen individually at −80 °C for later RNA and DNA extraction using the TRIzol^TM^ method (see above). *eGFP* mRNA levels were measured by RT-qPCR for each larva individually as described above, and the fold change in transcript levels was calculated by the ΔΔCt method [67]. The fold change was first calculated separately for each experimental replicate by normalizing the target gene CP of each replicate to the reference gene (= *rps17*) CP averaged across all replicates per treatment or control. Then, the fold changes in all experimental replicates of one target gene or control treatment were averaged, and the standard deviation was calculated. Moreover, the transgene copy number of each larva was confirmed to be two by digital droplet PCR according to the protocol in [89].

### 4.2. Protocols from ASTRE, CIRAD, Montpellier, France

#### 4.2.1. Mosquito Rearing

The mosquito strains used for the following experiments were the *Ae. aegypti* La Réunion wild-type strain, collected from La Réunion Island, France, in or before 2018; the Liverpool strain; and an *Ae. albopictus* wild-type strain collected in Montpellier, France. The *Ae. albopictus* strain was reared in a climatic chamber at 25 °C and 70% RH, and *Ae. aegypti* strains were reared at 27 °C and 70% RH. Both light cycles were 14 h light–10 h dark with dawn and dusk. Other rearing conditions were essentially as described above.

#### 4.2.2. shRNA Cloning for Bacterial Expression

Novel shRNA sequences were designed with the RNA interference tool from IDT: https://www.idtdna.com/site/order/designtool/index/DSIRNA_PREDESIGN (accessed on 25 February 2019). Obtained sequence suggestions were checked with Blast for off-targets. Every sequence that produced more than 17 bp off-target hits was excluded. Oligo primers were designed to represent the shRNA target sequence and the reverse complement linked by the loop sequence (5′AAGTTCTCT3′). An ‘AATG’ overhang was added to the 5′ end and ‘TGAG’ to the 3′ end of the top strand. The forward oligo started with the 5′ overhang (AATG) and ended at the end of the reverse complement. The reverse oligo started with the 3′ overhang (=CTCA, i.e., the reverse complement of TGAG) and ended at the start of the shRNA target sequence. The oligos were phosphorylated with polynucleotide kinase (New England BioLabs Inc., Ipswich, MA, USA) in a 10 µL reaction containing 1X PNK buffer, 1 µL PNK, and 100 µmol oligo for 1 h at 37 °C. Oligo pairs were mixed, the volume was increased to 200 µL, and oligos were annealed in a thermal cycler: 96 °C 6 min, 96 °C–1 °C per cycle down to 23 °C. Annealed oligos were blunt-cloned into the PJet1.2/blunt cloning vector (Thermo Fisher Scientific Inc., Waltham, MA, USA) and transformed into *E. coli* DH5alpha cells. Colonies with the correct sequence plasmid were identified by colony PCR. Plasmids were purified (E.Z.N.A. Plasmid DNA Mini Kit I, Omega Bio-tek, Inc., Norcross, GA, USA) and transformed into HT115 DE3 cells, and resulting colonies screened again for correct plasmid sequence by colony PCR. Positive plasmids were confirmed by sequencing.

#### 4.2.3. Culturing of shRNA-Expressing Bacteria and Preparation of Food Pellets

HT115 DE3 cells were grown as described above, and shRNA expression was induced at 0.8 OD_600_/mL. Food pellets were prepared by pelleting cells from 40 mL culture, resuspending the cells in 4 mL of 1% (w/v) agar at 80 °C, and adding 1 mL brewer’s yeast slurry. The mixture was incubated for 15 min at 80 °C to heat-kill the bacteria and filled into a 5 mL syringe with a cut-off tip. Upon solidifying, the food was cut into 0.5 mL sections.

#### 4.2.4. Feeding of *Ae. aegypti* and *Ae. albopictus* Larvae with shRNA-Expressing Bacteria

Forty first-instar larvae of *Ae. aegypti* or *Ae. albopictus* were kept in a Petri dish with 25 mL of ddH_2_O, supplied with two 0.5 mL food pellets, and incubated at 28 °C for 4–6 days until the fourth instar.

### 4.3. Protocols from Department of Microbiology and Molecular Genetics, Michigan State University, East Lansing, USA

#### 4.3.1. Mosquito Rearing

Mosquito rearing conditions were essentially as described above. The *Ae. aegypti* wild-type strain Waco (originally collected from the field in Waco Texas and has been colonized in the lab for >20 years [93]) was used for the following soaking experiments.

#### 4.3.2. *Ae. aegypti Doublesex* and *GFP* dsRNA Synthesis by IVT

For dsRNA synthesis by IVT, total RNA was extracted from fourth-instar larvae and reverse-transcribed to cDNA as described below. cDNA was used as a template for PCR. Primers with a T7 promoter sequence added to their 5′ end were employed for amplification. The PCR product was purified using a NucleoSpin Gel and PCR Clean-up kit (Takara Bio Inc., San Jose, CA, USA) after cutting the PCR product bands from gel analysis. Subsequently, dsRNAs were synthesized using the T7 RiboMAX^TM^ Express RNAi System (Promega Corp., Madison, WI, USA). As a control, ds*GFP* was also synthesized for larval RNAi experiments.

#### 4.3.3. *Ae. aegypti* Larval Soaking in *dsx* dsRNA

Purified dsRNA was dissolved to a concentration of 1000 ng/μL. Approximately 50 to 100 newly hatched larvae were transferred into a 50 mL conical tube, with its bottom replaced with a sieve to enable soaking. Subsequently, all the larvae were soaked in dsRNA at a concentration of 1000 ng/μL in soaking buffer for 2 h per day over 6 days. *dsGFP* was used as the control, with at least five replicates for each treatment. After soaking, the larvae were returned to a cup containing fresh water and food and allowed to recover for 24 h at 27 °C and 80% RH. Mosquito sexes were determined based on the morphological character of the adults developed after treatment. For RNAi efficiency determination via target gene transcript levels, larvae were collected in TRIzol^TM^ solution after the last soaking with 3 larvae/pool.

#### 4.3.4. Total RNA Extraction and RT-qPCR of *dsx* dsRNA-Treated *Ae. aegypti* Larvae

Insect tissues were collected in 1.5 mL tubes and immediately flash-frozen in liquid nitrogen. Total RNA was then extracted using TRIzol^TM^ reagent (Invitrogen, Waltham, MA, USA) following the manufacturer’s instructions. cDNA was synthesized from purified total RNA using a QuantiTect Reverse Transcription Kit (QIAGEN, Venlo, The Netherlands; integrated gDNA removal step and mix of oligo-dT and random primers). Subsequently, real-time quantitative PCR (RT-qPCR) was performed for 40 cycles on the QuantStudio Real-Time PCR System (Applied Biosystems/Thermo Fischer Scientific Inc., Waltham, MA, USA) using SYBR™ Green PCR Master Mix (Thermo Fischer Scientific Inc., Waltham, MA, USA). *rps6* was used as a reference gene. Transcript levels of the target mRNA were normalized to reference gene levels within the same samples. Each experiment involved the collection of four separate samples, with duplicate measurements conducted for each. The relative gene expression was calculated as rq = 2^−ΔΔCt^ [67].

### 4.4. Statistical Analysis

Data analysis was carried out using the software SigmaPlot (Version 14.0, Systat Software Inc., San José, CA, USA) and MiniTab^®^ (Minitab, LLC., State College, CA, USA). Data were analyzed via a one-way analysis of variance (ANOVA), with the Shapiro–Wilk test for normality and the Brown–Forsythe test for equal variance. The Holm–Sidak method was used for multiple comparisons of the data from each condition, or the Bonferroni test was performed for comparison with a control group when the differences in the mean values among the groups were significant (α = 0.05). Data that failed the Shapiro–Wilk test for normality underwent an arcsin or Box-Cox transformation [94] prior to conducting the ANOVA. When equal variance could not be assumed, the data were analyzed via Welch’s one-way ANOVA. The means were then compared with the Games–Howell pairwise comparison at a 95% confidence level. Student’s *t*-test or Welch’s *t*-test was used to compare the means between two groups when equal variances were or were not assumed, respectively. One sample *t*-test was performed to compare observed means to a hypothesized value. The Wilcoxon signed rank test was performed for non-parametric data. All statistical analyses are provided in Table S4.

## 5. Conclusions

In summary, the results of this study, produced independently in different laboratories, in combination with other RNAi results published to date, demonstrate inconsistent effects, indicating a high variability in RNAi efficiency in *Aedes* mosquitoes, for which the reasons are currently not understood. For the reproducible and reliable application of RNAi in (*Aedes*) mosquitoes, we need to better understand the involved molecular mechanisms and contributing factors, which will require further basic research on the different steps of the RNAi process and the fate of RNAi-triggering molecules inside the mosquito body. Only then will we be able to develop RNAi protocols that are robust, not only under laboratory conditions but also in the field, where varying climatic conditions and other variables such as the larval development stage, nutrition status, or availability of alternative nutrition sources influence the system. Only if the system is stable enough to be unaffected by these variables can the technology reliably be used for mosquito control or sexing within genetic control programs.

## Data Availability

The original contributions presented in the study are included in the article/Supplementary Materials; further inquiries can be directed to the corresponding author/s.

## Supplementary Materials

The following supporting information can be downloaded at: https://www.mdpi.com/article/10.3390/ijms25105218/s1.
