# Supplementary material for: Challenges of Robust RNAi-Mediated Gene Silencing in Aedes Mosquitoes
Source: Int J Mol Sci. 2024 May 10;25(10):5218. doi: 10.3390/ijms25105218 (PMC11121262; doi:10.3390/ijms25105218)
Supplement: Supplementary file 1 [file ijms-25-05218-s001.zip › Figure S1.pdf]

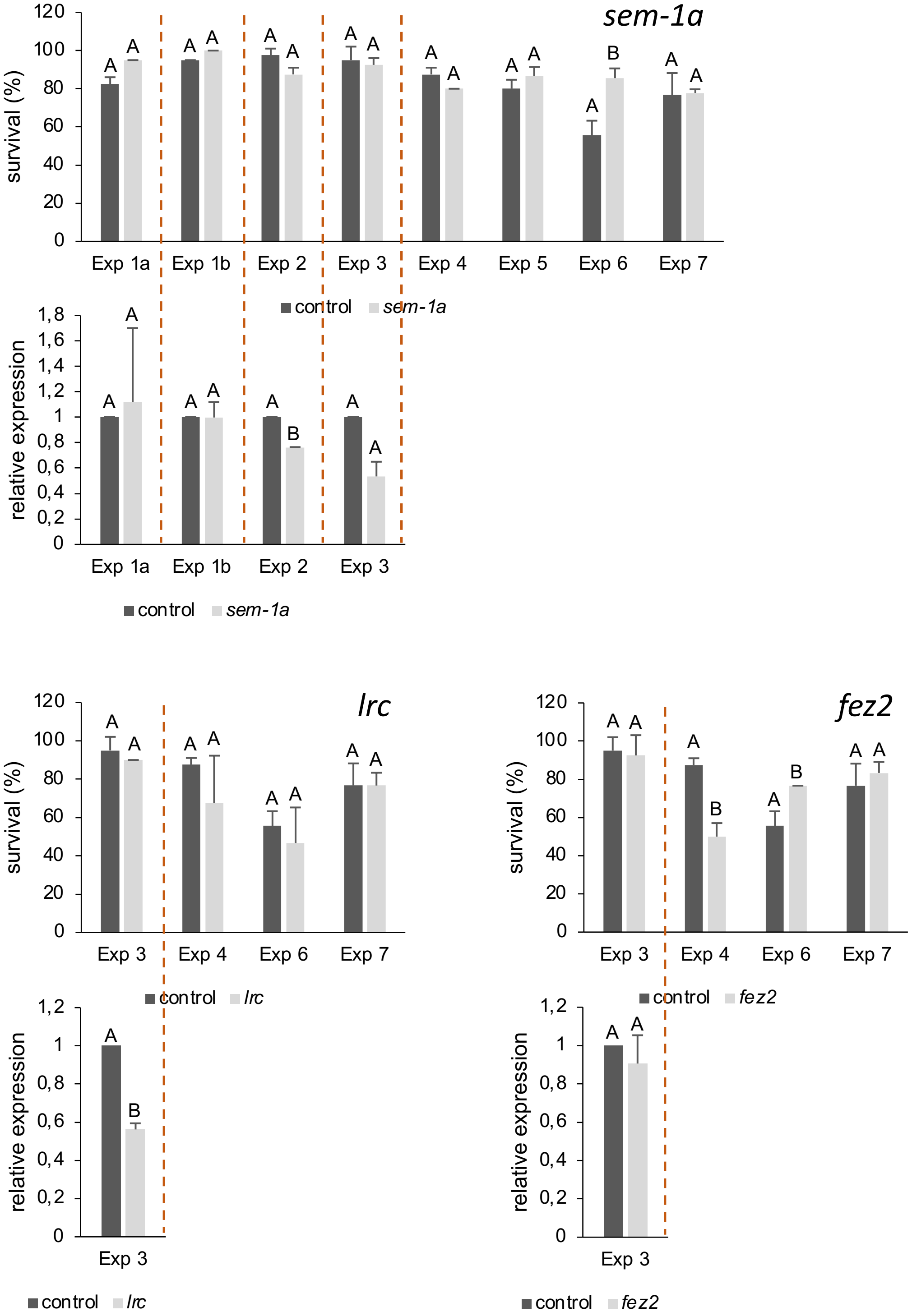

Figure S1: Percent survival until pupation of *Ae. aegypti* larvae fed with shRNA-expressing yeast, and corresponding transcript levels of targeted genes in the surviving pupae. Shown are the average survival rates of the different feeding experiments with shRNA-producing yeasts that are summrized in Figure 1 of the study. Insects were monitored until pupation and all viable pupae were counted as survivors. In case transcript levels were assessed by RT-qPCR, five pupae were pooled. Relative expression was calculated with the Pfaffl method. Bars and error bars represent the average survival and standard deviation, respectively, of two technical replicates for experiments 1 to 5, and three replicates for experiments 6 and 7. Each replicate contained 20 (exp. 1-4) to 30 (exp. 5-7) individuals. Exp. 1-4 and 6 were performed with larvae of the Orlando wild type strain, exp. 5 and 7 with the Liverpool wild type strain. Exp. 1a and 1b were performed with yeast from the same culture batch, but on different days. Relative transcript levels of experiments 6 and 7 are shown in Figure 1. “Control” is the feeding with yeasts expressing an unspecific shRNA); “Exp” = independent experiment (i.e. biological replicate); *sem-1a = semaphorin-1a*, *lcr = leukocyte receptor cluster*, *fez2 = fasciculation and elongation protein zeta2;* Raw data are provided in Table S1
