# Supplementary material for: Challenges of Robust RNAi-Mediated Gene Silencing in Aedes Mosquitoes
Source: Int J Mol Sci. 2024 May 10;25(10):5218. doi: 10.3390/ijms25105218 (PMC11121262; doi:10.3390/ijms25105218)
Supplement: Supplementary file 1 [file ijms-25-05218-s001.zip › Figure S2.pdf]

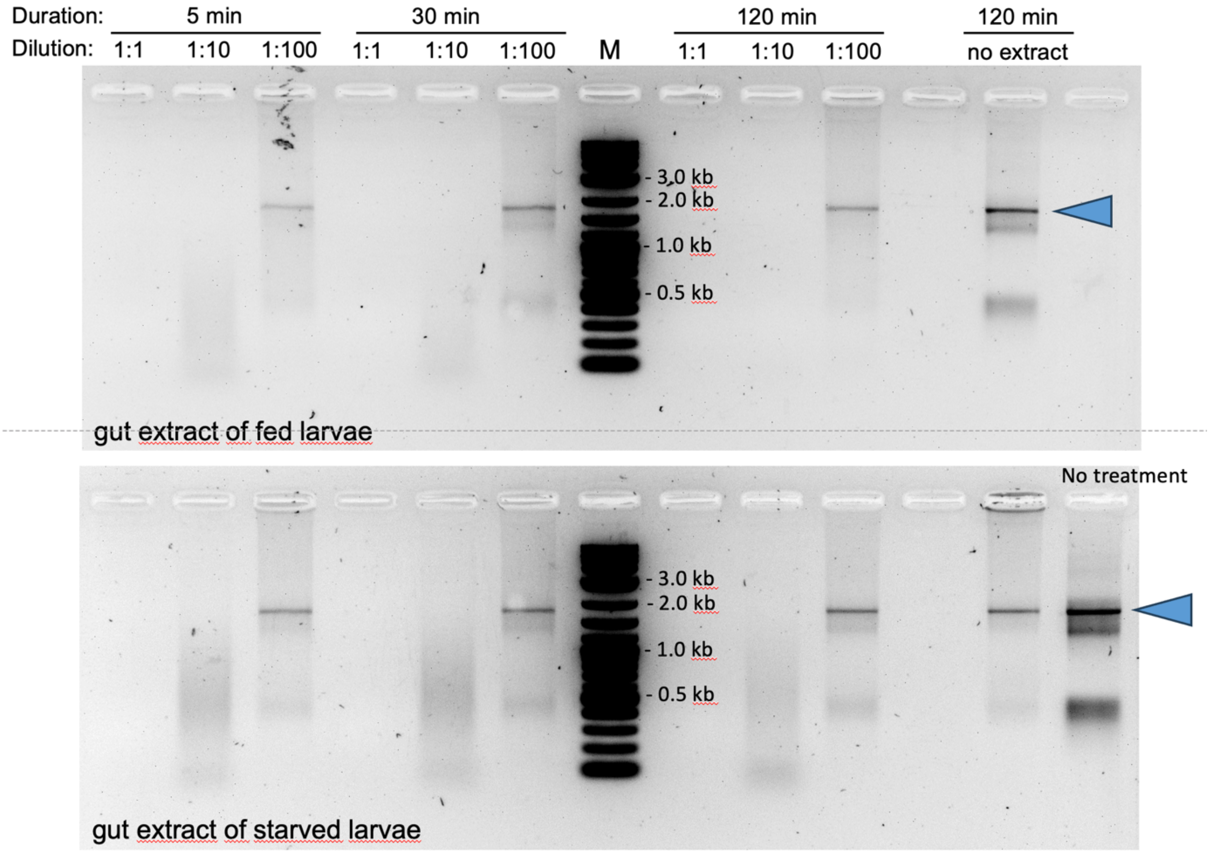

Figure S2. Gel electrophoretic analysis of dsRNA incubated with a serial dilution of *Ae. aegypti* gut extract. Guts of ten L3/L4 larvae (fed on fish food or starved for 24 h) were pooled in RNase-free 1x TE buffer and homogenized with a pistil. The supernatant was serially diluted and incubated as indicated with 200 ng of in vitro transcribed *gusA* dsRNA. The digestion was stopped by proteinase K digest before gel analysis. Arrows indicate the position of the undigested *gusA* dsRNA (size: ~1.8 kb). M = 1 kb plus ladder (NEB).
