# Supplementary material for: Challenges of Robust RNAi-Mediated Gene Silencing in Aedes Mosquitoes
Source: Int J Mol Sci. 2024 May 10;25(10):5218. doi: 10.3390/ijms25105218 (PMC11121262; doi:10.3390/ijms25105218)
Supplement: Supplementary file 1 [file ijms-25-05218-s001.zip › Table S3.pdf]

Table S3. Feeding scheme for bacteria-agar pellets in Orlando, food variant 4. As the first day pellet in experiment 1 was not consumed at all, the day 4 pellet was produced with less agar to facilitate the young larvae feeding. In experiment 2, the agar concentration of the first food pellet was reduced to 2% to account for the putatively low strength in the mouth parts of the very young larvae. “Agar content” specifies the concentration (w/v) and the volume of the agar solution used to prepare the food pellets.

Experiment 1			Experiment 2		
Feeding day	Agar content	Fish food	Feeding day	Agar content	Fish food
1	6% (w/v), 110 µL	-	1	2 % (w/v), 110 µL	-
4	4% (w/v), 110 µL	-	4	6 % (w/v), 110 µL	-
6	6% (w/v), 55 µL	-	6	6 % (w/v), 110 µL	-
8	6% (w/v), 55 µL	-	8	6 % (w/v), 110 µL	-
10	6% (w/v), 55 µL	-	10	6 % (w/v), 110 µL	-
11	6% (w/v), 55 µL	yes	11	6 % (w/v), 110 µL	yes
			13	6 % (w/v), 110 µL	yes
			15	6 % (w/v), 110 µL	yes
