# Supplementary material for: Challenges of Robust RNAi-Mediated Gene Silencing in Aedes Mosquitoes
Source: Int J Mol Sci. 2024 May 10;25(10):5218. doi: 10.3390/ijms25105218 (PMC11121262; doi:10.3390/ijms25105218)
Supplement: Supplementary file 1 [file ijms-25-05218-s001.zip › Table S4.pdf]

Figure 1a

One Way Analysis of Variance

Normality Test (Shapiro-Wilk): Failed (P < 0,050)

Equal Variance Test (Brown-Forsythe): Passed (P = 0,066)

Group Name	N	Missing	Mean	Std Dev	SEM
Control	18	0	81,759	15,151	3,571
sem1a	18	0	87,407	7,632	1,799
fez2	10	0	76,500	15,937	5,040
lrc	10	0	68,500	20,942	6,622

Source of Variation	DF	SS	MS	F	P
Between Groups	3	2484,346	828,115	3,871	0,014
Residual	52	11125,525	213,952		
Total	55	13609,871			

The differences in the mean values among the treatment groups are greater than would be expected by chance; there is a statistically significant difference (P = 0,014).

Power of performed test with alpha = 0,050: 0,653

One Way Analysis of Variance

Normality Test (Shapiro-Wilk): Passed (P = 0,806)

Equal Variance Test (Brown-Forsythe): Passed (P = 0,480)

Group Name	N	Missing	Mean	Std Dev	SEM
asinsqrt(control)	18	0	1,172	0,223	0,0526
asinsqrt(sem1a)	18	0	1,236	0,153	0,0360
asinsqrt(fez2)	10	0	1,098	0,224	0,0710
asinsqrt(lrc)	10	0	0,991	0,233	0,0736

Source of Variation	DF	SS	MS	F	P
Between Groups	3	0,422	0,141	3,350	0,026
Residual	52	2,185	0,0420		
Total	55	2,608			

The differences in the mean values among the treatment groups are greater than would be expected by chance; there is a statistically significant difference (P = 0,026).

Power of performed test with alpha = 0,050: 0,558

Multiple Comparisons versus Control Group (Bonferroni t-test):

Comparisons for factor:

Comparison	Diff of Means	t	P	P<0,050
asinsqrt(con vs. asinsqrt(lrc	0,181	2,238	0,088	No
asinsqrt(con vs. asinsqrt(fez	0,0748	0,925	1,000	Do Not Test
asinsqrt(con vs. asinsqrt(sem	0,0641	0,939	1,000	Do Not Test

A result of "Do Not Test" occurs for a comparison when no significant difference is found between two means that enclose that comparison. For example, if you had four means sorted in order, and found no difference between means 4 vs. 2, then you would not test 4 vs. 3 and 3 vs. 2, but still test 4 vs. 1 and 3 vs. 1 (4 vs. 3 and 3 vs. 2 are enclosed by 4 vs. 2: 4 3 2 1). Note that not testing the enclosed means is a procedural rule, and a result of Do Not Test should be treated as if there is no significant difference between the means, even though one may appear to exist.

Figure 1B

One-Sample t-test

Normality Test (Shapiro-Wilk): Passed (P = 0,624)

Group Name	N	Missing	Mean	Std Dev	SEM
4_Sem1a_1, day 3	3	0	1,074	0,241	0,139

Hypothesized population mean 1,000

t = 0,534 with 2 degrees of freedom.

95 percent two-tailed confidence interval for the population mean: 0,475 to 1,674

Two-tailed P-value = 0,647

The difference between the mean of the sampled population and the hypothesized population mean is not great enough to reject the hypothesis that the difference is only due to random sample variability. There is not a significant difference between the two means (P = 0,647).

One-tailed P-value = 0,323

The sample mean of the group does not exceed the hypothesized mean by an amount great enough to exclude the possibility that the difference is due to random sampling variability. The hypothesis that the hypothesized mean is greater than or equal to the true mean cannot be rejected. (P = 0,323).

One-Sample t-test

Normality Test (Kolmogorov-Smirnov): Passed (P = 0,481)

Group Name	N	Missing	Mean	Std Dev	SEM
16_Sem1a_1, day 5	2	0	0,582	0,0131	0,00926

Hypothesized population mean 1,000

t = -45,118 with 1 degrees of freedom.

95 percent two-tailed confidence interval for the population mean: 0,464 to 0,700

Two-tailed P-value = 0,0141

There is a statistically significant difference between the mean of the sampled population and the hypothesized population mean (P = 0,014).

One-tailed P-value = 0,00705

The hypothesized mean exceeds the sample mean of the group by an amount that is greater than would be expected by chance, rejecting the hypothesis that the true mean of group is greater than or equal to the hypothesized mean. (P = 0,007).

One-Sample t-test

Normality Test (Shapiro-Wilk): Passed (P = 0,111)

Group Name	N	Missing	Mean	Std Dev	SEM
28_Sem1a_1,, pupae	3	0	0,944	0,278	0,160

Hypothesized population mean 1,000

t = -0,351 with 2 degrees of freedom.

95 percent two-tailed confidence interval for the population mean: 0,253 to 1,634

Two-tailed P-value = 0,759

The difference between the mean of the sampled population and the hypothesized population mean is not great enough to reject the hypothesis that the difference is only due to random sample variability. There is not a significant difference between the two means (P = 0,759).

One-tailed P-value = 0,380

The hypothesized mean does not exceed the sample mean of the group by an amount great enough to exclude the possibility that the difference is due to random sampling variability. The hypothesis that the true mean is greater than or equal to the hypothesized mean cannot be rejected. (P = 0,380).

Figure 1C

One-Sample t-test

Normality Test (Shapiro-Wilk): Passed (P = 0,214)

Group Name	N	Missing	Mean	Std Dev	SEM
4_Sem1a_1, day 3	3	0	0,942	0,0879	0,0507

Hypothesized population mean 1,000

t = -1,137 with 2 degrees of freedom.

95 percent two-tailed confidence interval for the population mean: 0,724 to 1,161

Two-tailed P-value = 0,373

The difference between the mean of the sampled population and the hypothesized population mean is not great enough to reject the hypothesis that the difference is only due to random sample variability. **There is not a significant difference between the two means (P = 0,373).**

One-tailed P-value = 0,187

The hypothesized mean does not exceed the sample mean of the group by an amount great enough to exclude the possibility that the difference is due to random sampling variability. The hypothesis that the true mean is greater than or equal to the hypothesized mean cannot be rejected. (P = 0,187).

One-Sample t-test

Normality Test (Shapiro-Wilk): Passed (P = 0,973)

Group Name	N	Missing	Mean	Std Dev	SEM
16_Sem1a_1, day 5	3	0	1,013	0,148	0,0856

Hypothesized population mean 1,000

t = 0,151 with 2 degrees of freedom.

95 percent two-tailed confidence interval for the population mean: 0,645 to 1,381

Two-tailed P-value = 0,894

The difference between the mean of the sampled population and the hypothesized population mean is not great enough to reject the hypothesis that the difference is only due to random sample variability. There is not a significant difference between the two means (P = 0,894).

One-tailed P-value = 0,447

The sample mean of the group does not exceed the hypothesized mean by an amount great enough to exclude the possibility that the difference is due to random sampling variability. The hypothesis that the hypothesized mean is greater than or equal to the true mean cannot be rejected. (P = 0,447).

One-Sample t-test

Normality Test (Shapiro-Wilk): Passed (P = 0,959)

**Group Name**	**N**	**Missing**	**Mean**	**Std Dev**	**SEM**
28_Sem1a_1,, pupae	3	0	0,903	0,153	0,0882

Hypothesized population mean 1,000

t = -1,095 with 2 degrees of freedom.

95 percent two-tailed confidence interval for the population mean: 0,524 to 1,283

Two-tailed P-value = 0,388

The difference between the mean of the sampled population and the hypothesized population mean is not great enough to reject the hypothesis that the difference is only due to random sample variability. There is not a significant difference between the two means (P = 0,388).

One-tailed P-value = 0,194

The hypothesized mean does not exceed the sample mean of the group by an amount great enough to exclude the possibility that the difference is due to random sampling variability. The hypothesis that the true mean is greater than or equal to the hypothesized mean cannot be rejected. (P = 0,194).

Figure 1D

One-Sample t-test

Normality Test (Shapiro-Wilk): Passed (P = 0,702)

Group Name	N	Missing	Mean	Std Dev	SEM
7_lrc_1, day 3	3	0	1,214	0,382	0,221

Hypothesized population mean 1,000

t = 0,968 with 2 degrees of freedom.

95 percent two-tailed confidence interval for the population mean: 0,264 to 2,164

Two-tailed P-value = 0,435

The difference between the mean of the sampled population and the hypothesized population mean is not great enough to reject the hypothesis that the difference is only due to random sample variability. There is not a significant difference between the two means (P = 0,435).

One-tailed P-value = 0,218

The sample mean of the group does not exceed the hypothesized mean by an amount great enough to exclude the possibility that the difference is due to random sampling variability. The hypothesis that the hypothesized mean is greater than or equal to the true mean cannot be rejected. (P = 0,218).

One-Sample t-test

Normality Test (Kolmogorov-Smirnov): Passed (P = 0,481)

Group Name	N	Missing	Mean	Std Dev	SEM
20_lrc_2, day 5	2	0	0,791	0,181	0,128

Hypothesized population mean 1,000

t = -1,629 with 1 degrees of freedom.

95 percent two-tailed confidence interval for the population mean: -0,837 to 2,420

Two-tailed P-value = 0,351

The difference between the mean of the sampled population and the hypothesized population mean is not great enough to reject the hypothesis that the difference is only due to random sample variability. There is not a significant difference between the two means (P = 0,351).

One-tailed P-value = 0,175

The hypothesized mean does not exceed the sample mean of the group by an amount great enough to exclude the possibility that the difference is due to random sampling variability. The hypothesis that the true mean is greater than or equal to the hypothesized mean cannot be rejected. (P = 0,175).

One-Sample t-test

Normality Test (Shapiro-Wilk): Passed (P = 0,234)

Group Name	N	Missing	Mean	Std Dev	SEM
31_lrc_1, pupae	3	0	1,143	0,136	0,0783

Hypothesized population mean 1,000

t = 1,832 with 2 degrees of freedom.

95 percent two-tailed confidence interval for the population mean: 0,806 to 1,480

Two-tailed P-value = 0,208

The difference between the mean of the sampled population and the hypothesized population mean is not great enough to reject the hypothesis that the difference is only due to random sample variability. There is not a significant difference between the two means (P = 0,208).

One-tailed P-value = 0,104

The sample mean of the group does not exceed the hypothesized mean by an amount great enough to exclude the possibility that the difference is due to random sampling variability. The hypothesis that the hypothesized mean is greater than or equal to the true mean cannot be rejected. (P = 0,104).

Figure 1E

One-Sample t-test

Normality Test (Kolmogorov-Smirnov): Passed (P = 0,481)

Group Name	N	Missing	Mean	Std Dev	SEM
7_lrc_1, day 3	2	0	1,379	0,0755	0,0534

Hypothesized population mean 1,000

t = 7,098 with 1 degrees of freedom.

95 percent two-tailed confidence interval for the population mean: 0,701 to 2,057

Two-tailed P-value = 0,0891

The difference between the mean of the sampled population and the hypothesized population mean is not great enough to reject the hypothesis that the difference is only due to random sample variability. There is not a significant difference between the two means (P = 0,089).

One-tailed P-value = 0,0446

The sample mean of the group exceeds the hypothesized mean by an amount that is greater than would be expected by chance, rejecting the hypothesis that the hypothesized mean is greater than or equal to the true mean. (P = 0,045).

One-Sample t-test

Normality Test (Shapiro-Wilk): Passed (P = 0,627)

Group Name	N	Missing	Mean	Std Dev	SEM
19_lrc_1, day 5	3	0	1,366	0,363	0,210

Hypothesized population mean 1,000

t = 1,746 with 2 degrees of freedom.

95 percent two-tailed confidence interval for the population mean: 0,464 to 2,267

Two-tailed P-value = 0,223

The difference between the mean of the sampled population and the hypothesized population mean is not great enough to reject the hypothesis that the difference is only due to random sample variability. There is not a significant difference between the two means (P = 0,223).

One-tailed P-value = 0,111

The sample mean of the group does not exceed the hypothesized mean by an amount great enough to exclude the possibility that the difference is due to random sampling variability. The hypothesis that the hypothesized mean is greater than or equal to the true mean cannot be rejected. (P = 0,111).

One-Sample t-test

Normality Test (Shapiro-Wilk): Passed (P = 0,186)

Group Name	N	Missing	Mean	Std Dev	SEM
31_lrc_1, pupae	3	0	0,844	0,141	0,0814

Hypothesized population mean 1,000

t = -1,911 with 2 degrees of freedom.

95 percent two-tailed confidence interval for the population mean: 0,494 to 1,195

Two-tailed P-value = 0,196

The difference between the mean of the sampled population and the hypothesized population mean is not great enough to reject the hypothesis that the difference is only due to random sample variability. There is not a significant difference between the two means (P = 0,196).

One-tailed P-value = 0,0981

The hypothesized mean does not exceed the sample mean of the group by an amount great enough to exclude the possibility that the difference is due to random sampling variability. The hypothesis that the true mean is greater than or equal to the hypothesized mean cannot be rejected. (P = 0,098).

Figure 1F

One-Sample t-test

Normality Test (Shapiro-Wilk): Passed (P = 0,335)

Group Name	N	Missing	Mean	Std Dev	SEM
10_fez2_1, day 3	3	0	1,226	0,126	0,0727

Hypothesized population mean 1,000

t = 3,105 with 2 degrees of freedom.

95 percent two-tailed confidence interval for the population mean: 0,913 to 1,538

Two-tailed P-value = 0,0900

The difference between the mean of the sampled population and the hypothesized population mean is not great enough to reject the hypothesis that the difference is only due to random sample variability. There is not a significant difference between the two means (P = 0,090).

One-tailed P-value = 0,0450

The sample mean of the group exceeds the hypothesized mean by an amount that is greater than would be expected by chance, rejecting the hypothesis that the hypothesized mean is greater than or equal to the true mean. (P = 0,045).

One-Sample t-test

Normality Test (Shapiro-Wilk): Passed (P = 0,760)

Group Name	N	Missing	Mean	Std Dev	SEM
22_fez2_1, day 5	3	0	0,753	0,0745	0,0430

Hypothesized population mean 1,000

t = -5,743 with 2 degrees of freedom.

95 percent two-tailed confidence interval for the population mean: 0,568 to 0,938

Two-tailed P-value = 0,0290

There is a statistically significant difference between the mean of the sampled population and the hypothesized population mean (P = 0,029).

One-tailed P-value = 0,0145

The hypothesized mean exceeds the sample mean of the group by an amount that is greater than would be expected by chance, rejecting the hypothesis that the true mean of group is greater than or equal to the hypothesized mean. (P = 0,015).

One-Sample t-test

Normality Test (Shapiro-Wilk): Passed (P = 0,604)

Group Name	N	Missing	Mean	Std Dev	SEM
34_fez2_1, pupae	3	0	1,026	0,0151	0,00873

Hypothesized population mean 1,000

t = 3,023 with 2 degrees of freedom.

95 percent two-tailed confidence interval for the population mean: 0,989 to 1,064

Two-tailed P-value = 0,0942

The difference between the mean of the sampled population and the hypothesized population mean is not great enough to reject the hypothesis that the difference is only due to random sample variability. There is not a significant difference between the two means (P = 0,094).

One-tailed P-value = 0,0471

The sample mean of the group exceeds the hypothesized mean by an amount that is greater than would be expected by chance, rejecting the hypothesis that the hypothesized mean is greater than or equal to the true mean. (P = 0,047).

Figure 1G

One-Sample t-test

Normality Test (Shapiro-Wilk): Passed (P = 0,555)

Group Name	N	Missing	Mean	Std Dev	SEM
10_fez2_1, day 3	3	0	0,774	0,141	0,0817

Hypothesized population mean 1,000

t = -2,769 with 2 degrees of freedom.

95 percent two-tailed confidence interval for the population mean: 0,423 to 1,125

Two-tailed P-value = 0,109

The difference between the mean of the sampled population and the hypothesized population mean is not great enough to reject the hypothesis that the difference is only due to random sample variability. There is not a significant difference between the two means (P = 0,109).

One-tailed P-value = 0,0547

The hypothesized mean does not exceed the sample mean of the group by an amount great enough to exclude the possibility that the difference is due to random sampling variability. The hypothesis that the true mean is greater than or equal to the hypothesized mean cannot be rejected. (P = 0,055).

One-Sample t-test

Normality Test (Shapiro-Wilk): Passed (P = 0,553)

Group Name	N	Missing	Mean	Std Dev	SEM
22_fez2_1, day 5	3	0	1,251	0,199	0,115

Hypothesized population mean 1,000

t = 2,183 with 2 degrees of freedom.

95 percent two-tailed confidence interval for the population mean: 0,757 to 1,744

Two-tailed P-value = 0,161

The difference between the mean of the sampled population and the hypothesized population mean is not great enough to reject the hypothesis that the difference is only due to random sample variability. There is not a significant difference between the two means (P = 0,161).

One-tailed P-value = 0,0804

The sample mean of the group does not exceed the hypothesized mean by an amount great enough to exclude the possibility that the difference is due to random sampling variability. The hypothesis that the hypothesized mean is greater than or equal to the true mean cannot be rejected. (P = 0,080).

One-Sample t-test

Normality Test (Shapiro-Wilk): Passed (P = 0,556)

Group Name	N	Missing	Mean	Std Dev	SEM
34_fez2_1, pupae	3	0	1,281	0,0324	0,0187

Hypothesized population mean 1,000

t = 14,998 with 2 degrees of freedom.

95 percent two-tailed confidence interval for the population mean: 1,200 to 1,361

Two-tailed P-value = 0,00442

There is a statistically significant difference between the mean of the sampled population and the hypothesized population mean (P = 0,004).

One-tailed P-value = 0,00221

The sample mean of the group exceeds the hypothesized mean by an amount that is greater than would be expected by chance, rejecting the hypothesis that the hypothesized mean is greater than or equal to the true mean. (P = 0,002).

Figure 2a

t-test

Normality Test (Shapiro-Wilk): Failed (P < 0,050)

Equal Variance Test (Brown-Forsythe): Passed (P = 0,697)

The result of the equal variance test indicates the likelihood that the two groups are sampled from populations with equal variances, but does not guarantee the equality or inequality of the two variances.

Group Name	N	Missing	Mean	Std Dev	SEM
EGFP IVT	24	0	0,908	0,167	0,0340
β-tub 2851 IVT	24	0	0,926	0,153	0,0312

Difference of means -0,0181

Use the results of Welch's test, where equal variances are not assumed, if the equality of the population variances of the two groups is in doubt.

Equal Variances Assumed (Student's t-test):

t = -0,391 with 46 degrees of freedom.

95 percent two-tailed confidence interval for difference of means: -0,111 to 0,0749

Two-tailed P-value = 0,697

The difference in the mean values of the two groups is not great enough to reject the possibility that the difference is due to random sampling variability. There is not a statistically significant difference between the input groups (P = 0,697).

One-tailed P-value = 0,349

The sample mean of group β-tub 2851 IVT does not exceed the sample mean of the group EGFP IVT by an amount great enough to exclude the possibility that the difference is due to random sampling variability. The hypothesis that the population mean of group EGFP IVT is greater than or equal to the population mean of group β-tub 2851 IVT cannot be rejected. (P = 0,349).

Equal Variances Not Assumed (Welch's t-test):

t = -0,391 with 45,666 degrees of freedom.

95 percent two-tailed confidence interval for difference of means: -0,111 to 0,0749

Two-tailed P-value = 0,697

The difference in the mean values of the two groups is not great enough to reject the possibility that the difference is due to random sampling variability. There is not a statistically significant difference between the input groups (P = 0,697).

One-tailed P-value = 0,349

The sample mean of group β-tub 2851 IVT does not exceed the sample mean of the group EGFP IVT by an amount great enough to exclude the possibility that the difference is due to random sampling variability. The hypothesis that the population mean of group EGFP IVT is greater than or equal to the population mean of group β-tub 2851 IVT cannot be rejected. (P = 0,349).

Box-Cox Transformation

Two-Sample T-Test and CI: C3, C2

Method

μ_1: population mean of C3 when C2 = EGFP IVT

μ_2: population mean of C3 when C2 = β-tub 2851 IVT

Difference: $\mu_1 - \mu_2$

Equal variances are not assumed for this analysis.

Descriptive Statistics: C3

C2	N	Mean	StDev	SE Mean
EGFP IVT	24	7698533333	3740213378	763467859
β-tub 2851 IVT	24	8213715622	3607999659	736479846

Estimation for Difference

Difference	95% CI for Difference
-515182288	(-2651732811, 1621368235)

Test

Null hypothesis	H_0: $\mu_1 - \mu_2 = 0$
Alternative hypothesis	H_1: $\mu_1 - \mu_2 \neq 0$

T-Value	DF	P-Value
-0.49	45	0.630

t-test

Normality Test (Shapiro-Wilk): Failed (P < 0,050)

Equal Variance Test (Brown-Forsythe): Passed (P = 0,098)

The result of the equal variance test indicates the likelihood that the two groups are sampled from populations with equal variances, but does not guarantee the equality or inequality of the two variances.

Group Name	N	Missing	Mean	Std Dev	SEM
βtub 2851 einfach	12	0	0,875	0,142	0,0411
EGFP einfach	12	0	0,903	0,0855	0,0247

Difference of means -0,0279

Use the results of Welch's test, where equal variances are not assumed, if the equality of the population variances of the two groups is in doubt.

Equal Variances Assumed (Student's t-test):

t = -0,583 with 22 degrees of freedom.

95 percent two-tailed confidence interval for difference of means: -0,127 to 0,0714

Two-tailed P-value = 0,566

The difference in the mean values of the two groups is not great enough to reject the possibility that the difference is due to random sampling variability. There is not a statistically significant difference between the input groups (P = 0,566).

One-tailed P-value = 0,283

The sample mean of group EGFP einfach does not exceed the sample mean of the group βtub 2851 einfach by an amount great enough to exclude the possibility that the difference is due to random sampling variability. The hypothesis that the population mean of group βtub 2851 einfach is greater than or equal to the population mean of group EGFP einfach cannot be rejected. (P = 0,283).

Equal Variances Not Assumed (Welch's t-test):

t = -0,583 with 18,028 degrees of freedom.

95 percent two-tailed confidence interval for difference of means: -0,129 to 0,0727

Two-tailed P-value = 0,567

The difference in the mean values of the two groups is not great enough to reject the possibility that the difference is due to random sampling variability. There is not a statistically significant difference between the input groups (P = 0,567).

One-tailed P-value = 0,284

The sample mean of group EGFP einfach does not exceed the sample mean of the group βtub 2851 einfach by an amount great enough to exclude the possibility that the difference is due to random sampling variability. The hypothesis that the population mean of group βtub 2851 einfach is greater than or equal to the population mean of group EGFP einfach cannot be rejected. (P = 0,284).

Box-Cox Transformation

Two-Sample T-Test and CI: C4, C1

Method

μ_1: population mean of C4 when C1 = EGFP einfach

μ_2: population mean of C4 when C1 = βtub 2851 einfach

Difference: $\mu_1 - \mu_2$

Equal variances are not assumed for this analysis.

Descriptive Statistics: C4

C1	N	Mean	StDev	SE Mean
EGFP einfach	12	6455650412	2557120655	738177149
βtub 2851 einfach	12	6260875000	3707638894	1070303157

Estimation for Difference

Difference	95% CI for Difference
194775412	(-2526521591, 2916072415)

Test

Null hypothesis	$H_0: \mu_1 - \mu_2 = 0$
Alternative hypothesis	$H_1: \mu_1 - \mu_2 \neq 0$

T-Value	DF	P-Value
0.15	19	0.882

t-test

Normality Test (Shapiro-Wilk): Failed (P < 0,050)

Equal Variance Test (Brown-Forsythe): Passed (P = 0,868)

The result of the equal variance test indicates the likelihood that the two groups are sampled from populations with equal variances, but does not guarantee the equality or inequality of the two variances.

Group Name	N	Missing	Mean	Std Dev	SEM
βtub 2851 6-fach	12	0	0,833	0,156	0,0449
EGFP 6-fach	12	0	0,892	0,124	0,0358

Difference of means -0,0591

Use the results of Welch's test, where equal variances are not assumed, if the equality of the population variances of the two groups is in doubt.

Equal Variances Assumed (Student's t-test):

t = -1,028 with 22 degrees of freedom.

95 percent two-tailed confidence interval for difference of means: -0,178 to 0,0601

Two-tailed P-value = 0,315

The difference in the mean values of the two groups is not great enough to reject the possibility that the difference is due to random sampling variability. There is not a statistically significant difference between the input groups (P = 0,315).

One-tailed P-value = 0,158

The sample mean of group EGFP 6-fach does not exceed the sample mean of the group βtub 2851 6-fach by an amount great enough to exclude the possibility that the difference is due to random sampling variability. The hypothesis that the population mean of group βtub 2851 6-fach is greater than or equal to the population mean of group EGFP 6-fach cannot be rejected. (P = 0,158).

Equal Variances Not Assumed (Welch's t-test):

t = -1,028 with 20,957 degrees of freedom.

95 percent two-tailed confidence interval for difference of means: -0,179 to 0,0605

Two-tailed P-value = 0,316

The difference in the mean values of the two groups is not great enough to reject the possibility that the difference is due to random sampling variability. There is not a statistically significant difference between the input groups (P = 0,316).

One-tailed P-value = 0,158

The sample mean of group EGFP 6-fach does not exceed the sample mean of the group βtub 2851 6-fach by an amount great enough to exclude the possibility that the difference is due to random sampling variability. The hypothesis that the population mean of group βtub 2851 6-fach is greater than or equal to the population mean of group EGFP 6-fach cannot be rejected. (P = 0,158).

Box-Cox Transformation

Two-Sample T-Test and CI: C4, C1

Method

μ_1: population mean of C4 when C1 = EGFP 6-fach

μ_2: population mean of C4 when C1 = βtub 2851 6-fach

Difference: $\mu_1 - \mu_2$

Equal variances are not assumed for this analysis.

Descriptive Statistics: C4

C1	N	Mean	StDev	SE Mean
EGFP 6-fach	12	69763445	31486318	9089317
βtub 2851 6-fach	12	56486667	30151641	8704029

Estimation for Difference

Difference	95% CI for Difference
13276779	(-12894631, 39448189)

Test

Null hypothesis H_0: $\mu_1 - \mu_2 = 0$

Alternative hypothesis H_1: $\mu_1 - \mu_2 \neq 0$

T-Value	DF	P-Value
1.05	21	0.303

t-test

Normality Test (Shapiro-Wilk): Failed (P < 0,050)

Equal Variance Test (Brown-Forsythe): Passed (P = 0,228)

The result of the equal variance test indicates the likelihood that the two groups are sampled from populations with equal variances, but does not guarantee the equality or inequality of the two variances.

Group Name	N	Missing	Mean	Std Dev	SEM
β-tub 4939 dreifach	12	0	0,867	0,144	0,0414
EGFP dreifach	12	0	0,908	0,0793	0,0229

Difference of means -0,0417

Use the results of Welch's test, where equal variances are not assumed, if the equality of the population variances of the two groups is in doubt.

Equal Variances Assumed (Student's t-test):

t = -0,880 with 22 degrees of freedom.

95 percent two-tailed confidence interval for difference of means: -0,140 to 0,0565

Two-tailed P-value = 0,388

The difference in the mean values of the two groups is not great enough to reject the possibility that the difference is due to random sampling variability. There is not a statistically significant difference between the input groups (P = 0,388).

One-tailed P-value = 0,194

The sample mean of group EGFP dreifach does not exceed the sample mean of the group β-tub 4939 dreifach by an amount great enough to exclude the possibility that the difference is due to random sampling variability. The hypothesis that the population mean of group β-tub 4939 dreifach is greater than or equal to the population mean of group EGFP dreifach cannot be rejected. (P = 0,194).

Equal Variances Not Assumed (Welch's t-test):

t = -0,880 with 17,141 degrees of freedom.

95 percent two-tailed confidence interval for difference of means: -0,141 to 0,0582

Two-tailed P-value = 0,391

The difference in the mean values of the two groups is not great enough to reject the possibility that the difference is due to random sampling variability. There is not a statistically significant difference between the input groups (P = 0,391).

One-tailed P-value = 0,195

The sample mean of group EGFP dreifach does not exceed the sample mean of the group β-tub 4939 dreifach by an amount great enough to exclude the possibility that the difference is due to random sampling variability. The hypothesis that the population mean of group β-tub 4939 dreifach is greater than or equal to the population mean of group EGFP dreifach cannot be rejected. (P = 0,195).

Box-Cox Transformation

Two-Sample T-Test and CI: C4, C1

Method

μ_1: population mean of C4 when C1 = EGFP dreifach

μ_2: population mean of C4 when C1 = β-tub 4939 dreifach

Difference: $\mu_1 - \mu_2$

Equal variances are not assumed for this analysis.

Descriptive Statistics: C4

C1	N	Mean	StDev	SE Mean
EGFP dreifach	12	70910833	23772348	6862486
β-tub 4939 dreifach	12	64190000	32285349	9319978

Estimation for Difference

Difference	95% CI for Difference
6720833	(-17421947, 30863614)

Test

Null hypothesis	H_0: $\mu_1 - \mu_2 = 0$
Alternative hypothesis	H_1: $\mu_1 - \mu_2 \neq 0$

T-Value	DF	P-Value
0.58	20	0.568

Box-Cox Transformation

Two-Sample T-Test and CI: C4, C1

Method

μ_1: population mean of C4 when C1 = eGFP 6-fach

μ_2: population mean of C4 when C1 = βtub 4939 6-fach

Difference: $\mu_1 - \mu_2$

Equal variances are not assumed for this analysis.

Descriptive Statistics: C4

C1	N	Mean	StDev	SE Mean
eGFP 6-fach	24	6658688051	3411898544	696450874
βtub 4939 6-fach	24	6329615763	3353619834	684554782

Estimation for Difference

Difference	95% CI for Difference
329072288	(-1637809829, 2295954405)

Test

Null hypothesis	H_0: $\mu_1 - \mu_2 = 0$
Alternative hypothesis	H_1: $\mu_1 - \mu_2 \neq 0$

T-Value	DF	P-Value
0.34	45	0.738

t-test

Normality Test (Shapiro-Wilk): Failed (P < 0,050)

Equal Variance Test (Brown-Forsythe): Passed (P = 0,768)

The result of the equal variance test indicates the likelihood that the two groups are sampled from populations with equal variances, but does not guarantee the equality or inequality of the two variances.

Group Name	N	Missing	Mean	Std Dev	SEM
βtub 2851 + βtub 4939 einfach	12	0	0,708	0,315	0,0908
EGFP einfach doppelt	12	0	0,771	0,303	0,0874

Difference of means -0,0629

Use the results of Welch's test, where equal variances are not assumed, if the equality of the population variances of the two groups is in doubt.

Equal Variances Assumed (Student's t-test):

t = -0,499 with 22 degrees of freedom.

95 percent two-tailed confidence interval for difference of means: -0,324 to 0,199

Two-tailed P-value = 0,623

The difference in the mean values of the two groups is not great enough to reject the possibility that the difference is due to random sampling variability. There is not a statistically significant difference between the input groups (P = 0,623).

One-tailed P-value = 0,311

The sample mean of group EGFP einfach doppelt does not exceed the sample mean of the group βtub 2851 + βtub 4939 einfach by an amount great enough to exclude the possibility that the difference is due to random sampling variability. The hypothesis that the population mean of group βtub 2851 + βtub 4939 einfach is greater than or equal to the population mean of group EGFP einfach doppelt cannot be rejected. (P = 0,311).

Equal Variances Not Assumed (Welch's t-test):

t = -0,499 with 21,968 degrees of freedom.

95 percent two-tailed confidence interval for difference of means: -0,324 to 0,199

Two-tailed P-value = 0,623

The difference in the mean values of the two groups is not great enough to reject the possibility that the difference is due to random sampling variability. There is not a statistically significant difference between the input groups (P = 0,623).

One-tailed P-value = 0,311

The sample mean of group EGFP einfach doppelt does not exceed the sample mean of the group βtub 2851 + βtub 4939 einfach by an amount great enough to exclude the possibility that the difference is due to random sampling variability. The hypothesis that the population mean of group βtub 2851 + βtub 4939 einfach is greater than or equal to the population mean of group EGFP einfach doppelt cannot be rejected. (P = 0,311).

Box-Cox Transformation

Two-Sample T-Test and CI: C4, C1

Method

μ_1: population mean of C4 when C1 = EGFP einfach doppelt

μ_2: population mean of C4 when C1 = βtub 2851 + βtub 4939 einfach

Difference: $\mu_1 - \mu_2$

Equal variances are not assumed for this analysis.

Descriptive Statistics: C4

C1	N	Mean	StDev	SE Mean
EGFP einfach doppelt	12	6789	3951	1141
βtub 2851 + βtub 4939 einfach	12	5925	3937	1136

Estimation for Difference

Difference	95% CI for Difference
864	(-2484, 4212)

Test

Null hypothesis	H_0: $\mu_1 - \mu_2 = 0$
Alternative hypothesis	H_1: $\mu_1 - \mu_2 \neq 0$

T-Value	DF	P-Value
0.54	21	0.597

Box-Cox Transformation

Two-Sample T-Test and CI: C4, C1

Method

μ_1: population mean of C4 when C1 = eGFP 6-fach

μ_2: population mean of C4 when C1 = βtub 2851, 4393 6-fach (dreifach, 2 targets)

Difference: $\mu_1 - \mu_2$

Equal variances are not assumed for this analysis.

Descriptive Statistics: C4

C1	N	Mean	StDev	SE Mean
eGFP 6-fach	12	423467	297863	85986
βtub 2851, 4393 6-fach (dreifach, 2 targets)	12	712110	247972	71583

Estimation for Difference

Difference	95% CI for Difference
-288643	(-521316, -55970)

Test

Null hypothesis	H_0: $\mu_1 - \mu_2 = 0$
Alternative hypothesis	H_1: $\mu_1 - \mu_2 \neq 0$

T-Value	DF	P-Value
-2.58	21	0.017

Figure 2B

Descriptive Statistics:

Column	Size	Missing	Mean	Std Dev	Std. Error	C.I. of Mean
Ache1	2	0	0,718	0,0261	0,0184	0,234
fez2	2	0	0,850	0,212	0,150	1,906
lrcE1	2	0	0,750	0,212	0,150	1,906
Sem1aE8	2	0	0,850	0,141	0,1000	1,271
Sem1aE15	2	0	0,875	0,106	0,0750	0,953
VATPase	2	0	0,925	0,106	0,0750	0,953
eGFP	2	0	0,833	0,101	0,0714	0,908
ßtub2851	2	0	0,950	0,0707	0,0500	0,635

Column	Range	Max	Min	Median	25%	75%
Ache1	0,0368	0,737	0,700	0,718	0,700	0,737
fez2	0,300	1,000	0,700	0,850	0,700	1,000
lrcE1	0,300	0,900	0,600	0,750	0,600	0,900
Sem1aE8	0,200	0,950	0,750	0,850	0,750	0,950
Sem1aE15	0,150	0,950	0,800	0,875	0,800	0,950
VATPase	0,150	1,000	0,850	0,925	0,850	1,000
eGFP	0,143	0,905	0,762	0,833	0,762	0,905
ßtub2851	0,1000	1,000	0,900	0,950	0,900	1,000

Column	Skewness	Kurtosis	K-S Dist.	K-S Prob.	SWilk W	SWilk Prob
Ache1	--	--	0,260	0,481	--	--
fez2	--	--	0,260	0,481	--	--
lrcE1	--	--	0,260	0,481	--	--
Sem1aE8	--	--	0,260	0,481	--	--
Sem1aE15	--	--	0,260	0,481	--	--
VATPase	--	--	0,260	0,481	--	--
eGFP	--	--	0,260	0,481	--	--
ßtub2851	--	--	0,260	0,481	--	--

Column	Sum	Sum of Squares
Ache1	1,437	1,033
fez2	1,700	1,490
lrcE1	1,500	1,170
Sem1aE8	1,700	1,465
Sem1aE15	1,750	1,543
VATPase	1,850	1,722
eGFP	1,667	1,399
ßtub2851	1,900	1,810

One Way Analysis of Variance

Normality Test (Shapiro-Wilk): Passed (P = 0,302)

Equal Variance Test (Brown-Forsythe): Failed (P < 0,050)

Test execution ended by user request, ANOVA on Ranks begun

Kruskal-Wallis One Way Analysis of Variance on Ranks

Group	N	Missing	Median	25%	75%
Ache1	2	0	0,718	0,700	0,737
fez2	2	0	0,850	0,700	1,000
lrcE1	2	0	0,750	0,600	0,900
Sem1aE8	2	0	0,850	0,750	0,950
Sem1aE15	2	0	0,875	0,800	0,950
VATPase	2	0	0,925	0,850	1,000
eGFP	2	0	0,833	0,762	0,905
ßtub2851	2	0	0,950	0,900	1,000

H = 5,605 with 7 degrees of freedom. (P = 0,586)

The differences in the median values among the treatment groups are not great enough to exclude the possibility that the difference is due to random sampling variability; there is not a statistically significant difference (P = 0,586)

One-way ANOVA: Ache1, fez2, lrc_E1, Sem1a_E8, Sem1a_E15, VATPase, eGFP, βtub2851

Method

Null hypothesis	All means are equal
Alternative hypothesis	Not all means are equal
Significance level	α = 0.05

Equal variances were not assumed for the analysis.

Factor Information

Factor	Levels	Values
Factor	8	Ache1, fez2, lrc_E1, Sem1a_E8, Sem1a_E15, VATPase, eGFP, βtub2851

Welch’s Test

Source	DF Num	DF Den	F-Value	P-Value
Factor	7	3.21282	1.83	0.324

Model Summary

R-sq	R-sq(adj)	R-sq(pred)
36.99%	0.00%	0.00%

Means

Factor	N	Mean	StDev	95% CI
Ache1	2	0.7184	0.0261	(0.4844, 0.9525)
fez2	2	0.850	0.212	(-1.056, 2.756)
lrc_E1	2	0.750	0.212	(-1.156, 2.656)
Sem1a_E8	2	0.850	0.141	(-0.421, 2.121)
Sem1a_E15	2	0.8750	0.1061	(-0.0780, 1.8280)
VATPase	2	0.9250	0.1061	(-0.0280, 1.8780)
eGFP	2	0.8333	0.1010	(-0.0743, 1.7409)
βtub2851	2	0.9500	0.0707	(0.3147, 1.5853)

Games-Howell Pairwise Comparisons

Grouping Information Using the Games-Howell Method and 95% Confidence

Factor	N	Mean	Grouping
βtub2851	2	0.9500	A
VATPase	2	0.9250	A
Sem1a_E15	2	0.8750	A
Sem1a_E8	2	0.850	A
fez2	2	0.850	A
eGFP	2	0.8333	A
lrc_E1	2	0.750	A
Ache1	2	0.7184	A

Means that do not share a letter are significantly different.

Games-Howell Simultaneous Tests for Differences of Means

Difference of Levels	Difference of Means	SE of Difference	95% CI	T-Value	Adjusted P-Value
fez2 - Ache1	0.132	0.151	(-4.367, 4.631)	0.87	0.950
lrc_E1 - Ache1	0.032	0.151	(-4.467, 4.531)	0.21	1.000
Sem1a_E8 - Ache1	0.132	0.102	(-2.639, 2.903)	1.29	0.846
Sem1a_E15 - Ache1	0.1566	0.0772	(-1.7231, 2.0362)	2.03	0.650
VATPase - Ache1	0.2066	0.0772	(-1.6731, 2.0862)	2.67	0.520
eGFP - Ache1	0.1149	0.0738	(-1.6371, 1.8670)	1.56	0.771
βtub2851 - Ache1	0.2316	0.0533	(-0.7665, 1.2297)	4.35	0.299
lrc_E1 - fez2	-0.100	0.212	(-2.054, 1.854)	-0.47	0.998
Sem1a_E8 - fez2	0.000	0.180	(-1.982, 1.982)	0.00	1.000
Sem1a_E15 - fez2	0.025	0.168	(-2.364, 2.414)	0.15	1.000
VATPase - fez2	0.075	0.168	(-2.314, 2.464)	0.45	0.998
eGFP - fez2	-0.017	0.166	(-2.495, 2.462)	-0.10	1.000
βtub2851 - fez2	0.100	0.158	(-3.099, 3.299)	0.63	0.988
Sem1a_E8 - lrc_E1	0.100	0.180	(-1.882, 2.082)	0.55	0.995
Sem1a_E15 - lrc_E1	0.125	0.168	(-2.264, 2.514)	0.75	0.978
VATPase - lrc_E1	0.175	0.168	(-2.214, 2.564)	1.04	0.919
eGFP - lrc_E1	0.083	0.166	(-2.395, 2.562)	0.50	0.997
βtub2851 - lrc_E1	0.200	0.158	(-2.999, 3.399)	1.26	0.855
Sem1a_E15 - Sem1a_E8	0.025	0.125	(-1.239, 1.289)	0.20	1.000
VATPase - Sem1a_E8	0.075	0.125	(-1.189, 1.339)	0.60	0.993
eGFP - Sem1a_E8	-0.017	0.123	(-1.300, 1.267)	-0.14	1.000
βtub2851 - Sem1a_E8	0.100	0.112	(-1.493, 1.693)	0.89	0.953
VATPase - Sem1a_E15	0.050	0.106	(-0.927, 1.027)	0.47	0.998
eGFP - Sem1a_E15	-0.042	0.104	(-0.999, 0.916)	-0.40	0.999
βtub2851 - Sem1a_E15	0.0750	0.0901	(-0.9161, 1.0661)	0.83	0.968
eGFP - VATPase	-0.092	0.104	(-1.049, 0.866)	-0.89	0.961
βtub2851 - VATPase	0.0250	0.0901	(-0.9661, 1.0161)	0.28	1.000
βtub2851 - eGFP	0.1167	0.0872	(-0.8075, 1.0408)	1.34	0.836

Figure 2C

Descriptive Statistics - data in lethality

Column	Size	Missing	Mean	Std Dev	Std. Error	C.I. of Mean
empty bact	2	0	0,671	0,136	0,0961	1,221
eGFP	2	0	0,704	0,0420	0,0297	0,378
Fez2	2	0	0,604	0,0994	0,0703	0,893
Sem1aE8	2	0	0,863	0,0523	0,0370	0,470
aCOP494	2	0	0,725	0,0820	0,0580	0,737
lrcE1	2	0	0,825	0,153	0,108	1,372

Column	Range	Max	Min	Median	25%	75%
empty bact	0,192	0,767	0,574	0,671	0,574	0,767
eGFP	0,0594	0,733	0,674	0,704	0,674	0,733
Fez2	0,141	0,674	0,533	0,604	0,533	0,674
Sem1aE8	0,0739	0,900	0,826	0,863	0,826	0,900
aCOP494	0,116	0,783	0,667	0,725	0,667	0,783
lrcE1	0,216	0,933	0,717	0,825	0,717	0,933

Column	Skewness	Kurtosis	K-S Dist.	K-S Prob.	SWilk W	SWilk Prob
empty bact	--	--	0,260	0,481	--	--
eGFP	--	--	0,260	0,481	--	--
Fez2	--	--	0,260	0,481	--	--
Sem1aE8	--	--	0,260	0,481	--	--
aCOP494	--	--	0,260	0,481	--	--
lrcE1	--	--	0,260	0,481	--	--

Column	Sum	Sum of Squares
empty bact	1,341	0,918
eGFP	1,407	0,992
Fez2	1,207	0,739
Sem1aE8	1,726	1,492
aCOP494	1,449	1,057
lrcE1	1,651	1,386

One Way Analysis of Variance

Normality Test (Shapiro-Wilk): Passed (P = 0,408)

Equal Variance Test (Brown-Forsythe): Failed (P < 0,050)

Test execution ended by user request, ANOVA on Ranks begun

Kruskal-Wallis One Way Analysis of Variance on Ranks

Group	N	Missing	Median	25%	75%
empty bact	2	0	0,671	0,574	0,767
eGFP	2	0	0,704	0,674	0,733
Fez2	2	0	0,604	0,533	0,674
Sem1aE8	2	0	0,863	0,826	0,900
aCOP494	2	0	0,725	0,667	0,783
lrcE1	2	0	0,825	0,717	0,933

H = 6,079 with 5 degrees of freedom. (P = 0,299)

The differences in the median values among the treatment groups are not great enough to exclude the possibility that the difference is due to random sampling variability; there is not a statistically significant difference (P = 0,299)

One-way ANOVA: Empty bact, eGFP, fez2, sem1a_E8, αCOP494_1B, lrc_E1

Method

Null hypothesis	All means are equal
Alternative hypothesis	Not all means are equal
Significance level	α = 0.05

Equal variances were not assumed for the analysis.

Factor Information

Factor	Levels	Values
Factor	6	Empty bact, eGFP, fez2, sem1a_E8, αCOP494_1B, lrc_E1

Welch's Test

Source	DF Num	DF Den	F-Value	P-Value
Factor	5	2.71285	1.76	0.355

Model Summary

R-sq	R-sq(adj)	R-sq(pred)
59.92%	26.52%	0.00%

Means

Factor	N	Mean	StDev	95% CI
Empty bact	2	0.6706	0.1359	(-0.5505, 1.8916)
eGFP	2	0.7036	0.0420	(0.3261, 1.0811)
fez2	2	0.6036	0.0994	(-0.2895, 1.4967)
sem1a_E8	2	0.8630	0.0523	(0.3935, 1.3326)
αCOP494_1B	2	0.7246	0.0820	(-0.0120, 1.4612)
lrc_E1	2	0.825	0.153	(-0.547, 2.197)

Games-Howell Pairwise Comparisons - Grouping Information Using the Games-Howell Method and 95% Confidence

Factor	N	Mean	Grouping
sem1a_E8	2	0.8630	A
lrc_E1	2	0.825	A
αCOP494_1B	2	0.7246	A
eGFP	2	0.7036	A
Empty bact	2	0.6706	A
fez2	2	0.6036	A

Means that do not share a letter are significantly different.

Games-Howell Simultaneous Tests for Differences of Means

Difference of Levels	Difference of Means	SE of Difference	95% CI	T-Value	Adjusted P-Value
eGFP - Empty bact	0.033	0.101	(-1.881, 1.947)	0.33	0.998
fez2 - Empty bact	-0.067	0.119	(-1.167, 1.033)	-0.56	0.984
sem1a_E8 - Empty bact	0.192	0.103	(-1.471, 1.856)	1.87	0.613
αCOP494_1B - Empty bact	0.054	0.112	(-1.151, 1.259)	0.48	0.991
lrc_E1 - Empty bact	0.155	0.145	(-1.064, 1.373)	1.07	0.863
fez2 - eGFP	-0.1000	0.0763	(-1.2364, 1.0364)	-1.31	0.780
sem1a_E8 - eGFP	0.1594	0.0474	(-0.2560, 0.5749)	3.36	0.265
αCOP494_1B - eGFP	0.0210	0.0651	(-0.7911, 0.8331)	0.32	0.998
lrc_E1 - eGFP	0.122	0.112	(-2.167, 2.411)	1.09	0.854
sem1a_E8 - fez2	0.2594	0.0794	(-0.7070, 1.2258)	3.27	0.323
αCOP494_1B - fez2	0.1210	0.0911	(-0.6676, 0.9096)	1.33	0.768
lrc_E1 - fez2	0.222	0.129	(-1.076, 1.519)	1.72	0.632
αCOP494_1B - sem1a_E8	-0.1384	0.0687	(-0.8423, 0.5655)	-2.01	0.544
lrc_E1 - sem1a_E8	-0.038	0.114	(-2.058, 1.983)	-0.33	0.998
lrc_E1 - αCOP494_1B	0.101	0.123	(-1.362, 1.563)	0.82	0.934

Figure 2D

One-way ANOVA: GusA, ßtub, Fez2, Lrc_E1, Sem1a_E8, Sem1a_E15, Ache1, VATPase, eGFP

Method

Null hypothesis	All means are equal
Alternative hypothesis	Not all means are equal
Significance level	α = 0.05

Equal variances were not assumed for the analysis.

Factor Information

Factor	Levels	Values
Factor	9	GusA, ßtub, Fez2, Lrc_E1, Sem1a_E8, Sem1a_E15, Ache1, VATPase, eGFP

Welch's Test

Source	DF Num	DF Den	F-Value	P-Value
Factor	8	7.30229	5.40	**0.017**

Model Summary

R-sq	R-sq(adj)	R-sq(pred)
65.74%	50.51%	22.90%

Means

Factor	N	Mean	StDev	95% CI
GusA	3	83.57	7.73	(64.38, 102.76)
ßtub	3	50.0	17.3	(7.0, 93.0)
Fez2	3	56.67	12.58	(25.41, 87.92)
Lrc_E1	3	71.67	2.89	(64.50, 78.84)
Sem1a_E8	3	75.56	7.70	(56.43, 94.68)
Sem1a_E15	3	51.67	12.58	(20.41, 82.92)
Ache1	3	58.33	2.89	(51.16, 65.50)
VATPase	3	50.00	8.66	(28.49, 71.51)
eGFP	3	65.00	10.00	(40.16, 89.84)

Games-Howell Pairwise Comparisons

Grouping Information Using the Games-Howell Method and 95% Confidence

Factor	N	Mean	Grouping
GusA	3	83.57	A
Sem1a_E8	3	75.56	A

Lrc_E1	3	71.67	A
eGFP	3	65.00	A
Ache1	3	58.33	A
Fez2	3	56.67	A
Sem1a_E15	3	51.67	A
VATPase	3	50.00	A
ßtub	3	50.0	A

Means that do not share a letter are significantly different.

Games-Howell Simultaneous Tests for Differences of Means

Difference of Levels	Difference of Means	SE of Difference	95% CI	T-Value	Adjusted P-Value
ßtub - GusA	-33.6	10.9	(-109.4, 42.2)	-3.07	0.321
Fez2 - GusA	-26.90	8.53	(-78.32, 24.51)	-3.16	0.274
Lrc_E1 - GusA	-11.90	4.76	(-47.33, 23.52)	-2.50	0.466
Sem1a_E8 - GusA	-8.02	6.30	(-41.86, 25.82)	-1.27	0.899
Sem1a_E15 - GusA	-31.90	8.53	(-83.32, 19.51)	-3.74	0.184
Ache1 - GusA	-25.24	4.76	(-60.66, 10.18)	-5.30	0.110
VATPase - GusA	-33.57	6.70	(-69.86, 2.72)	-5.01	0.065
eGFP - GusA	-18.57	7.30	(-59.22, 22.08)	-2.55	0.407
Fez2 - ßtub	6.7	12.4	(-63.4, 76.8)	0.54	0.999
Lrc_E1 - ßtub	21.7	10.1	(-69.5, 112.9)	2.14	0.592
Sem1a_E8 - ßtub	25.6	10.9	(-50.4, 101.5)	2.34	0.506
Sem1a_E15 - ßtub	1.7	12.4	(-68.4, 71.8)	0.13	1.000
Ache1 - ßtub	8.3	10.1	(-82.9, 99.5)	0.82	0.981
VATPase - ßtub	0.0	11.2	(-73.7, 73.7)	0.00	1.000
eGFP - ßtub	15.0	11.5	(-56.4, 86.4)	1.30	0.885
Lrc_E1 - Fez2	15.00	7.45	(-48.77, 78.77)	2.01	0.629
Sem1a_E8 - Fez2	18.89	8.52	(-32.54, 70.32)	2.22	0.530
Sem1a_E15 - Fez2	-5.0	10.3	(-60.2, 50.2)	-0.49	1.000
Ache1 - Fez2	1.67	7.45	(-62.11, 65.44)	0.22	1.000
VATPase - Fez2	-6.67	8.82	(-57.62, 44.28)	-0.76	0.992
eGFP - Fez2	8.33	9.28	(-42.98, 59.65)	0.90	0.980
Sem1a_E8 - Lrc_E1	3.89	4.75	(-31.35, 39.13)	0.82	0.983
Sem1a_E15 - Lrc_E1	-20.00	7.45	(-83.77, 43.77)	-2.68	0.438

**Ache1 - Lrc_E1**	**-13.33**	**2.36**	**(-26.00, -0.67)**	**-5.66**	**0.042**
VATPase - Lrc_E1	-21.67	5.27	(-62.47, 19.14)	-4.11	0.198
eGFP - Lrc_E1	-6.67	6.01	(-55.32, 41.99)	-1.11	0.931
Sem1a_E15 - Sem1a_E8	-23.89	8.52	(-75.32, 27.54)	-2.81	0.351
Ache1 - Sem1a_E8	-17.22	4.75	(-52.46, 18.02)	-3.63	0.244
VATPase - Sem1a_E8	-25.56	6.69	(-61.79, 10.68)	-3.82	0.149
eGFP - Sem1a_E8	-10.56	7.29	(-51.20, 30.09)	-1.45	0.836
Ache1 - Sem1a_E15	6.67	7.45	(-57.11, 70.44)	0.89	0.972
VATPase - Sem1a_E15	-1.67	8.82	(-52.62, 49.28)	-0.19	1.000
eGFP - Sem1a_E15	13.33	9.28	(-37.98, 64.65)	1.44	0.841
VATPase - Ache1	-8.33	5.27	(-49.14, 32.47)	-1.58	0.780
eGFP - Ache1	6.67	6.01	(-41.99, 55.32)	1.11	0.931
eGFP - VATPase	15.00	7.64	(-26.53, 56.53)	1.96	0.620

Figure 2E

One Way Analysis of Variance - data given in lethality

Normality Test (Shapiro-Wilk): Passed (P = 0,230)

Equal Variance Test (Brown-Forsythe): Passed (P = 0,899)

Group Name	N	Missing	Mean	Std Dev	SEM
Ache1	3	0	0,433	0,0764	0,0441
Fez2	3	0	0,387	0,191	0,110
lrcE1	3	0	0,517	0,176	0,101
sem1aE8	3	0	0,600	0,000	0,000
Sem1aE15	3	0	0,429	0,168	0,0967
VATPase	3	0	0,400	0,180	0,104
eGFP	3	0	0,367	0,104	0,0601
ßtub	3	0	0,367	0,189	0,109
GusA	3	0	0,400	0,150	0,0866

Source of Variation	DF	SS	MS	F	P
Between Groups	8	0,144	0,0180	0,798	0,612
Residual	18	0,406	0,0225		
Total	26	0,550			

The differences in the mean values among the treatment groups are not great enough to exclude the possibility that the difference is due to random sampling variability; there is not a statistically significant difference (P = 0,612).

Power of performed test with alpha = 0,050: 0,050

The power of the performed test (0,050) is below the desired power of 0,800.
Less than desired power indicates you are less likely to detect a difference when one actually exists. Negative results should be interpreted cautiously.

Figure 2F

One Way Analysis of Variance – data given in lethality

Normality Test (Shapiro-Wilk): Passed (P = 0,202)

Equal Variance Test (Brown-Forsythe): Failed (P < 0,050)

Test execution ended by user request, ANOVA on Ranks begun

Kruskal-Wallis One Way Analysis of Variance on Ranks

Group	N	Missing	Median	25%	75%
empty bact	2	0	0,859	0,717	1,000
eGFP	2	0	0,814	0,652	0,975
fez2	2	0	0,843	0,761	0,925
sem1E8	2	0	0,723	0,522	0,925
aCOP494	2	0	0,876	0,826	0,925
lrcE1	2	0	0,736	0,522	0,950

H = 0,998 with 5 degrees of freedom. (P = 0,963)

The differences in the median values among the treatment groups are not great enough to exclude the possibility that the difference is due to random sampling variability; there is not a statistically significant difference (P = 0,963)

One-way ANOVA: Empty bact, eGFP, fez2, sem1a_E8, αCOP494_1B, lrc_E1

Method

Null hypothesis	All means are equal
Alternative hypothesis	Not all means are equal
Significance level	α = 0.05

Equal variances were not assumed for the analysis.

Factor Information

Factor	Levels	Values
Factor	6	Empty bact, eGFP, fez2, sem1a_E8, αCOP494_1B, lrc_E1

Welch’s Test

Source	DF Num	DF Den	F-Value	P-Value
Factor	5	2.64824	0.10	0.985

Model Summary

R-sq	R-sq(adj)	R-sq(pred)
12.77%	0.00%	0.00%

Means

Factor	N	Mean	StDev	95% CI
Empty bact	2	0.859	0.200	(-0.937, 2.654)

eGFP	2	0.814	0.228	(-1.237, 2.865)
fez2	2	0.8429	0.1161	(-0.1998, 1.8857)
sem1a_E8	2	0.723	0.285	(-1.839, 3.285)
αCOP494_1B	2	0.8755	0.0699	(0.2471, 1.5039)
lrc_E1	2	0.736	0.303	(-1.985, 3.457)

Games-Howell Pairwise Comparisons

Grouping Information Using the Games-Howell Method and 95% Confidence

Factor	N	Mean	Grouping
αCOP494_1B	2	0.8755	A
Empty bact	2	0.859	A
fez2	2	0.8429	A
eGFP	2	0.814	A
lrc_E1	2	0.736	A
sem1a_E8	2	0.723	A

Means that do not share a letter are significantly different.

Games-Howell Simultaneous Tests for Differences of Means

Difference of Levels	Difference of Means	SE of Difference	95% CI	T-Value	Adjusted P-Value
eGFP - Empty bact	-0.045	0.215	(-1.862, 1.772)	-0.21	1.000
fez2 - Empty bact	-0.016	0.163	(-1.830, 1.798)	-0.10	1.000
sem1a_E8 - Empty bact	-0.135	0.246	(-2.479, 2.208)	-0.55	0.985
αCOP494_1B - Empty bact	0.017	0.150	(-2.590, 2.624)	0.11	1.000
lrc_E1 - Empty bact	-0.123	0.257	(-2.677, 2.431)	-0.48	0.992
fez2 - eGFP	0.029	0.181	(-2.245, 2.303)	0.16	1.000
sem1a_E8 - eGFP	-0.090	0.258	(-2.357, 2.176)	-0.35	0.998
αCOP494_1B - eGFP	0.062	0.169	(-3.169, 3.293)	0.37	0.997
lrc_E1 - eGFP	-0.078	0.268	(-2.510, 2.355)	-0.29	0.999
sem1a_E8 - fez2	-0.120	0.218	(-3.471, 3.232)	-0.55	0.983
αCOP494_1B - fez2	0.0326	0.0958	(-0.9972, 1.0624)	0.34	0.998
lrc_E1 - fez2	-0.107	0.229	(-3.822, 3.608)	-0.47	0.991
αCOP494_1B - sem1a_E8	0.152	0.208	(-4.356, 4.660)	0.73	0.950
lrc_E1 - sem1a_E8	0.013	0.294	(-2.437, 2.462)	0.04	1.000
lrc_E1 - αCOP494_1B	-0.140	0.220	(-5.046, 4.766)	-0.64	0.968

Figure 5A

One Way Analysis of Variance

Normality Test (Shapiro-Wilk): Passed (P = 0,903)

Equal Variance Test (Brown-Forsythe): Failed (P < 0,050)

Group Name	N	Missing	Mean	Std Dev	SEM
lrc	2	0	81,250	19,445	13,750
fez2	2	0	65,000	49,497	35,000
none scr	2	0	88,750	15,910	11,250

Source of Variation	DF	SS	MS	F	P
Between Groups	2	589,583	294,792	0,287	0,769
Residual	3	3081,250	1027,083		
Total	5	3670,833			

The differences in the mean values among the treatment groups are not great enough to exclude the possibility that the difference is due to random sampling variability; there is not a statistically significant difference (P = 0,769).

Power of performed test with alpha = 0,050: 0,050

The power of the performed test (0,050) is below the desired power of 0,800.
Less than desired power indicates you are less likely to detect a difference when one actually exists. Negative results should be interpreted cautiously.

Statistics

Variable	N	N*	Mean	SE Mean	StDev	Variance	CoefVar	Minimum	Q1	Median	Q3	Maximum
lrc	2	0	81.3	13.8	19.4	378.1	23.93	67.5	*	81.3	*	95.0
fez2	2	0	65.0	35.0	49.5	2450.0	76.15	30.0	*	65.0	*	100.0
none scr	2	0	88.8	11.3	15.9	253.1	17.93	77.5	*	88.8	*	100.0

One-way ANOVA: lrc, fez2, none scr

Method

Null hypothesis	All means are equal
Alternative hypothesis	Not all means are equal
Significance level	α = 0.05

Equal variances were not assumed for the analysis.

Factor Information

Factor	Levels	Values
Factor	3	lrc, fez2, none scr

Welch's Test

Source	DF Num	DF Den	F-Value	P-Value
Factor	2	1.82124	0.19	0.844

Model Summary

R-sq	R-sq(adj)	R-sq(pred)
16.06%	0.00%	0.00%

Means

Factor	N	Mean	StDev	95% CI
lrc	2	81.3	19.4	(-93.5, 256.0)
fez2	2	65.0	49.5	(-379.7, 509.7)
none scr	2	88.8	15.9	(-54.2, 231.7)

Games-Howell Pairwise Comparisons

Grouping Information Using the Games-Howell Method and 95% Confidence

Factor	N	Mean	Grouping
none scr	2	88.8	A
lrc	2	81.3	A
fez2	2	65.0	A

Means that do not share a letter are significantly different.

Figure 5B

t-test

Normality Test (Shapiro-Wilk): Passed (P = 0,191)

Equal Variance Test (Brown-Forsythe): Passed (P = 0,860)

The result of the equal variance test indicates the likelihood that the two groups are sampled from populations with equal variances, but does not guarantee the equality or inequality of the two variances.

Group Name	N	Missing	Mean	Std Dev	SEM
fez2 v1	2	0	75,000	17,678	12,500
none scr	3	0	71,667	14,216	8,207

Difference of means 3,333

Use the results of Welch's test, where equal variances are not assumed, if the equality of the population variances of the two groups is in doubt.

Equal Variances Assumed (Student's t-test):

t = 0,236 with 3 degrees of freedom.

95 percent two-tailed confidence interval for difference of means: -41,569 to 48,235

Two-tailed P-value = 0,828

The difference in the mean values of the two groups is not great enough to reject the possibility that the difference is due to random sampling variability. There is not a statistically significant difference between the input groups (P = 0,828).

One-tailed P-value = 0,414

The sample mean of group fez2 v1 does not exceed the sample mean of the group none scr by an amount great enough to exclude the possibility that the difference is due to random sampling variability. The hypothesis that the population mean of group none scr is greater than or equal to the population mean of group fez2 v1 cannot be rejected. (P = 0,414).

Equal Variances Not Assumed (Welch's t-test):

t = 0,223 with 1,874 degrees of freedom.

95 percent two-tailed confidence interval for difference of means: -186,671 to 193,337

Two-tailed P-value = 0,846

The difference in the mean values of the two groups is not great enough to reject the possibility that the difference is due to random sampling variability. There is not a statistically significant difference between the input groups (P = 0,846).

One-tailed P-value = 0,423

The sample mean of group fez2 v1 does not exceed the sample mean of the group none scr by an amount great enough to exclude the possibility that the difference is due to random sampling variability. The hypothesis that the population mean of group none scr is greater than or equal to the population mean of group fez2 v1 cannot be rejected. (P = 0,423).

t-test

Normality Test (Shapiro-Wilk): Passed (P = 0,683)

Equal Variance Test (Brown-Forsythe): Failed (P < 0,050)

The result of the equal variance test indicates the likelihood that the two groups are sampled from populations with equal variances, but does not guarantee the equality or inequality of the two variances.

Group Name	N	Missing	Mean	Std Dev	SEM
fez2v2	2	0	81,250	1,768	1,250
non scr	2	0	97,500	0,000	0,000

Difference of means -16,250

Use the results of Welch's test, where equal variances are not assumed, if the equality of the population variances of the two groups is in doubt.

Equal Variances Assumed (Student's t-test):

t = -13,000 with 2 degrees of freedom.

95 percent two-tailed confidence interval for difference of means: -21,628 to -10,872

Two-tailed P-value = 0,00587

The difference in the mean values of the two groups is greater than would be expected by chance; there is a statistically significant difference between the input groups (P = 0,006).

One-tailed P-value = 0,00293

The sample mean of group non scr exceeds the sample mean of group fez2v2 by an amount that is greater than would be expected by chance, rejecting the hypothesis that the population mean of group fez2v2 is greater than or equal to the population mean of group non scr. (P = 0,003).

Equal Variances Not Assumed (Welch's t-test):

t = -13,000 with 1,000 degrees of freedom.

95 percent two-tailed confidence interval for difference of means: -32,133 to -0,367

Two-tailed P-value = 0,0489

The difference in the mean values of the two groups is greater than would be expected by chance; there is a statistically significant difference between the input groups (P = 0,049).

One-tailed P-value = 0,0244

The sample mean of group non scr exceeds the sample mean of group fez2v2 by an amount that is greater than would be expected by chance, rejecting the hypothesis that the population mean of group fez2v2 is greater than or equal to the population mean of group non scr. (P = 0,024).

t-test

Normality Test (Shapiro-Wilk): Passed (P = 0,683)

Equal Variance Test (Brown-Forsythe): Failed (P < 0,050)

The result of the equal variance test indicates the likelihood that the two groups are sampled from populations with equal variances, but does not guarantee the equality or inequality of the two variances.

Group Name	N	Missing	Mean	Std Dev	SEM
lrc	2	0	75,000	10,607	7,500
non scr	2	0	97,500	0,000	0,000

Difference of means -22,500

Use the results of Welch's test, where equal variances are not assumed, if the equality of the population variances of the two groups is in doubt.

Equal Variances Assumed (Student's t-test):

t = -3,000 with 2 degrees of freedom.

95 percent two-tailed confidence interval for difference of means: -54,770 to 9,770

Two-tailed P-value = 0,0955

The difference in the mean values of the two groups is not great enough to reject the possibility that the difference is due to random sampling variability. There is not a statistically significant difference between the input groups (P = 0,095).

One-tailed P-value = 0,0477

The sample mean of group non scr exceeds the sample mean of group lrc by an amount that is greater than would be expected by chance, rejecting the hypothesis that the population mean of group lrc is greater than or equal to the population mean of group non scr. (P = 0,048).

Equal Variances Not Assumed (Welch's t-test):

t = -3,000 with 1,000 degrees of freedom.

95 percent two-tailed confidence interval for difference of means: -117,797 to 72,797

Two-tailed P-value = 0,205

The difference in the mean values of the two groups is not great enough to reject the possibility that the difference is due to random sampling variability. There is not a statistically significant difference between the input groups (P = 0,205).

One-tailed P-value = 0,102

The sample mean of group non scr does not exceed the sample mean of the group lrc by an amount great enough to exclude the possibility that the difference is due to random sampling variability. The hypothesis that the population mean of group lrc is greater than or equal to the population mean of group non scr cannot be rejected. (P = 0,102).

t-test

Normality Test (Shapiro-Wilk): Passed (P = 0,980)

Equal Variance Test (Brown-Forsythe): Failed (P < 0,050)

The result of the equal variance test indicates the likelihood that the two groups are sampled from populations with equal variances, but does not guarantee the equality or inequality of the two variances.

Group Name	N	Missing	Mean	Std Dev	SEM
lrc	2	0	75,000	10,607	7,500
fez2v2	2	0	81,250	1,768	1,250

Difference of means -6,250

Use the results of Welch's test, where equal variances are not assumed, if the equality of the population variances of the two groups is in doubt.

Equal Variances Assumed (Student's t-test):

t = -0,822 with 2 degrees of freedom.

95 percent two-tailed confidence interval for difference of means: -38,965 to 26,465

Two-tailed P-value = 0,497

The difference in the mean values of the two groups is not great enough to reject the possibility that the difference is due to random sampling variability. There is not a statistically significant difference between the input groups (P = 0,497).

One-tailed P-value = 0,249

The sample mean of group fez2v2 does not exceed the sample mean of the group lrc by an amount great enough to exclude the possibility that the difference is due to random sampling variability. The hypothesis that the population mean of group lrc is greater than or equal to the population mean of group fez2v2 cannot be rejected. (P = 0,249).

Equal Variances Not Assumed (Welch's t-test):

t = -0,822 with 1,056 degrees of freedom.

95 percent two-tailed confidence interval for difference of means: -102,861 to 90,361

Two-tailed P-value = 0,556

The difference in the mean values of the two groups is not great enough to reject the possibility that the difference is due to random sampling variability. There is not a statistically significant difference between the input groups (P = 0,556).

One-tailed P-value = 0,278

The sample mean of group fez2v2 does not exceed the sample mean of the group lrc by an amount great enough to exclude the possibility that the difference is due to random sampling variability. The hypothesis that the population mean of group lrc is greater than or equal to the population mean of group fez2v2 cannot be rejected. (P = 0,278).

Figure 5C

t-test

Normality Test (Shapiro-Wilk): Passed (P = 0,325)

Equal Variance Test (Brown-Forsythe): Passed (P = 1,000)

The result of the equal variance test indicates the likelihood that the two groups are sampled from populations with equal variances, but does not guarantee the equality or inequality of the two variances.

**Group Name**	**N**	**Missing**	**Mean**	**Std Dev**	**SEM**
fez2 nuc	2	0	98,750	1,768	1,250
non scr	3	0	100,000	0,000	0,000

Difference of means -1,250

Use the results of Welch's test, where equal variances are not assumed, if the equality of the population variances of the two groups is in doubt.

Equal Variances Assumed (Student's t-test):

t = -1,342 with 3 degrees of freedom.

95 percent two-tailed confidence interval for difference of means: -4,215 to 1,715

Two-tailed P-value = 0,272

The difference in the mean values of the two groups is not great enough to reject the possibility that the difference is due to random sampling variability. There is not a statistically significant difference between the input groups (P = 0,272).

One-tailed P-value = 0,136

The sample mean of group non scr does not exceed the sample mean of the group fez2 nuc by an amount great enough to exclude the possibility that the difference is due to random sampling variability. The hypothesis that the population mean of group fez2 nuc is greater than or equal to the population mean of group non scr cannot be rejected. (P = 0,136).

Equal Variances Not Assumed (Welch's t-test):

t = -1,000 with 1,000 degrees of freedom.

95 percent two-tailed confidence interval for difference of means: -17,133 to 14,633

Two-tailed P-value = 0,500

The difference in the mean values of the two groups is not great enough to reject the possibility that the difference is due to random sampling variability. There is not a statistically significant difference between the input groups (P = 0,500).

One-tailed P-value = 0,250

The sample mean of group non scr does not exceed the sample mean of the group fez2 nuc by an amount great enough to exclude the possibility that the difference is due to random sampling variability. The hypothesis that the population mean of group fez2 nuc is greater than or equal to the population mean of group non scr cannot be rejected. (P = 0,250).

Figure 6

One Way Analysis of Variance

Normality Test (Shapiro-Wilk): Passed (P = 0,730)

Equal Variance Test (Brown-Forsythe): Passed (P = 0,102)

Group Name	N	Missing	Mean	Std Dev	SEM
Males dsx	5	0	45,740	6,594	2,949
Males scramble	3	0	46,567	10,706	6,181
Males none	2	0	55,000	7,071	5,000

Source of Variation	DF	SS	MS	F	P
Between Groups	2	129,445	64,723	1,000	0,415
Residual	7	453,159	64,737		
Total	9	582,604			

The differences in the mean values among the treatment groups are not great enough to exclude the possibility that the difference is due to random sampling variability; there is not a statistically significant difference (P = 0,415).

Power of performed test with alpha = 0,050: 0,050

The power of the performed test (0,050) is below the desired power of 0,800.
Less than desired power indicates you are less likely to detect a difference when one actually exists. Negative results should be interpreted cautiously.

Figure 7A

Descriptive Statistics – data in lethality

Column	Size	Missing	Mean	Std Dev	Std. Error	C.I. of Mean
H2O	2	0	0,0500	0,0236	0,0167	0,212
Gus 1500	2	0	0,0667	0,0943	0,0667	0,847
eGFP 1500	2	0	0,0318	0,000713	0,000504	0,00640
fez 500	2	0	0,167	0,0943	0,0667	0,847
fez 1000	2	0	0,0667	0,0471	0,0333	0,424
fez 1500	2	0	0,0333	0,000	0,000	0,000
lrc 500	2	0	0,117	0,0236	0,0167	0,212
lrc 1000	2	0	0,133	0,141	0,1000	1,271
lrc 1500	2	0	0,117	0,0236	0,0167	0,212
sem 1500	2	0	0,150	0,0707	0,0500	0,635

Column	Range	Max	Min	Median	25%	75%
H2O	0,0333	0,0667	0,0333	0,0500	0,0333	0,0667
Gus 1500	0,133	0,133	0,000	0,0667	0,000	0,133
eGFP 1500	0,00101	0,0323	0,0313	0,0318	0,0313	0,0323
fez 500	0,133	0,233	0,1000	0,167	0,1000	0,233
fez 1000	0,0667	0,1000	0,0333	0,0667	0,0333	0,1000
fez 1500	0,000	0,0333	0,0333	0,0333	0,0333	0,0333
lrc 500	0,0333	0,133	0,1000	0,117	0,1000	0,133
lrc 1000	0,200	0,233	0,0333	0,133	0,0333	0,233
lrc 1500	0,0333	0,133	0,1000	0,117	0,1000	0,133
sem 1500	0,1000	0,200	0,1000	0,150	0,1000	0,200

Column	Skewness	Kurtosis	K-S Dist.	K-S Prob.	SWilk W	SWilk Prob
H2O	--	--	0,260	0,481	--	--
Gus 1500	--	--	0,260	0,481	--	--
eGFP 1500	--	--	0,260	0,481	--	--
fez 500	--	--	0,260	0,481	--	--
fez 1000	--	--	0,260	0,481	--	--
fez 1500	--	--	0,000	<0,001	--	--
lrc 500	--	--	0,260	0,481	--	--
lrc 1000	--	--	0,260	0,481	--	--
lrc 1500	--	--	0,260	0,481	--	--
sem 1500	--	--	0,260	0,481	--	--

Column	Sum	Sum of Squares
H2O	0,1000	0,00556
Gus 1500	0,133	0,0178
eGFP 1500	0,0635	0,00202
fez 500	0,333	0,0644
fez 1000	0,133	0,0111
fez 1500	0,0667	0,00222
lrc 500	0,233	0,0278
lrc 1000	0,267	0,0556
lrc 1500	0,233	0,0278
sem 1500	0,300	0,0500

One Way Analysis of Variance

Normality Test (Shapiro-Wilk): Passed (P = 0,949)

Equal Variance Test (Brown-Forsythe): Failed (P < 0,050)

Test execution ended by user request, ANOVA on Ranks begun

Kruskal-Wallis One Way Analysis of Variance on Ranks

Group	N	Missing	Median	25%	75%
H2O	2	0	0,0500	0,0333	0,0667
Gus 1500	2	0	0,0667	0,000	0,133
eGFP 1500	2	0	0,0318	0,0313	0,0323
fez 500	2	0	0,167	0,1000	0,233
fez 1000	2	0	0,0667	0,0333	0,1000
fez 1500	2	0	0,0333	0,0333	0,0333
lrc 500	2	0	0,117	0,1000	0,133
lrc 1000	2	0	0,133	0,0333	0,233
lrc 1500	2	0	0,117	0,1000	0,133
sem 1500	2	0	0,150	0,1000	0,200

H = 10,461 with 9 degrees of freedom. (P = 0,314)

The differences in the median values among the treatment groups are not great enough to exclude the possibility that the difference is due to random sampling variability; there is not a statistically significant difference (P = 0,314)

One-way ANOVA: H2O, Gus 1500, eGFP 1500, fez 500, fez 1000, lrc 500, lrc 1000, lrc 1500, Sem 1500 – data in lethality

Method

Null hypothesis	All means are equal
Alternative hypothesis	Not all means are equal
Significance level	α = 0.05

Equal variances were not assumed for the analysis.

Factor Information

Factor	Levels	Values
Factor	9	H2O, Gus 1500, eGFP 1500, fez 500, fez 1000, lrc 500, lrc 1000, lrc 1500, Sem 1500

Welch's Test

Source	DF Num	DF Den	F-Value	P-Value
Factor	8	3.33600	3.39	0.154

Model Summary

R-sq	R-sq(adj)	R-sq(pred)
43.54%	0.00%	0.00%

Means

Factor	N	Mean	StDev	95% CI
H2O	2	0.0500	0.0236	(-0.1618, 0.2618)
Gus 1500	2	0.0667	0.0943	(-0.7804, 0.9137)
eGFP 1500	2	0.031754	0.000713	(0.025350, 0.038158)
fez 500	2	0.1667	0.0943	(-0.6804, 1.0137)
fez 1000	2	0.0667	0.0471	(-0.3569, 0.4902)
lrc 500	2	0.1167	0.0236	(-0.0951, 0.3284)
lrc 1000	2	0.133	0.141	(-1.137, 1.404)
lrc 1500	2	0.1167	0.0236	(-0.0951, 0.3284)
Sem 1500	2	0.1500	0.0707	(-0.4853, 0.7853)

Games-Howell Pairwise Comparisons

Grouping Information Using the Games-Howell Method and 95% Confidence

Factor	N	Mean	Grouping
fez 500	2	0.1667	A
Sem 1500	2	0.1500	A
lrc 1000	2	0.133	A
lrc 1500	2	0.1167	A
lrc 500	2	0.1167	A
fez 1000	2	0.0667	A
Gus 1500	2	0.0667	A
H2O	2	0.0500	A
eGFP 1500	2	0.031754	A

Means that do not share a letter are significantly different.

Games-Howell Simultaneous Tests for Differences of Means

Difference of Levels	Difference of Means	SE of Difference	95% CI	T-Value	Adjusted P-Value
Gus 1500 - H2O	0.0167	0.0687	(-1.7122, 1.7455)	0.24	1.000
eGFP 1500 - H2O	-0.0182	0.0167	(-0.5738, 0.5373)	-1.09	0.914
fez 500 - H2O	0.1167	0.0687	(-1.6122, 1.8455)	1.70	0.755
fez 1000 - H2O	0.0167	0.0373	(-0.5362, 0.5695)	0.45	0.999
lrc 500 - H2O	0.0667	0.0236	(-0.1590, 0.2923)	2.83	0.421
lrc 1000 - H2O	0.083	0.101	(-2.882, 3.048)	0.82	0.969
lrc 1500 - H2O	0.0667	0.0236	(-0.1590, 0.2923)	2.83	0.421
Sem 1500 - H2O	0.1000	0.0527	(-1.0113, 1.2113)	1.90	0.698
eGFP 1500 - Gus 1500	-0.0349	0.0667	(-2.2661, 2.1963)	-0.52	0.996
fez 500 - Gus 1500	0.1000	0.0943	(-0.8027, 1.0027)	1.06	0.938
fez 1000 - Gus 1500	0.0000	0.0745	(-1.1057, 1.1057)	0.00	1.000
lrc 500 - Gus 1500	0.0500	0.0687	(-1.6789, 1.7789)	0.73	0.982
lrc 1000 - Gus 1500	0.067	0.120	(-1.308, 1.442)	0.55	0.997
lrc 1500 - Gus 1500	0.0500	0.0687	(-1.6789, 1.7789)	0.73	0.982
Sem 1500 - Gus 1500	0.0833	0.0833	(-0.7929, 0.9596)	1.00	0.949

fez 500 - eGFP 1500	0.1349	0.0667	(-2.0963, 2.3661)	2.02	0.682
fez 1000 - eGFP 1500	0.0349	0.0333	(-1.0799, 1.1497)	1.05	0.924
lrc 500 - eGFP 1500	0.0849	0.0167	(-0.4707, 0.6405)	5.09	0.318
lrc 1000 - eGFP 1500	0.102	0.100	(-3.246, 3.449)	1.02	0.931
lrc 1500 - eGFP 1500	0.0849	0.0167	(-0.4707, 0.6405)	5.09	0.318
Sem 1500 - eGFP 1500	0.1182	0.0500	(-1.5549, 1.7913)	2.36	0.612
fez 1000 - fez 500	-0.1000	0.0745	(-1.2057, 1.0057)	-1.34	0.855
lrc 500 - fez 500	-0.0500	0.0687	(-1.7789, 1.6789)	-0.73	0.982
lrc 1000 - fez 500	-0.033	0.120	(-1.408, 1.342)	-0.28	1.000
lrc 1500 - fez 500	-0.0500	0.0687	(-1.7789, 1.6789)	-0.73	0.982
Sem 1500 - fez 500	-0.0167	0.0833	(-0.8929, 0.8596)	-0.20	1.000
lrc 500 - fez 1000	0.0500	0.0373	(-0.5029, 0.6029)	1.34	0.855
lrc 1000 - fez 1000	0.067	0.105	(-2.156, 2.289)	0.63	0.992
lrc 1500 - fez 1000	0.0500	0.0373	(-0.5029, 0.6029)	1.34	0.855
Sem 1500 - fez 1000	0.0833	0.0601	(-0.6042, 0.7709)	1.39	0.843
lrc 1000 - lrc 500	0.017	0.101	(-2.948, 2.982)	0.16	1.000
lrc 1500 - lrc 500	0.0000	0.0236	(-0.2257, 0.2257)	0.00	1.000
Sem 1500 - lrc 500	0.0333	0.0527	(-1.0780, 1.1447)	0.63	0.992
lrc 1500 - lrc 1000	-0.017	0.101	(-2.982, 2.948)	-0.16	1.000
Sem 1500 - lrc 1000	0.017	0.112	(-1.642, 1.675)	0.15	1.000
Sem 1500 - lrc 1500	0.0333	0.0527	(-1.0780, 1.1447)	0.63	0.992

One-Sample T: fez 500, fez 1000, Gus 1500, eGFP 1500, lrc 1500, Sem 1500, H2O

Descriptive Statistics

Sample	N	Mean	StDev	SE Mean	95% CI for μ
fez 500	2	0.1667	0.0943	0.0667	(-0.6804, 1.0137)
fez 1000	2	0.0667	0.0471	0.0333	(-0.3569, 0.4902)
Gus 1500	2	0.0667	0.0943	0.0667	(-0.7804, 0.9137)
eGFP 1500	2	0.031754	0.000713	0.000504	(0.025350, 0.038158)
lrc 1500	2	0.1167	0.0236	0.0167	(-0.0951, 0.3284)
Sem 1500	2	0.1500	0.0707	0.0500	(-0.4853, 0.7853)
H2O	2	0.0500	0.0236	0.0167	(-0.1618, 0.2618)

μ: population mean of fez 500, fez 1000, Gus 1500, eGFP 1500, lrc 1500, Sem 1500, H2O

Test

Null hypothesis	H_0: $\mu = 0.0333333$
Alternative hypothesis	H_1: $\mu \neq 0.0333333$

Sample	T-Value	P-Value
fez 500	2.00	0.295
fez 1000	1.00	0.500
Gus 1500	0.50	0.705
eGFP 1500	-3.13	0.197
lrc 1500	5.00	0.126
Sem 1500	2.33	0.258
H2O	1.00	0.500

Figure 7B

Descriptive Statistics – data in lethality

Column	Size	Missing	Mean	Std Dev	Std. Error	C.I. of Mean
Gus 1500	2	0	0,0333	0,0471	0,0333	0,424
fez2 1500	2	0	0,0517	0,0731	0,0517	0,657
sem1a 1500	2	0	0,0667	0,000	0,000	0,000
lrcE1 1500	2	0	0,0635	0,0427	0,0302	0,384

Column	Range	Max	Min	Median	25%	75%
Gus 1500	0,0667	0,0667	0,000	0,0333	0,000	0,0667
fez2 1500	0,103	0,103	0,000	0,0517	0,000	0,103
sem1a 1500	0,000	0,0667	0,0667	0,0667	0,0667	0,0667
lrcE1 1500	0,0604	0,0938	0,0333	0,0635	0,0333	0,0938

Column	Skewness	Kurtosis	K-S Dist.	K-S Prob.	SWilk W	SWilk Prob
Gus 1500	--	--	0,260	0,481	--	--
fez2 1500	--	--	0,260	0,481	--	--
sem1a 1500	--	--	0,000	<0,001	--	--
lrcE1 1500	--	--	0,260	0,481	--	--

Column	Sum	Sum of Squares
Gus 1500	0,0667	0,00444
fez2 1500	0,103	0,0107
sem1a 1500	0,133	0,00889
lrcE1 1500	0,127	0,00990

One Way Analysis of Variance

Normality Test (Shapiro-Wilk): Passed (P = 0,665)

Equal Variance Test (Brown-Forsythe): Failed (P < 0,050)

Test execution ended by user request, ANOVA on Ranks begun

Kruskal-Wallis One Way Analysis of Variance on Ranks

Group	N	Missing	Median	25%	75%
Gus 1500	2	0	0,0333	0,000	0,0667
fez2 1500	2	0	0,0517	0,000	0,103
sem1a 1500	2	0	0,0667	0,0667	0,0667
lrcE1 1500	2	0	0,0635	0,0333	0,0938

H = 0,753 with 3 degrees of freedom. P(est.)= 0,861 P(exact)= 0,886

The differences in the median values among the treatment groups are not great enough to exclude the possibility that the difference is due to random sampling variability; there is not a statistically significant difference (P = 0,886)

One-way ANOVA: Gus A dsRNA 1500, fez2 dsRNA 1500, lrc_E1 dsRNA 1500

Method

Null hypothesis	All means are equal
Alternative hypothesis	Not all means are equal
Significance level	α = 0.05

Equal variances were not assumed for the analysis.

Factor Information

Factor	Levels	Values
Factor	3	Gus A dsRNA 1500, fez2 dsRNA 1500, lrc_E1 dsRNA 1500

Welch's Test

Source	DF Num	DF Den	F-Value	P-Value
Factor	2	1.92818	0.17	0.857

Model Summary

R-sq	R-sq(adj)	R-sq(pred)
8.98%	0.00%	0.00%

Means

Factor	N	Mean	StDev	95% CI
Gus A dsRNA 1500	2	0.0333	0.0471	(-0.3902, 0.4569)
fez2 dsRNA 1500	2	0.0517	0.0731	(-0.6055, 0.7089)
lrc_E1 dsRNA 1500	2	0.0635	0.0427	(-0.3203, 0.4474)

Games-Howell Pairwise Comparisons

Grouping Information Using the Games-Howell Method and 95% Confidence

Factor	N	Mean	Grouping
lrc_E1 dsRNA 1500	2	0.0635	A
fez2 dsRNA 1500	2	0.0517	A
Gus A dsRNA 1500	2	0.0333	A

Means that do not share a letter are significantly different.

Games-Howell Simultaneous Tests for Differences of Means

Difference of Levels	Difference of Means	SE of Difference	95% CI	T-Value	Adjusted P-Value
fez2 dsRNA 1 - Gus A dsRNA	0.0184	0.0615	(-0.4189, 0.4557)	0.30	0.953
lrc_E1 dsRNA - Gus A dsRNA	0.0302	0.0450	(-0.2376, 0.2980)	0.67	0.801
lrc_E1 dsRNA - fez2 dsRNA 1	0.0118	0.0599	(-0.4490, 0.4726)	0.20	0.979

One-Sample T: Gus A dsRNA 1500, fez2 dsRNA 1500, lrc_E1 dsRNA 1500

Descriptive Statistics

Sample	N	Mean	StDev	SE Mean	95% CI for μ
Gus A dsRNA 1500	2	0.0333	0.0471	0.0333	(-0.3902, 0.4569)
fez2 dsRNA 1500	2	0.0517	0.0731	0.0517	(-0.6055, 0.7089)
lrc_E1 dsRNA 1500	2	0.0635	0.0427	0.0302	(-0.3203, 0.4474)

μ: population mean of Gus A dsRNA 1500, fez2 dsRNA 1500, lrc_E1 dsRNA 1500

Test

Null hypothesis	H_0: μ = 0.0666667
Alternative hypothesis	H_1: μ ≠ 0.0666667

Sample	T-Value	P-Value
Gus A dsRNA 1500	-1.00	0.500
fez2 dsRNA 1500	-0.29	0.821
lrc_E1 dsRNA 1500	-0.10	0.934

One-Sample T: fez2 dsRNA 1500, sem1a_E8 dsRNA 1500, lrc_E1 dsRNA 1500

Descriptive Statistics

Sample	N	Mean	StDev	SE Mean	95% CI for μ
fez2 dsRNA 1500	2	0.0517	0.0731	0.0517	(-0.6055, 0.7089)
sem1a_E8 dsRNA 1500	2	0.06667	0.00000	0.00000	(0.06667, 0.06667)
lrc_E1 dsRNA 1500	2	0.0635	0.0427	0.0302	(-0.3203, 0.4474)

μ: population mean of fez2 dsRNA 1500, sem1a_E8 dsRNA 1500, lrc_E1 dsRNA 1500

Test

Null hypothesis	H_0: $\mu = 0.03333$
Alternative hypothesis	H_1: $\mu \neq 0.03333$

Sample	T-Value	P-Value
fez2 dsRNA 1500	0.36	0.782
sem1a_E8 dsRNA 1500	*	*
lrc_E1 dsRNA 1500	1.00	0.500

*** NOTE * All values in column are identical.**

Figure 7C

Descriptive Statistics – data in lethality

Column	Size	Missing	Mean	Std Dev	Std. Error	C.I. of Mean
H2O dye	3	0	0,0533	0,0462	0,0267	0,115
H2O	3	0	0,133	0,0611	0,0353	0,152
ctl siRNA	3	0	0,240	0,183	0,106	0,455
fez2 siRNA	3	0	0,293	0,257	0,148	0,639
lrc siRNA	3	0	0,427	0,254	0,147	0,631
sem1a siRNA	3	0	0,333	0,180	0,104	0,448

Column	Range	Max	Min	Median	25%	75%
H2O dye	0,0800	0,0800	0,000	0,0800	0,000	0,0800
H2O	0,120	0,200	0,0800	0,120	0,0800	0,200
ctl siRNA	0,360	0,400	0,0400	0,280	0,0400	0,400
fez2 siRNA	0,480	0,480	0,000	0,400	0,000	0,480
lrc siRNA	0,440	0,720	0,280	0,280	0,280	0,720
sem1a siRNA	0,360	0,520	0,160	0,320	0,160	0,520

Column	Skewness	Kurtosis	K-S Dist.	K-S Prob.	SWilk W	SWilk Prob
H2O dye	-1,732	--	0,385	0,089	0,750	<0,001
H2O	0,935	--	0,253	0,487	0,964	0,637
ctl siRNA	-0,935	--	0,253	0,487	0,964	0,637
fez2 siRNA	-1,545	--	0,328	0,225	0,871	0,298
lrc siRNA	1,732	--	0,385	0,089	0,750	<0,001
sem1a siRNA	0,331	--	0,196	0,636	0,996	0,878

Column	Sum	Sum of Squares
H2O dye	0,160	0,0128
H2O	0,400	0,0608
ctl siRNA	0,720	0,240
fez2 siRNA	0,880	0,390
lrc siRNA	1,280	0,675
sem1a siRNA	1,000	0,398

One Way Analysis of Variance

Normality Test (Shapiro-Wilk): Passed (P = 0,980)

Equal Variance Test (Brown-Forsythe): Passed (P = 0,197)

Group Name	N	Missing	Mean	Std Dev	SEM
H2O dye	3	0	0,0533	0,0462	0,0267
H2O	3	0	0,133	0,0611	0,0353
ctl siRNA	3	0	0,240	0,183	0,106
fez2 siRNA	3	0	0,293	0,257	0,148
lrc siRNA	3	0	0,427	0,254	0,147
sem1a siRNA	3	0	0,333	0,180	0,104

Source of Variation	DF	SS	MS	F	P
Between Groups	5	0,277	0,0554	1,641	0,223
Residual	12	0,405	0,0338		
Total	17	0,682			

The differences in the mean values among the treatment groups are not great enough to exclude the possibility that the difference is due to random sampling variability; there is not a statistically significant difference (P = 0,223).

Power of performed test with alpha = 0,050: 0,165

The power of the performed test (0,165) is below the desired power of 0,800.
Less than desired power indicates you are less likely to detect a difference when one actually exists. Negative results should be interpreted cautiously.

Figure 8A

t-test

Normality Test (Shapiro-Wilk): Passed (P = 0,720)

Equal Variance Test (Brown-Forsythe): Failed (P < 0,050)

The result of the equal variance test indicates the likelihood that the two groups are sampled from populations with equal variances, but does not guarantee the equality or inequality of the two variances.

Group Name	N	Missing	Mean	Std Dev	SEM
control	8	0	0,955	0,0530	0,0187
dsx2 soaking	11	0	0,478	0,232	0,0700

Difference of means 0,477

Use the results of Welch's test, where equal variances are not assumed, if the equality of the population variances of the two groups is in doubt.

Equal Variances Assumed (Student's t-test):

t = 5,661 with 17 degrees of freedom.

95 percent two-tailed confidence interval for difference of means: 0,299 to 0,654

Two-tailed P-value = 0,0000282

The difference in the mean values of the two groups is greater than would be expected by chance; there is a statistically significant difference between the input groups (P = <0,001).

One-tailed P-value = 0,0000141

The sample mean of group control exceeds the sample mean of group dsx2 soaking by an amount that is greater than would be expected by chance, rejecting the hypothesis that the population mean of group dsx2 soaking is greater than or equal to the population mean of group control. (P = <0,001).

Equal Variances Not Assumed (Welch's t-test):

t = 6,580 with 11,403 degrees of freedom.

95 percent two-tailed confidence interval for difference of means: 0,318 to 0,635

Two-tailed P-value = 0,0000334

The difference in the mean values of the two groups is greater than would be expected by chance; there is a statistically significant difference between the input groups (P = <0,001).

One-tailed P-value = 0,0000167

The sample mean of group control exceeds the sample mean of group dsx2 soaking by an amount that is greater than would be expected by chance, rejecting the hypothesis that the population mean of group dsx2 soaking is greater than or equal to the population mean of group control. (P = <0,001).

Figure 8B

t-test

Normality Test (Shapiro-Wilk): Passed (P = 0,282)

Equal Variance Test (Brown-Forsythe): Passed (P = 0,540)

The result of the equal variance test indicates the likelihood that the two groups are sampled from populations with equal variances, but does not guarantee the equality or inequality of the two variances.

Group Name	N	Missing	Mean	Std Dev	SEM
male control	7	0	62,102	8,851	3,345
male dsx	5	0	61,021	5,551	2,482

Difference of means 1,081

Use the results of Welch's test, where equal variances are not assumed, if the equality of the population variances of the two groups is in doubt.

Equal Variances Assumed (Student's t-test):

t = 0,240 with 10 degrees of freedom.

95 percent two-tailed confidence interval for difference of means: -8,968 to 11,130

Two-tailed P-value = 0,815

The difference in the mean values of the two groups is not great enough to reject the possibility that the difference is due to random sampling variability. There is not a statistically significant difference between the input groups (P = 0,815).

One-tailed P-value = 0,408

The sample mean of group male control does not exceed the sample mean of the group male dsx by an amount great enough to exclude the possibility that the difference is due to random sampling variability. The hypothesis that the population mean of group male dsx is greater than or equal to the population mean of group male control cannot be rejected. (P = 0,408).

Equal Variances Not Assumed (Welch's t-test):

t = 0,260 with 9,917 degrees of freedom.

95 percent two-tailed confidence interval for difference of means: -8,211 to 10,373

Two-tailed P-value = 0,801

The difference in the mean values of the two groups is not great enough to reject the possibility that the difference is due to random sampling variability. There is not a statistically significant difference between the input groups (P = 0,801).

One-tailed P-value = 0,400

The sample mean of group male control does not exceed the sample mean of the group male dsx by an amount great enough to exclude the possibility that the difference is due to random sampling variability. The hypothesis that the population mean of group male dsx is greater than or equal to the population mean of group male control cannot be rejected. (P = 0,400).

Figure 9A

Descriptive Statistics – data in lethality

Column	Size	Missing	Mean	Std Dev	Std. Error	C.I. of Mean
H2O dye	3	0	0,458	0,114	0,0657	0,283
fez dsRNA	3	0	0,519	0,0757	0,0437	0,188
lrc dsRNA	3	0	0,523	0,0795	0,0459	0,198
sem1a dsRNA	3	0	0,451	0,114	0,0657	0,283
GusA dsRNA	3	0	0,504	0,118	0,0683	0,294
Coextract	3	0	0,492	0,123	0,0708	0,304
Handling	3	0	0,360	0,0739	0,0427	0,184

Column	Range	Max	Min	Median	25%	75%
H2O dye	0,216	0,545	0,330	0,500	0,330	0,545
fez dsRNA	0,136	0,568	0,432	0,557	0,432	0,568
lrc dsRNA	0,148	0,580	0,432	0,557	0,432	0,580
sem1a dsRNA	0,227	0,568	0,341	0,443	0,341	0,568
GusA dsRNA	0,227	0,636	0,409	0,466	0,409	0,636
Coextract	0,227	0,580	0,352	0,545	0,352	0,580
Handling	0,148	0,432	0,284	0,364	0,284	0,432

Column	Skewness	Kurtosis	K-S Dist.	K-S Prob.	SWilk W	SWilk Prob
H2O dye	-1,427	--	0,310	0,283	0,900	0,384
fez dsRNA	-1,688	--	0,358	0,142	0,812	0,144
lrc dsRNA	-1,574	--	0,333	0,210	0,862	0,274
sem1a dsRNA	0,298	--	0,193	0,640	0,997	0,890
GusA dsRNA	1,293	--	0,292	0,344	0,923	0,463
Coextract	-1,583	--	0,334	0,205	0,860	0,266
Handling	-0,230	--	0,187	0,646	0,998	0,915

Column	Sum	Sum of Squares
H2O dye	1,375	0,656
fez dsRNA	1,557	0,819
lrc dsRNA	1,568	0,832
sem1a dsRNA	1,352	0,635
GusA dsRNA	1,511	0,789
Coextract	1,477	0,757
Handling	1,080	0,399

One Way Analysis of Variance

Normality Test (Shapiro-Wilk): Passed (P = 0,136)

Equal Variance Test (Brown-Forsythe): Passed (P = 0,977)

Group Name	N	Missing	Mean	Std Dev	SEM
H2O dye	3	0	0,458	0,114	0,0657
fez dsRNA	3	0	0,519	0,0757	0,0437
lrc dsRNA	3	0	0,523	0,0795	0,0459
sem1a dsRNA	3	0	0,451	0,114	0,0657
GusA dsRNA	3	0	0,504	0,118	0,0683
Coextract	3	0	0,492	0,123	0,0708
Handling	3	0	0,360	0,0739	0,0427

Source of Variation	DF	SS	MS	F	P
Between Groups	6	0,0583	0,00971	0,938	0,499
Residual	14	0,145	0,0103		
Total	20	0,203			

The differences in the mean values among the treatment groups are not great enough to exclude the possibility that the difference is due to random sampling variability; there is not a statistically significant difference (P = 0,499).

Power of performed test with alpha = 0,050: 0,050

The power of the performed test (0,050) is below the desired power of 0,800.
Less than desired power indicates you are less likely to detect a difference when one actually exists. Negative results should be interpreted cautiously.

Figure 9B

Descriptive Statistics – data in lethality

Column	Size	Missing	Mean	Std Dev	Std. Error	C.I. of Mean
H2O dye	3	0	0,543	0,193	0,112	0,480
fez2 siRNA	3	0	0,663	0,0966	0,0558	0,240
lrc siRNA	3	0	0,678	0,181	0,105	0,450
sem1a siRNA	3	0	0,568	0,129	0,0745	0,321
ctl siRNA	3	0	0,572	0,202	0,117	0,502
H2O	3	0	0,458	0,106	0,0610	0,262
Handling	3	0	0,330	0,0227	0,0131	0,0565

Column	Range	Max	Min	Median	25%	75%
H2O dye	0,383	0,750	0,367	0,511	0,367	0,750
fez2 siRNA	0,182	0,773	0,591	0,625	0,591	0,773
lrc siRNA	0,352	0,830	0,477	0,727	0,477	0,830
sem1a siRNA	0,239	0,716	0,477	0,511	0,477	0,716
ctl siRNA	0,375	0,716	0,341	0,659	0,341	0,716
H2O	0,193	0,580	0,386	0,409	0,386	0,580
Handling	0,0455	0,352	0,307	0,330	0,307	0,352

Column	Skewness	Kurtosis	K-S Dist.	K-S Prob.	SWilk W	SWilk Prob
H2O dye	0,715	--	0,231	0,557	0,980	0,729
fez2 siRNA	1,493	--	0,319	0,251	0,885	0,339
lrc siRNA	-1,132	--	0,274	0,412	0,945	0,546
sem1a siRNA	1,597	--	0,337	0,197	0,855	0,253
ctl siRNA	-1,579	--	0,333	0,207	0,861	0,269
H2O	1,642	--	0,346	0,172	0,837	0,206
Handling	-0,0000000660	--	0,175	0,654	1,000	1,000

Column	Sum	Sum of Squares
H2O dye	1,629	0,959
fez2 siRNA	1,989	1,337
lrc siRNA	2,034	1,445
sem1a siRNA	1,705	1,002
ctl siRNA	1,716	1,063
H2O	1,375	0,653
Handling	0,989	0,327

One Way Analysis of Variance

Normality Test (Shapiro-Wilk): Passed (P = 0,574)

Equal Variance Test (Brown-Forsythe): Passed (P = 0,679)

Group Name	N	Missing	Mean	Std Dev	SEM
H2O dye	3	0	0,543	0,193	0,112
fez2 siRNA	3	0	0,663	0,0966	0,0558
lrc siRNA	3	0	0,678	0,181	0,105
sem1a siRNA	3	0	0,568	0,129	0,0745
ctl siRNA	3	0	0,572	0,202	0,117
H2O	3	0	0,458	0,106	0,0610
Handling	3	0	0,330	0,0227	0,0131

Source of Variation	DF	SS	MS	F	P
Between Groups	6	0,260	0,0434	2,043	0,127
Residual	14	0,297	0,0212		
Total	20	0,558			

The differences in the mean values among the treatment groups are not great enough to exclude the possibility that the difference is due to random sampling variability; there is not a statistically significant difference (P = 0,127).

Power of performed test with alpha = 0,050: 0,283

The power of the performed test (0,283) is below the desired power of 0,800.
Less than desired power indicates you are less likely to detect a difference when one actually exists. Negative results should be interpreted cautiously.

Figure 9C

Descriptive Statistics – data in lethality

Column	Size	Missing	Mean	Std Dev	Std. Error	C.I. of Mean
H2O dye	3	0	0,180	0,0500	0,0289	0,124
fez2 siRNA	3	0	0,217	0,107	0,0617	0,266
lrc siRNA	3	0	0,188	0,00703	0,00406	0,0175
sem1a siRNA	3	0	0,214	0,0913	0,0527	0,227
ctl siRNA	3	0	0,187	0,0153	0,00882	0,0379
H2O	3	0	0,148	0,0103	0,00597	0,0257
Handling	3	0	0,121	0,0186	0,0107	0,0462

Column	Range	Max	Min	Median	25%	75%
H2O dye	0,1000	0,230	0,130	0,180	0,130	0,230
fez2 siRNA	0,190	0,340	0,150	0,160	0,150	0,340
lrc siRNA	0,0135	0,194	0,180	0,190	0,180	0,194
sem1a siRNA	0,181	0,311	0,130	0,200	0,130	0,311
ctl siRNA	0,0300	0,200	0,170	0,190	0,170	0,200
H2O	0,0200	0,160	0,140	0,145	0,140	0,160
Handling	0,0372	0,140	0,103	0,120	0,103	0,140

Column	Skewness	Kurtosis	K-S Dist.	K-S Prob.	SWilk W	SWilk Prob
H2O dye	-2,498E-015	--	0,175	0,654	1,000	1,000
fez2 siRNA	1,715	--	0,369	0,120	0,789	0,089
lrc siRNA	-1,248	--	0,287	0,364	0,930	0,488
sem1a siRNA	0,660	--	0,226	0,571	0,983	0,751
ctl siRNA	-0,935	--	0,253	0,487	0,964	0,637
H2O	1,206	--	0,282	0,382	0,936	0,510
Handling	0,225	--	0,187	0,647	0,998	0,917

Column	Sum	Sum of Squares
H2O dye	0,540	0,102
fez2 siRNA	0,650	0,164
lrc siRNA	0,564	0,106
sem1a siRNA	0,641	0,154
ctl siRNA	0,560	0,105
H2O	0,445	0,0664
Handling	0,363	0,0446

One Way Analysis of Variance

Normality Test (Shapiro-Wilk): Failed (P < 0,050)

Test execution ended by user request, ANOVA on Ranks begun

Kruskal-Wallis One Way Analysis of Variance on Ranks

Group	N	Missing	Median	25%	75%
H2O dye	3	0	0,180	0,130	0,230
fez2 siRNA	3	0	0,160	0,150	0,340
lrc siRNA	3	0	0,190	0,180	0,194
sem1a siRNA	3	0	0,200	0,130	0,311
ctl siRNA	3	0	0,190	0,170	0,200
H2O	3	0	0,145	0,140	0,160
Handling	3	0	0,120	0,103	0,140

H = 8,861 with 6 degrees of freedom. (P = 0,182)

The differences in the median values among the treatment groups are not great enough to exclude the possibility that the difference is due to random sampling variability; there is not a statistically significant difference (P = 0,182)

Descriptive Statistics, transformed (arcsin transformation) data

Column	Size	Missing	Mean	Std Dev	Std. Error	C.I. of Mean
asinsqrt(col(1))	3	0	0,436	0,0657	0,0379	0,163
asinsqrt(col(2))	3	0	0,477	0,126	0,0728	0,313
asinsqrt(col(3))	3	0	0,448	0,00902	0,00521	0,0224
asinsqrt(col(4))	3	0	0,475	0,112	0,0646	0,278
asinsqrt(col(5))	3	0	0,447	0,0197	0,0114	0,0490
asinsqrt(col(6))	3	0	0,395	0,0145	0,00835	0,0359
asinsqrt(col(7))	3	0	0,355	0,0286	0,0165	0,0709

Column	Range	Max	Min	Median	25%	75%
asinsqrt(col(1))	0,131	0,500	0,369	0,438	0,369	0,500
asinsqrt(col(2))	0,225	0,623	0,398	0,412	0,398	0,623
asinsqrt(col(3))	0,0174	0,456	0,438	0,451	0,438	0,456
asinsqrt(col(4))	0,223	0,592	0,369	0,464	0,369	0,592
asinsqrt(col(5))	0,0387	0,464	0,425	0,451	0,425	0,464
asinsqrt(col(6))	0,0280	0,412	0,383	0,391	0,383	0,412
asinsqrt(col(7))	0,0571	0,383	0,326	0,354	0,326	0,383

Column	Skewness	Kurtosis	K-S Dist.	K-S Prob.	SWilk W	SWilk Prob
asinsqrt(col(1))	-0,165	--	0,181	0,651	0,999	0,939
asinsqrt(col(2))	1,709	--	0,366	0,126	0,796	0,105
asinsqrt(col(3))	-1,259	--	0,288	0,359	0,928	0,482
asinsqrt(col(4))	0,442	--	0,206	0,619	0,993	0,836
asinsqrt(col(5))	-0,968	--	0,256	0,475	0,961	0,622
asinsqrt(col(6))	1,183	--	0,279	0,391	0,938	0,521
asinsqrt(col(7))	0,126	--	0,178	0,653	0,999	0,953

Column	Sum	Sum of Squares
asinsqrt(col(1))	1,307	0,578
asinsqrt(col(2))	1,432	0,715
asinsqrt(col(3))	1,345	0,603
asinsqrt(col(4))	1,424	0,701
asinsqrt(col(5))	1,340	0,599
asinsqrt(col(6))	1,186	0,470
asinsqrt(col(7))	1,064	0,379

One Way Analysis of Variance

Normality Test (Shapiro-Wilk): Passed (P = 0,082)

Equal Variance Test (Brown-Forsythe): Passed (P = 0,327)

Group Name	N	Missing	Mean	Std Dev	SEM
asinsqrt(col(1))	3	0	0,436	0,0657	0,0379
asinsqrt(col(2))	3	0	0,477	0,126	0,0728
asinsqrt(col(3))	3	0	0,448	0,00902	0,00521
asinsqrt(col(4))	3	0	0,475	0,112	0,0646
asinsqrt(col(5))	3	0	0,447	0,0197	0,0114
asinsqrt(col(6))	3	0	0,395	0,0145	0,00835
asinsqrt(col(7))	3	0	0,355	0,0286	0,0165

Source of Variation	DF	SS	MS	F	P
Between Groups	6	0,0351	0,00584	1,196	0,363
Residual	14	0,0684	0,00488		
Total	20	0,103			

The differences in the mean values among the treatment groups are not great enough to exclude the possibility that the difference is due to random sampling variability; there is not a statistically significant difference (P = 0,363).

Power of performed test with alpha = 0,050: 0,084

The power of the performed test (0,084) is below the desired power of 0,800.
Less than desired power indicates you are less likely to detect a difference when one actually exists. Negative results should be interpreted cautiously.

Figure 9D

One Way Analysis of Variance

Normality Test (Shapiro-Wilk): Passed (P = 0,443)

Equal Variance Test (Brown-Forsythe): Failed (P < 0,050)

**Group Name**	**N**	**Missing**	**Mean**	**Std Dev**	**SEM**
fez2 dsRNA	2	0	0,883	0,0130	0,00920
fez2 h2o+dye	2	0	0,775	0,369	0,261
fez2 coextract	2	0	0,728	0,310	0,219

**Source of Variation**	**DF**	**SS**	**MS**	**F**	**P**
Between Groups	2	0,0254	0,0127	0,164	0,856
Residual	3	0,232	0,0774		
Total	5	0,258			

The differences in the mean values among the treatment groups are not great enough to exclude the possibility that the difference is due to random sampling variability; there is not a statistically significant difference (P = 0,856).

Power of performed test with alpha = 0,050: 0,050

The power of the performed test (0,050) is below the desired power of 0,800.
Less than desired power indicates you are less likely to detect a difference when one actually exists. Negative results should be interpreted cautiously.

One-way ANOVA: dsRNA fez2, ddH2O + dye fez2, Coextract empty bact fez2

Method

Null hypothesis	All means are equal
Alternative hypothesis	Not all means are equal
Significance level	α = 0.05

Equal variances were not assumed for the analysis.

Factor Information

Factor	Levels	Values
Factor	3	dsRNA fez2, ddH2O + dye fez2, Coextract empty bact fez2

Welch's Test

Source	DF Num	DF Den	F-Value	P-Value
Factor	2	1.33734	0.23	0.824

Model Summary

R-sq	R-sq(adj)	R-sq(pred)
9.88%	0.00%	0.00%

Means

Factor	N	Mean	StDev	95% CI
dsRNA fez2	2	0.88332	0.01301	(0.76640, 1.00024)
ddH2O + dye fez2	2	0.775	0.369	(-2.537, 4.086)
Coextract empty bact fez2	2	0.728	0.310	(-2.057, 3.513)

Games-Howell Pairwise Comparisons

Grouping Information Using the Games-Howell Method and 95% Confidence

Factor	N	Mean	Grouping
dsRNA fez2	2	0.88332	A
ddH2O + dye fez2	2	0.775	A
Coextract empty bact fez2	2	0.728	A

Means that do not share a letter are significantly different.

One Way Analysis of Variance

Normality Test (Shapiro-Wilk): Passed (P = 0,841)

Equal Variance Test (Brown-Forsythe): Failed (P < 0,050)

Group Name	N	Missing	Mean	Std Dev	SEM
lrc dsRNA	2	0	1,239	0,596	0,421
lrc h2o+dye	2	0	1,035	0,127	0,0896
lrc coextrct	2	0	1,023	0,141	0,0994

Source of Variation	DF	SS	MS	F	P
Between Groups	2	0,0589	0,0295	0,226	0,810
Residual	3	0,391	0,130		
Total	5	0,449			

The differences in the mean values among the treatment groups are not great enough to exclude the possibility that the difference is due to random sampling variability; there is not a statistically significant difference (P = 0,810).

Power of performed test with alpha = 0,050: 0,050

The power of the performed test (0,050) is below the desired power of 0,800.
Less than desired power indicates you are less likely to detect a difference when one actually exists. Negative results should be interpreted cautiously.

One-way ANOVA: dsRNA lrc, ddH2O + dye lrc, Coextract empty bact lrc

Method

Null hypothesis	All means are equal
Alternative hypothesis	Not all means are equal
Significance level	α = 0.05

Equal variances were not assumed for the analysis.

Factor Information

Factor	Levels	Values
Factor	3	dsRNA lrc, ddH2O + dye lrc, Coextract empty bact lrc

Welch's Test

Source	DF Num	DF Den	F-Value	P-Value
Factor	2	1.79989	0.09	0.917

Model Summary

R-sq	R-sq(adj)	R-sq(pred)
13.11%	0.00%	0.00%

Means

Factor	N	Mean	StDev	95% CI
dsRNA lrc	2	1.239	0.596	(-4.112, 6.590)
ddH2O + dye lrc	2	1.0350	0.1268	(-0.1040, 2.1740)
Coextract empty bact lrc	2	1.0231	0.1406	(-0.2402, 2.2863)

Games-Howell Pairwise Comparisons

Grouping Information Using the Games-Howell Method and 95% Confidence

Factor	N	Mean	Grouping
dsRNA lrc	2	1.239	A
ddH2O + dye lrc	2	1.0350	A
Coextract empty bact lrc	2	1.0231	A

Means that do not share a letter are significantly different.

One Way Analysis of Variance

Normality Test (Shapiro-Wilk): Passed (P = 0,969)

Equal Variance Test (Brown-Forsythe): Failed (P < 0,050)

Group Name	N	Missing	Mean	Std Dev	SEM
sem1a dsRNA	2	0	0,822	0,0215	0,0152
sem1a h2o+dye	2	0	1,237	0,242	0,171
sem1a coextract	2	0	1,289	0,432	0,306

Source of Variation	DF	SS	MS	F	P
Between Groups	2	0,262	0,131	1,599	0,337
Residual	3	0,246	0,0819		
Total	5	0,508			

The differences in the mean values among the treatment groups are not great enough to exclude the possibility that the difference is due to random sampling variability; there is not a statistically significant difference (P = 0,337).

Power of performed test with alpha = 0,050: 0,090

The power of the performed test (0,090) is below the desired power of 0,800.
Less than desired power indicates you are less likely to detect a difference when one actually exists. Negative results should be interpreted cautiously.

One-way ANOVA: dsRNA sem1a, ddH2O + dye sem1a, Coextract empty bact sem1a

Method

Null hypothesis	All means are equal
Alternative hypothesis	Not all means are equal
Significance level	α = 0.05

Equal variances were not assumed for the analysis.

Factor Information

Factor	Levels	Values
Factor	3	dsRNA sem1a, ddH2O + dye sem1a, Coextract empty bact sem1a

Welch's Test

Source	DF Num	DF Den	F-Value	P-Value
Factor	2	1.34707	2.72	0.337

Model Summary

R-sq	R-sq(adj)	R-sq(pred)
51.59%	19.32%	0.00%

Means

Factor	N	Mean	StDev	95% CI
dsRNA sem1a	2	0.8222	0.0215	(0.6289, 1.0156)
ddH2O + dye sem1a	2	1.237	0.242	(-0.937, 3.411)
Coextract empty bact sem1a	2	1.289	0.432	(-2.593, 5.172)

Games-Howell Pairwise Comparisons

Grouping Information Using the Games-Howell Method and 95% Confidence

Factor	N	Mean	Grouping
Coextract empty bact sem1a	2	1.289	A
ddH2O + dye sem1a	2	1.237	A
dsRNA sem1a	2	0.8222	A

Means that do not share a letter are significantly different.

Figure 9E

One Way Analysis of Variance

Normality Test (Shapiro-Wilk): Passed (P = 0,551)

Equal Variance Test (Brown-Forsythe): Failed (P < 0,050)

Group Name	N	Missing	Mean	Std Dev	SEM
fez2 siRNA	2	0	1,231	0,243	0,172
fez2 h2o+dye	2	0	1,317	0,200	0,141
fez2 h2o	2	0	1,165	0,0413	0,0292

Source of Variation	DF	SS	MS	F	P
Between Groups	2	0,0234	0,0117	0,349	0,731
Residual	3	0,101	0,0335		
Total	5	0,124			

The differences in the mean values among the treatment groups are not great enough to exclude the possibility that the difference is due to random sampling variability; there is not a statistically significant difference (P = 0,731).

Power of performed test with alpha = 0,050: 0,050

The power of the performed test (0,050) is below the desired power of 0,800.
Less than desired power indicates you are less likely to detect a difference when one actually exists. Negative results should be interpreted cautiously.

One-way ANOVA: siRNA fez2, ddH2O + dye fez2, ddH2O fez2

Method

Null hypothesis	All means are equal
Alternative hypothesis	Not all means are equal
Significance level	α = 0.05

Equal variances were not assumed for the analysis.

Factor Information

Factor	Levels	Values
Factor	3	siRNA fez2, ddH2O + dye fez2, ddH2O fez2

Welch's Test

Source	DF Num	DF Den	F-Value	P-Value
Factor	2	1.42374	0.42	0.719

Model Summary

R-sq	R-sq(adj)	R-sq(pred)
18.86%	0.00%	0.00%

Means

Factor	N	Mean	StDev	95% CI
siRNA fez2	2	1.231	0.243	(-0.951, 3.412)
ddH2O + dye fez2	2	1.317	0.200	(-0.479, 3.114)
ddH2O fez2	2	1.1648	0.0413	(0.7935, 1.5361)

Games-Howell Pairwise Comparisons

Grouping Information Using the Games-Howell Method and 95% Confidence

Factor	N	Mean	Grouping
ddH2O + dye fez2	2	1.317	A
siRNA fez2	2	1.231	A
ddH2O fez2	2	1.1648	A

Means that do not share a letter are significantly different.

One Way Analysis of Variance

Normality Test (Shapiro-Wilk): Passed (P = 0,259)

Equal Variance Test (Brown-Forsythe): Failed (P < 0,050)

Group Name	N	Missing	Mean	Std Dev	SEM
lrc siRNA	2	0	1,199	0,413	0,292
lrc h2o+dye	2	0	1,213	0,398	0,282
lrc h2o	2	0	1,198	0,111	0,0782

Source of Variation	DF	SS	MS	F	P
Between Groups	2	0,000288	0,000144	0,00127	0,999
Residual	3	0,341	0,114		
Total	5	0,341			

The differences in the mean values among the treatment groups are not great enough to exclude the possibility that the difference is due to random sampling variability; there is not a statistically significant difference (P = 0,999).

Power of performed test with alpha = 0,050: 0,050

The power of the performed test (0,050) is below the desired power of 0,800.
Less than desired power indicates you are less likely to detect a difference when one actually exists. Negative results should be interpreted cautiously.

One-way ANOVA: siRNA lrc, ddH2O + dye lrc, ddH2O lrc

Method

Null hypothesis	All means are equal
Alternative hypothesis	Not all means are equal
Significance level	α = 0.05

Equal variances were not assumed for the analysis.

Factor Information

Factor	Levels	Values
Factor	3	siRNA lrc, ddH2O + dye lrc, ddH2O lrc

Welch's Test

Source	DF Num	DF Den	F-Value	P-Value
Factor	2	1.51017	0.00	0.999

Model Summary

R-sq	R-sq(adj)	R-sq(pred)
0.08%	0.00%	0.00%

Means

Factor	N	Mean	StDev	95% CI
siRNA lrc	2	1.199	0.413	(-2.509, 4.907)
ddH2O + dye lrc	2	1.213	0.398	(-2.364, 4.790)
ddH2O lrc	2	1.1982	0.1106	(0.2043, 2.1921)

Games-Howell Pairwise Comparisons

Grouping Information Using the Games-Howell Method and 95% Confidence

Factor	N	Mean	Grouping
ddH2O + dye lrc	2	1.213	A
siRNA lrc	2	1.199	A
ddH2O lrc	2	1.1982	A

Means that do not share a letter are significantly different.

One Way Analysis of Variance

Normality Test (Shapiro-Wilk): Passed (P = 0,154)

Equal Variance Test (Brown-Forsythe): Failed (P < 0,050)

Group Name	N	Missing	Mean	Std Dev	SEM
sem1a siRNA	2	0	1,058	0,289	0,204
sem1 h2o+dye	2	0	0,859	0,180	0,127
sem1a h2o	2	0	1,049	0,211	0,149

Source of Variation	DF	SS	MS	F	P
Between Groups	2	0,0504	0,0252	0,472	0,664
Residual	3	0,160	0,0534		
Total	5	0,211			

The differences in the mean values among the treatment groups are not great enough to exclude the possibility that the difference is due to random sampling variability; there is not a statistically significant difference (P = 0,664).

Power of performed test with alpha = 0,050: 0,050

The power of the performed test (0,050) is below the desired power of 0,800.
Less than desired power indicates you are less likely to detect a difference when one actually exists. Negative results should be interpreted cautiously.

One-way ANOVA: siRNA sem1a, ddH2O + dye sem1a, ddH2O sem1a

Method

Null hypothesis	All means are equal
Alternative hypothesis	Not all means are equal
Significance level	α = 0.05

Equal variances were not assumed for the analysis.

Factor Information

Factor	Levels	Values
Factor	3	siRNA sem1a, ddH2O + dye sem1a, ddH2O sem1a

Welch's Test

Source	DF Num	DF Den	F-Value	P-Value
Factor	2	1.93831	0.45	0.690

Model Summary

R-sq	R-sq(adj)	R-sq(pred)
23.93%	0.00%	0.00%

Means

Factor	N	Mean	StDev	95% CI
siRNA sem1a	2	1.058	0.289	(-1.537, 3.653)
ddH2O + dye sem1a	2	0.859	0.180	(-0.754, 2.473)
ddH2O sem1a	2	1.049	0.211	(-0.849, 2.948)

Games-Howell Pairwise Comparisons

Grouping Information Using the Games-Howell Method and 95% Confidence

Factor	N	Mean	Grouping
siRNA sem1a	2	1.058	A
ddH2O sem1a	2	1.049	A
ddH2O + dye sem1a	2	0.859	A

Means that do not share a letter are significantly different.

Figure 10

One Way Analysis of Variance

Normality Test (Kolmogorov-Smirnov): Passed (P = 0,064)

Equal Variance Test (Brown-Forsythe): Failed (P < 0,050)

Group Name	N	Missing	Mean	Std Dev	SEM
h2o+bact ctrl	11	0	1,118	0,540	0,163
weak dsRNA	21	0	0,324	0,201	0,0439
strong dsRNA	6	0	0,380	0,163	0,0665

Source of Variation	DF	SS	MS	F	P
Between Groups	2	4,793	2,396	21,756	<0,001
Residual	35	3,855	0,110		
Total	37	8,648			

The differences in the mean values among the treatment groups are greater than would be expected by chance; there is a statistically significant difference (P = <0,001).

Power of performed test with alpha = 0,050: 1,000

Statistics

Variable	Total Count	Mean	SE Mean	StDev	Variance	CoefVar	Minimum	Median	Maximum
dsRNA weak GFP	21	0.324324	0.0442844	0.202937	0.0411833	62.57	0.0320301	0.257507	0.804121
dsRNA strong GFP	21	0.379385	0.0655798	0.160637	0.0258043	42.34	0.241042	0.350258	0.675468
ddH2O and bact ctrl	21	1.11952	0.162687	0.539571	0.291136	48.20	0.423641	0.943620	1.89582

Sheet1

One-way ANOVA: dsRNA weak GFP, dsRNA strong GFP, ddH2O and bact ctrl

Method

Null hypothesis	All means are equal
Alternative hypothesis	Not all means are equal
Significance level	α = 0.05
Rows unused	25

Equal variances were not assumed for the analysis.

Factor Information

Factor	Levels	Values
Factor	3	dsRNA weak GFP, dsRNA strong GFP, ddH2O and bact ctrl

Welch’s Test

Source	DF Num	DF Den	F-Value	P-Value
Factor	2	13.6678	10.60	0.002

Model Summary

R-sq	R-sq(adj)	R-sq(pred)
55.43%	52.88%	46.75%

Means

Factor	N	Mean	StDev	95% CI
dsRNA weak GFP	21	0.3243	0.2029	(0.2319, 0.4167)
dsRNA strong GFP	6	0.3794	0.1606	(0.2108, 0.5480)
ddH2O and bact ctrl	11	1.120	0.540	(0.757, 1.482)

Games-Howell Pairwise Comparisons

Grouping Information Using the Games-Howell Method and 95% Confidence

Factor	N	Mean	Grouping	
ddH2O and bact ctrl	11	1.120	A	
dsRNA strong GFP	6	0.3794		B
dsRNA weak GFP	21	0.3243		B

Means that do not share a letter are significantly different.

Games-Howell Simultaneous Tests for Differences of Means

Difference of Levels	Difference of Means	SE of Difference	95% CI	T-Value	Adjusted P-Value
dsRNA strong - dsRNA weak G	0.0551	0.0791	(-0.1615, 0.2716)	0.70	0.771
ddH2O and ba - dsRNA weak G	0.795	0.169	(0.342, 1.248)	4.72	0.002
ddH2O and ba - dsRNA strong	0.740	0.175	(0.276, 1.204)	4.22	0.003

Figure S1

One-Sample t-test_qPCR yeast feeding assays, relative expression ratio

Normality Test (Kolmogorov-Smirnov): Passed (P = 0,481)

Group Name	N	Missing	Mean	Std Dev	SEM
Exp 1a_sem1a	2	0	1,120	0,581	0,411

Hypothesized population mean 1,000

t = 0,293 with 1 degrees of freedom.

95 percent two-tailed confidence interval for the population mean: -4,097 to 6,337

Two-tailed P-value = 0,818

The difference between the mean of the sampled population and the hypothesized population mean is not great enough to reject the hypothesis that the difference is only due to random sample variability. There is not a significant difference between the two means (P = 0,818).

One-tailed P-value = 0,409

The sample mean of the group does not exceed the hypothesized mean by an amount great enough to exclude the possibility that the difference is due to random sampling variability. The hypothesis that the hypothesized mean is greater than or equal to the true mean cannot be rejected. (P = 0,409).

One-Sample t-test_qPCR yeast feeding assays, relative expression ratio

Normality Test (Kolmogorov-Smirnov): Passed (P = 0,481)

Group Name	N	Missing	Mean	Std Dev	SEM
Exp 1b_sem1a	2	0	0,998	0,122	0,0863

Hypothesized population mean 1,000

t = -0,0276 with 1 degrees of freedom.

95 percent two-tailed confidence interval for the population mean: -0,0987 to 2,094

Two-tailed P-value = 0,982

The difference between the mean of the sampled population and the hypothesized population mean is not great enough to reject the hypothesis that the difference is only due to random sample variability. There is not a significant difference between the two means (P = 0,982).

One-tailed P-value = 0,491

The hypothesized mean does not exceed the sample mean of the group by an amount great enough to exclude the possibility that the difference is due to random sampling variability. The hypothesis that the true mean is greater than or equal to the hypothesized mean cannot be rejected. (P = 0,491).

One-Sample t-test_qPCR yeast feeding assays, relative expression ratio

Normality Test (Kolmogorov-Smirnov): Passed (P = 0,481)

**Group Name**	**N**	**Missing**	**Mean**	**Std Dev**	**SEM**
Exp 2_sem1a	2	0	0,761	0,00378	0,00267

Hypothesized population mean 1,000

t = -89,267 with 1 degrees of freedom.

95 percent two-tailed confidence interval for the population mean: 0,727 to 0,795

Two-tailed P-value = 0,00713

There is a statistically significant difference between the mean of the sampled population and the hypothesized population mean (P = 0,007).

One-tailed P-value = 0,00357

The hypothesized mean exceeds the sample mean of the group by an amount that is greater than would be expected by chance, rejecting the hypothesis that the true mean of group is greater than or equal to the hypothesized mean. (P = 0,004).

One-Sample t-test_qPCR yeast feeding assays, relative expression ratio

Normality Test (Kolmogorov-Smirnov): Passed (P = 0,481)

**Group Name**	**N**	**Missing**	**Mean**	**Std Dev**	**SEM**
Exp 3_sem1a	2	0	0,534	0,116	0,0817

Hypothesized population mean 1,000

t = -5,697 with 1 degrees of freedom.

95 percent two-tailed confidence interval for the population mean: -0,504 to 1,573

Two-tailed P-value = 0,111

The difference between the mean of the sampled population and the hypothesized population mean is not great enough to reject the hypothesis that the difference is only due to random sample variability. There is not a significant difference between the two means (P = 0,111).

One-tailed P-value = 0,0553

The hypothesized mean does not exceed the sample mean of the group by an amount great enough to exclude the possibility that the difference is due to random sampling variability. The hypothesis that the true mean is greater than or equal to the hypothesized mean cannot be rejected. (P = 0,055).

One-Sample t-test_qPCR yeast feeding assays, relative expression ratio

Normality Test (Kolmogorov-Smirnov): Passed (P = 0,481)

Group Name	N	Missing	Mean	Std Dev	SEM
Exp 3 fez2	2	0	0,906	0,148	0,104

Hypothesized population mean 1,000

t = -0,899 with 1 degrees of freedom.

95 percent two-tailed confidence interval for the population mean: -0,420 to 2,233

Two-tailed P-value = 0,534

The difference between the mean of the sampled population and the hypothesized population mean is not great enough to reject the hypothesis that the difference is only due to random sample variability. There is not a significant difference between the two means (P = 0,534).

One-tailed P-value = 0,267

The hypothesized mean does not exceed the sample mean of the group by an amount great enough to exclude the possibility that the difference is due to random sampling variability. The hypothesis that the true mean is greater than or equal to the hypothesized mean cannot be rejected. (P = 0,267).

One-Sample t-test_qPCR yeast feeding assays, relative expression ratio

Normality Test (Kolmogorov-Smirnov): Passed (P = 0,481)

Group Name	N	Missing	Mean	Std Dev	SEM
Exp 3 lrc	2	0	0,562	0,0320	0,0226

Hypothesized population mean 1,000

t = -19,345 with 1 degrees of freedom.

95 percent two-tailed confidence interval for the population mean: 0,275 to 0,850

Two-tailed P-value = 0,0329

There is a statistically significant difference between the mean of the sampled population and the hypothesized population mean (P = 0,033).

One-tailed P-value = 0,0164

The hypothesized mean exceeds the sample mean of the group by an amount that is greater than would be expected by chance, rejecting the hypothesis that the true mean of group is greater than or equal to the hypothesized mean. (P = 0,016).

t-test_ Survival numbers yeast feeding assays

Normality Test (Shapiro-Wilk): Passed (P = 0,683)

Equal Variance Test (Brown-Forsythe): Failed (P < 0,050)

The result of the equal variance test indicates the likelihood that the two groups are sampled from populations with equal variances, but does not guarantee the equality or inequality of the two variances.

Group Name	N	Missing	Mean	Std Dev	SEM
Exp 1a_Sem-1a	2	0	0,950	0,000	0,000
Exp 1a_control	2	0	0,825	0,0354	0,0250

Difference of means 0,125

Use the results of Welch's test, where equal variances are not assumed, if the equality of the population variances of the two groups is in doubt.

Equal Variances Assumed (Student's t-test):

t = 5,000 with 2 degrees of freedom.

95 percent two-tailed confidence interval for difference of means: 0,0174 to 0,233

Two-tailed P-value = 0,0377

The difference in the mean values of the two groups is greater than would be expected by chance; there is a statistically significant difference between the input groups (P = 0,038).

One-tailed P-value = 0,0189

The sample mean of group Exp 1a_Sem-1a exceeds the sample mean of group Exp 1a_control by an amount that is greater than would be expected by chance, rejecting the hypothesis that the population mean of group Exp 1a_control is greater than or equal to the population mean of group Exp 1a_Sem-1a. (P = 0,019).

Equal Variances Not Assumed (Welch's t-test):

t = 5,000 with 1,000 degrees of freedom.

95 percent two-tailed confidence interval for difference of means: -0,193 to 0,443

Two-tailed P-value = 0,126

The difference in the mean values of the two groups is not great enough to reject the possibility that the difference is due to random sampling variability. There is not a statistically significant difference between the input groups (P = 0,126).

One-tailed P-value = 0,0628

The sample mean of group Exp 1a_Sem-1a does not exceed the sample mean of the group Exp 1a_control by an amount great enough to exclude the possibility that the difference is due to random sampling variability. The hypothesis that the population mean of group Exp 1a_control is greater than or equal to the population mean of group Exp 1a_Sem-1a cannot be rejected. (P = 0,063).

One-Sample t-test_ Survival numbers yeast feeding assays

Normality Test (Kolmogorov-Smirnov): Failed (P < 0,050)

Test execution ended by user request, One-Sample Signed Rank Test begun

One-Sample Signed Rank Test

Group	N	Missing	Median	25%	75%
Exp 1b_Sem-1a	2,000	0,000	1,000	1,000	1,000

Hypothesized population median 1,000

W= 0,000 T+ = 0,000 T-= 0,000
Z-Statistic (based on positive ranks) = 6,790E-313

Yates continuity correction option applied to calculations.

P(est.)= 1,000 P(exact)= 1,000

95 percent confidence interval for the population median: 1,000 to 1,000

The difference between the median of the group and the hypothesized population median is not great enough to reject the possibility that the difference is due to random sampling variability.There is not a significant difference between the two medians (P = 1,000).

One-Sample t-test

Normality Test (Kolmogorov-Smirnov): Failed (P < 0,050)

Test execution ended by user request, One-Sample Signed Rank Test begun

One-Sample Signed Rank Test

Group	N	Missing	Median	25%	75%
Exp 1b_control	2,000	0,000	0,950	0,950	0,950

Hypothesized population median 1,000

W= -3,000 T+ = 0,000 T-= 3,000
Z-Statistic (based on positive ranks) = -1,414

Yates continuity correction option applied to calculations.

P(est.)= 0,346 P(exact)= 0,500

95 percent confidence interval for the population median: 0,950 to 0,950

The difference between the median of the group and the hypothesized population median is not great enough to reject the possibility that the difference is due to random sampling variability.There is not a significant difference between the two medians (P = 0,500).

Wilcoxon Signed Rank Test: Exp 1b_Sem-1a

Method

η: median of Exp 1b_Sem-1a

Descriptive Statistics

Sample	N	Median
Exp 1b_Sem-1a	2	1

Test

Null hypothesis	H_0: $\eta = 0.95$
Alternative hypothesis	H_1: $\eta \neq 0.95$

Sample	N for Test	Wilcoxon Statistic	P-Value
Exp 1b_Sem-1a	2	3.00	0.371

t-test_ Survival numbers yeast feeding assays

Normality Test (Shapiro-Wilk): Failed (P < 0,050)

Equal Variance Test (Brown-Forsythe): Passed (P = 1,000)

The result of the equal variance test indicates the likelihood that the two groups are sampled from populations with equal variances, but does not guarantee the equality or inequality of the two variances.

Group Name	N	Missing	Mean	Std Dev	SEM
Exp 2_Sem-1a	2	0	0,875	0,0354	0,0250
Exp 2_control	2	0	0,975	0,0354	0,0250

Difference of means -0,1000

Use the results of Welch's test, where equal variances are not assumed, if the equality of the population variances of the two groups is in doubt.

Equal Variances Assumed (Student's t-test):

t = -2,828 with 2 degrees of freedom.

95 percent two-tailed confidence interval for difference of means: -0,252 to 0,0521

Two-tailed P-value = 0,106

The difference in the mean values of the two groups is not great enough to reject the possibility that the difference is due to random sampling variability. There is not a statistically significant difference between the input groups (P = 0,106).

One-tailed P-value = 0,0528

The sample mean of group Exp 2_control does not exceed the sample mean of the group Exp 2_Sem-1a by an amount great enough to exclude the possibility that the difference is due to random sampling variability. The hypothesis that the population mean of group Exp 2_Sem-1a is greater than or equal to the population mean of group Exp 2_control cannot be rejected. (P = 0,053).

Equal Variances Not Assumed (Welch's t-test):

t = -2,828 with 2,000 degrees of freedom.

95 percent two-tailed confidence interval for difference of means: -0,252 to 0,0521

Two-tailed P-value = 0,106

The difference in the mean values of the two groups is not great enough to reject the possibility that the difference is due to random sampling variability. There is not a statistically significant difference between the input groups (P = 0,106).

One-tailed P-value = 0,0528

The sample mean of group Exp 2_control does not exceed the sample mean of the group Exp 2_Sem-1a by an amount great enough to exclude the possibility that the difference is due to random sampling variability. The hypothesis that the population mean of group Exp 2_Sem-1a is greater than or equal to the population mean of group Exp 2_control cannot be rejected. (P = 0,053).

t-test_ Survival numbers yeast feeding assays

Normality Test (Shapiro-Wilk): Passed (P = 0,969)

Equal Variance Test (Brown-Forsythe): Failed (P < 0,050)

The result of the equal variance test indicates the likelihood that the two groups are sampled from populations with equal variances, but does not guarantee the equality or inequality of the two variances.

Group Name	N	Missing	Mean	Std Dev	SEM
asinsqrt(col(9))	2	0	1,211	0,0537	0,0380
asinsqrt(col(10))	2	0	1,458	0,159	0,113

Difference of means -0,247

Use the results of Welch's test, where equal variances are not assumed, if the equality of the population variances of the two groups is in doubt.

Equal Variances Assumed (Student's t-test):

t = -2,076 with 2 degrees of freedom.

95 percent two-tailed confidence interval for difference of means: -0,759 to 0,265

Two-tailed P-value = 0,174

The difference in the mean values of the two groups is not great enough to reject the possibility that the difference is due to random sampling variability. There is not a statistically significant difference between the input groups (P = 0,174).

One-tailed P-value = 0,0868

The sample mean of group asinsqrt(col(10)) does not exceed the sample mean of the group asinsqrt(col(9)) by an amount great enough to exclude the possibility that the difference is due to random sampling variability. The hypothesis that the population mean of group asinsqrt(col(9)) is greater than or equal to the population mean of group asinsqrt(col(10)) cannot be rejected. (P = 0,087).

Equal Variances Not Assumed (Welch's t-test):

t = -2,076 with 1,224 degrees of freedom.

95 percent two-tailed confidence interval for difference of means: -1,759 to 1,265

Two-tailed P-value = 0,248

The difference in the mean values of the two groups is not great enough to reject the possibility that the difference is due to random sampling variability. There is not a statistically significant difference between the input groups (P = 0,248).

One-tailed P-value = 0,124

The sample mean of group asinsqrt(col(10)) does not exceed the sample mean of the group asinsqrt(col(9)) by an amount great enough to exclude the possibility that the difference is due to random sampling variability. The hypothesis that the population mean of group asinsqrt(col(9)) is greater than or equal to the population mean of group asinsqrt(col(10)) cannot be rejected. (P = 0,124).

t-test_ Survival numbers yeast feeding assays

Normality Test (Shapiro-Wilk): Passed (P = 0,714)

Equal Variance Test (Brown-Forsythe): Failed (P < 0,050)

The result of the equal variance test indicates the likelihood that the two groups are sampled from populations with equal variances, but does not guarantee the equality or inequality of the two variances.

Group Name	N	Missing	Mean	Std Dev	SEM
Exp 3_sem-1a	2	0	0,925	0,0354	0,0250
Exp 3_control	2	0	0,950	0,0707	0,0500

Difference of means -0,0250

Use the results of Welch's test, where equal variances are not assumed, if the equality of the population variances of the two groups is in doubt.

Equal Variances Assumed (Student's t-test):

t = -0,447 with 2 degrees of freedom.

95 percent two-tailed confidence interval for difference of means: -0,266 to 0,216

Two-tailed P-value = 0,698

The difference in the mean values of the two groups is not great enough to reject the possibility that the difference is due to random sampling variability. There is not a statistically significant difference between the input groups (P = 0,698).

One-tailed P-value = 0,349

The sample mean of group Exp 3_control does not exceed the sample mean of the group Exp 3_sem-1a by an amount great enough to exclude the possibility that the difference is due to random sampling variability. The hypothesis that the population mean of group Exp 3_sem-1a is greater than or equal to the population mean of group Exp 3_control cannot be rejected. (P = 0,349).

Equal Variances Not Assumed (Welch's t-test):

t = -0,447 with 1,471 degrees of freedom.

95 percent two-tailed confidence interval for difference of means: -0,735 to 0,685

Two-tailed P-value = 0,712

The difference in the mean values of the two groups is not great enough to reject the possibility that the difference is due to random sampling variability. There is not a statistically significant difference between the input groups (P = 0,712).

One-tailed P-value = 0,356

The sample mean of group Exp 3_control does not exceed the sample mean of the group Exp 3_sem-1a by an amount great enough to exclude the possibility that the difference is due to random sampling variability. The hypothesis that the population mean of group Exp 3_sem-1a is greater than or equal to the population mean of group Exp 3_control cannot be rejected. (P = 0,356).

t-test_ Survival numbers yeast feeding assays

Normality Test (Shapiro-Wilk): Passed (P = 0,683)

Equal Variance Test (Brown-Forsythe): Failed (P < 0,050)

The result of the equal variance test indicates the likelihood that the two groups are sampled from populations with equal variances, but does not guarantee the equality or inequality of the two variances.

Group Name	N	Missing	Mean	Std Dev	SEM
Exp 4_Sem-1a	2	0	0,800	0,000	0,000
Exp 4_control	2	0	0,875	0,0354	0,0250

Difference of means -0,0750

Use the results of Welch's test, where equal variances are not assumed, if the equality of the population variances of the two groups is in doubt.

Equal Variances Assumed (Student's t-test):

t = -3,000 with 2 degrees of freedom.

95 percent two-tailed confidence interval for difference of means: -0,183 to 0,0326

Two-tailed P-value = 0,0955

The difference in the mean values of the two groups is not great enough to reject the possibility that the difference is due to random sampling variability. There is not a statistically significant difference between the input groups (P = 0,095).

One-tailed P-value = 0,0477

The sample mean of group Exp 4_control exceeds the sample mean of group Exp 4_Sem-1a by an amount that is greater than would be expected by chance, rejecting the hypothesis that the population mean of group Exp 4_Sem-1a is greater than or equal to the population mean of group Exp 4_control. (P = 0,048).

Equal Variances Not Assumed (Welch's t-test):

t = -3,000 with 1,000 degrees of freedom.

95 percent two-tailed confidence interval for difference of means: -0,393 to 0,243

Two-tailed P-value = 0,205

The difference in the mean values of the two groups is not great enough to reject the possibility that the difference is due to random sampling variability. There is not a statistically significant difference between the input groups (P = 0,205).

One-tailed P-value = 0,102

The sample mean of group Exp 4_control does not exceed the sample mean of the group Exp 4_Sem-1a by an amount great enough to exclude the possibility that the difference is due to random sampling variability. The hypothesis that the population mean of group Exp 4_Sem-1a is greater than or equal to the population mean of group Exp 4_control cannot be rejected. (P = 0,102).

t-test_ Survival numbers yeast feeding assays

Normality Test (Shapiro-Wilk): Failed (P < 0,050)

Equal Variance Test (Brown-Forsythe): Failed (P < 0,050)

The result of the equal variance test indicates the likelihood that the two groups are sampled from populations with equal variances, but does not guarantee the equality or inequality of the two variances.

Group Name	N	Missing	Mean	Std Dev	SEM
Exp 5_Sem-1a	2	0	0,867	0,0471	0,0333
Exp 5_control	2	0	0,800	0,0471	0,0333

Difference of means 0,0667

Use the results of Welch's test, where equal variances are not assumed, if the equality of the population variances of the two groups is in doubt.

Equal Variances Assumed (Student's t-test):

t = 1,414 with 2 degrees of freedom.

95 percent two-tailed confidence interval for difference of means: -0,136 to 0,269

Two-tailed P-value = 0,293

The difference in the mean values of the two groups is not great enough to reject the possibility that the difference is due to random sampling variability. There is not a statistically significant difference between the input groups (P = 0,293).

One-tailed P-value = 0,146

The sample mean of group Exp 5_Sem-1a does not exceed the sample mean of the group Exp 5_control by an amount great enough to exclude the possibility that the difference is due to random sampling variability. The hypothesis that the population mean of group Exp 5_control is greater than or equal to the population mean of group Exp 5_Sem-1a cannot be rejected. (P = 0,146).

Equal Variances Not Assumed (Welch's t-test):

t = 1,414 with 2,000 degrees of freedom.

95 percent two-tailed confidence interval for difference of means: -0,136 to 0,269

Two-tailed P-value = 0,293

The difference in the mean values of the two groups is not great enough to reject the possibility that the difference is due to random sampling variability. There is not a statistically significant difference between the input groups (P = 0,293).

One-tailed P-value = 0,146

The sample mean of group Exp 5_Sem-1a does not exceed the sample mean of the group Exp 5_control by an amount great enough to exclude the possibility that the difference is due to random sampling variability. The hypothesis that the population mean of group Exp 5_control is greater than or equal to the population mean of group Exp 5_Sem-1a cannot be rejected. (P = 0,146).

t-test_ Survival numbers yeast feeding assays

Normality Test (Shapiro-Wilk): Passed (P = 0,101)

Equal Variance Test (Brown-Forsythe): Failed (P < 0,050)

The result of the equal variance test indicates the likelihood that the two groups are sampled from populations with equal variances, but does not guarantee the equality or inequality of the two variances.

Group Name	N	Missing	Mean	Std Dev	SEM
asinsqrt(col(20))	2	0	1,200	0,0699	0,0494
asinsqrt(col(21))	2	0	1,108	0,0591	0,0418

Difference of means 0,0912

Use the results of Welch's test, where equal variances are not assumed, if the equality of the population variances of the two groups is in doubt.

Equal Variances Assumed (Student's t-test):

t = 1,409 with 2 degrees of freedom.

95 percent two-tailed confidence interval for difference of means: -0,187 to 0,370

Two-tailed P-value = 0,294

The difference in the mean values of the two groups is not great enough to reject the possibility that the difference is due to random sampling variability. There is not a statistically significant difference between the input groups (P = 0,294).

One-tailed P-value = 0,147

The sample mean of group asinsqrt(col(20)) does not exceed the sample mean of the group asinsqrt(col(21)) by an amount great enough to exclude the possibility that the difference is due to random sampling variability. The hypothesis that the population mean of group asinsqrt(col(21)) is greater than or equal to the population mean of group asinsqrt(col(20)) cannot be rejected. (P = 0,147).

Equal Variances Not Assumed (Welch's t-test):

t = 1,409 with 1,947 degrees of freedom.

95 percent two-tailed confidence interval for difference of means: -0,731 to 0,913

Two-tailed P-value = 0,297

The difference in the mean values of the two groups is not great enough to reject the possibility that the difference is due to random sampling variability. There is not a statistically significant difference between the input groups (P = 0,297).

One-tailed P-value = 0,149

The sample mean of group asinsqrt(col(20)) does not exceed the sample mean of the group asinsqrt(col(21)) by an amount great enough to exclude the possibility that the difference is due to random sampling variability. The hypothesis that the population mean of group asinsqrt(col(21)) is greater than or equal to the population mean of group asinsqrt(col(20)) cannot be rejected. (P = 0,149).

t-test_ Survival numbers yeast feeding assays

Normality Test (Shapiro-Wilk): Passed (P = 0,059)

Equal Variance Test (Brown-Forsythe): Passed (P = 1,000)

The result of the equal variance test indicates the likelihood that the two groups are sampled from populations with equal variances, but does not guarantee the equality or inequality of the two variances.

Group Name	N	Missing	Mean	Std Dev	SEM
Exp 6_Sem-1a	3	0	0,856	0,0509	0,0294
Exp 6_control	3	0	0,556	0,0770	0,0444

Difference of means 0,300

Use the results of Welch's test, where equal variances are not assumed, if the equality of the population variances of the two groups is in doubt.

Equal Variances Assumed (Student's t-test):

t = 5,630 with 4 degrees of freedom.

95 percent two-tailed confidence interval for difference of means: 0,152 to 0,448

Two-tailed P-value = 0,00490

The difference in the mean values of the two groups is greater than would be expected by chance; there is a statistically significant difference between the input groups (P = 0,005).

One-tailed P-value = 0,00245

The sample mean of group Exp 6_Sem-1a exceeds the sample mean of group Exp 6_control by an amount that is greater than would be expected by chance, rejecting the hypothesis that the population mean of group Exp 6_control is greater than or equal to the population mean of group Exp 6_Sem-1a. (P = 0,002).

Equal Variances Not Assumed (Welch's t-test):

t = 5,630 with 3,469 degrees of freedom.

95 percent two-tailed confidence interval for difference of means: 0,143 to 0,457

Two-tailed P-value = 0,00742

The difference in the mean values of the two groups is greater than would be expected by chance; there is a statistically significant difference between the input groups (P = 0,007).

One-tailed P-value = 0,00371

The sample mean of group Exp 6_Sem-1a exceeds the sample mean of group Exp 6_control by an amount that is greater than would be expected by chance, rejecting the hypothesis that the population mean of group Exp 6_control is greater than or equal to the population mean of group Exp 6_Sem-1a. (P = 0,004).

t-test_ Survival numbers yeast feeding assays

Normality Test (Shapiro-Wilk): Passed (P = 0,190)

Equal Variance Test (Brown-Forsythe): Passed (P = 1,000)

The result of the equal variance test indicates the likelihood that the two groups are sampled from populations with equal variances, but does not guarantee the equality or inequality of the two variances.

Group Name	N	Missing	Mean	Std Dev	SEM
Exp 7_Sem-1a	3	0	0,778	0,0192	0,0111
Exp 7_control	3	0	0,767	0,115	0,0667

Difference of means 0,0111

Use the results of Welch's test, where equal variances are not assumed, if the equality of the population variances of the two groups is in doubt.

Equal Variances Assumed (Student's t-test):

t = 0,164 with 4 degrees of freedom.

95 percent two-tailed confidence interval for difference of means: -0,177 to 0,199

Two-tailed P-value = 0,877

The difference in the mean values of the two groups is not great enough to reject the possibility that the difference is due to random sampling variability. There is not a statistically significant difference between the input groups (P = 0,877).

One-tailed P-value = 0,439

The sample mean of group Exp 7_Sem-1a does not exceed the sample mean of the group Exp 7_control by an amount great enough to exclude the possibility that the difference is due to random sampling variability. The hypothesis that the population mean of group Exp 7_control is greater than or equal to the population mean of group Exp 7_Sem-1a cannot be rejected. (P = 0,439).

Equal Variances Not Assumed (Welch's t-test):

t = 0,164 with 2,111 degrees of freedom.

95 percent two-tailed confidence interval for difference of means: -0,280 to 0,302

Two-tailed P-value = 0,884

The difference in the mean values of the two groups is not great enough to reject the possibility that the difference is due to random sampling variability. There is not a statistically significant difference between the input groups (P = 0,884).

One-tailed P-value = 0,442

The sample mean of group Exp 7_Sem-1a does not exceed the sample mean of the group Exp 7_control by an amount great enough to exclude the possibility that the difference is due to random sampling variability. The hypothesis that the population mean of group Exp 7_control is greater than or equal to the population mean of group Exp 7_Sem-1a cannot be rejected. (P = 0,442).

t-test_ Survival numbers yeast feeding assays

Normality Test (Shapiro-Wilk): Passed (P = 0,683)

Equal Variance Test (Brown-Forsythe): Failed (P < 0,050)

The result of the equal variance test indicates the likelihood that the two groups are sampled from populations with equal variances, but does not guarantee the equality or inequality of the two variances.

Group Name	N	Missing	Mean	Std Dev	SEM
Exp 3_lrc	2	0	0,900	0,000	0,000
Exp 3_control	2	0	0,950	0,0707	0,0500

Difference of means -0,0500

Use the results of Welch's test, where equal variances are not assumed, if the equality of the population variances of the two groups is in doubt.

Equal Variances Assumed (Student's t-test):

t = -1,000 with 2 degrees of freedom.

95 percent two-tailed confidence interval for difference of means: -0,265 to 0,165

Two-tailed P-value = 0,423

The difference in the mean values of the two groups is not great enough to reject the possibility that the difference is due to random sampling variability. There is not a statistically significant difference between the input groups (P = 0,423).

One-tailed P-value = 0,211

The sample mean of group Exp 3_control does not exceed the sample mean of the group Exp 3_lrc by an amount great enough to exclude the possibility that the difference is due to random sampling variability. The hypothesis that the population mean of group Exp 3_lrc is greater than or equal to the population mean of group Exp 3_control cannot be rejected. (P = 0,211).

Equal Variances Not Assumed (Welch's t-test):

t = -1,000 with 1,000 degrees of freedom.

95 percent two-tailed confidence interval for difference of means: -0,685 to 0,585

Two-tailed P-value = 0,500

The difference in the mean values of the two groups is not great enough to reject the possibility that the difference is due to random sampling variability. There is not a statistically significant difference between the input groups (P = 0,500).

One-tailed P-value = 0,250

The sample mean of group Exp 3_control does not exceed the sample mean of the group Exp 3_lrc by an amount great enough to exclude the possibility that the difference is due to random sampling variability. The hypothesis that the population mean of group Exp 3_lrc is greater than or equal to the population mean of group Exp 3_control cannot be rejected. (P = 0,250).

t-test_ Survival numbers yeast feeding assays

Normality Test (Shapiro-Wilk): Passed (P = 0,962)

Equal Variance Test (Brown-Forsythe): Failed (P < 0,050)

The result of the equal variance test indicates the likelihood that the two groups are sampled from populations with equal variances, but does not guarantee the equality or inequality of the two variances.

Group Name	N	Missing	Mean	Std Dev	SEM
Exp 4_lrc	2	0	0,675	0,247	0,175
Exp 4_control	2	0	0,875	0,0354	0,0250

Difference of means -0,200

Use the results of Welch's test, where equal variances are not assumed, if the equality of the population variances of the two groups is in doubt.

Equal Variances Assumed (Student's t-test):

t = -1,131 with 2 degrees of freedom.

95 percent two-tailed confidence interval for difference of means: -0,961 to 0,561

Two-tailed P-value = 0,375

The difference in the mean values of the two groups is not great enough to reject the possibility that the difference is due to random sampling variability. There is not a statistically significant difference between the input groups (P = 0,375).

One-tailed P-value = 0,188

The sample mean of group Exp 4_control does not exceed the sample mean of the group Exp 4_lrc by an amount great enough to exclude the possibility that the difference is due to random sampling variability. The hypothesis that the population mean of group Exp 4_lrc is greater than or equal to the population mean of group Exp 4_control cannot be rejected. (P = 0,188).

Equal Variances Not Assumed (Welch's t-test):

t = -1,131 with 1,041 degrees of freedom.

95 percent two-tailed confidence interval for difference of means: -2,446 to 2,046

Two-tailed P-value = 0,455

The difference in the mean values of the two groups is not great enough to reject the possibility that the difference is due to random sampling variability. There is not a statistically significant difference between the input groups (P = 0,455).

One-tailed P-value = 0,228

The sample mean of group Exp 4_control does not exceed the sample mean of the group Exp 4_lrc by an amount great enough to exclude the possibility that the difference is due to random sampling variability. The hypothesis that the population mean of group Exp 4_lrc is greater than or equal to the population mean of group Exp 4_control cannot be rejected. (P = 0,228).

t-test_ Survival numbers yeast feeding assays

Normality Test (Shapiro-Wilk): Passed (P = 0,588)

Equal Variance Test (Brown-Forsythe): Passed (P = 1,000)

The result of the equal variance test indicates the likelihood that the two groups are sampled from populations with equal variances, but does not guarantee the equality or inequality of the two variances.

Group Name	N	Missing	Mean	Std Dev	SEM
Exp 6_lrc	3	0	0,467	0,186	0,107
Exp 6_control	3	0	0,556	0,0770	0,0444

Difference of means -0,0889

Use the results of Welch's test, where equal variances are not assumed, if the equality of the population variances of the two groups is in doubt.

Equal Variances Assumed (Student's t-test):

t = -0,766 with 4 degrees of freedom.

95 percent two-tailed confidence interval for difference of means: -0,411 to 0,233

Two-tailed P-value = 0,486

The difference in the mean values of the two groups is not great enough to reject the possibility that the difference is due to random sampling variability. There is not a statistically significant difference between the input groups (P = 0,486).

One-tailed P-value = 0,243

The sample mean of group Exp 6_control does not exceed the sample mean of the group Exp 6_lrc by an amount great enough to exclude the possibility that the difference is due to random sampling variability. The hypothesis that the population mean of group Exp 6_lrc is greater than or equal to the population mean of group Exp 6_control cannot be rejected. (P = 0,243).

Equal Variances Not Assumed (Welch's t-test):

t = -0,766 with 2,668 degrees of freedom.

95 percent two-tailed confidence interval for difference of means: -0,588 to 0,410

Two-tailed P-value = 0,506

The difference in the mean values of the two groups is not great enough to reject the possibility that the difference is due to random sampling variability. There is not a statistically significant difference between the input groups (P = 0,506).

One-tailed P-value = 0,253

The sample mean of group Exp 6_control does not exceed the sample mean of the group Exp 6_lrc by an amount great enough to exclude the possibility that the difference is due to random sampling variability. The hypothesis that the population mean of group Exp 6_lrc is greater than or equal to the population mean of group Exp 6_control cannot be rejected. (P = 0,253).

t-test_ Survival numbers yeast feeding assays

Normality Test (Shapiro-Wilk): Passed (P = 0,110)

Equal Variance Test (Brown-Forsythe): Passed (P = 1,000)

The result of the equal variance test indicates the likelihood that the two groups are sampled from populations with equal variances, but does not guarantee the equality or inequality of the two variances.

**Group Name**	**N**	**Missing**	**Mean**	**Std Dev**	**SEM**
Exp 7_lrc	3	0	0,767	0,0667	0,0385
Exp 7_control	3	0	0,767	0,115	0,0667

Difference of means 0,000

Use the results of Welch's test, where equal variances are not assumed, if the equality of the population variances of the two groups is in doubt.

Equal Variances Assumed (Student's t-test):

t = 0,000 with 4 degrees of freedom.

95 percent two-tailed confidence interval for difference of means: -0,214 to 0,214

Two-tailed P-value = 1,000

The difference in the mean values of the two groups is not great enough to reject the possibility that the difference is due to random sampling variability. There is not a statistically significant difference between the input groups (P = 1,000).

One-tailed P-value = 1,000

The sample mean of group Exp 7_control does not exceed the sample mean of the group Exp 7_lrc by an amount great enough to exclude the possibility that the difference is due to random sampling variability. The hypothesis that the population mean of group Exp 7_lrc is greater than or equal to the population mean of group Exp 7_control cannot be rejected. (P = 1,000).

Equal Variances Not Assumed (Welch's t-test):

t = 0,000 with 3,200 degrees of freedom.

95 percent two-tailed confidence interval for difference of means: -0,237 to 0,237

Two-tailed P-value = 1,000

The difference in the mean values of the two groups is not great enough to reject the possibility that the difference is due to random sampling variability. There is not a statistically significant difference between the input groups (P = 1,000).

One-tailed P-value = 1,000

The sample mean of group Exp 7_control does not exceed the sample mean of the group Exp 7_lrc by an amount great enough to exclude the possibility that the difference is due to random sampling variability. The hypothesis that the population mean of group Exp 7_lrc is greater than or equal to the population mean of group Exp 7_control cannot be rejected. (P = 1,000).

t-test_ Survival numbers yeast feeding assays

Normality Test (Shapiro-Wilk): Passed (P = 0,348)

Equal Variance Test (Brown-Forsythe): Failed (P < 0,050)

The result of the equal variance test indicates the likelihood that the two groups are sampled from populations with equal variances, but does not guarantee the equality or inequality of the two variances.

Group Name	N	Missing	Mean	Std Dev	SEM
Exp 3_fez2	2	0	0,925	0,106	0,0750
Exp 3_control	2	0	0,950	0,0707	0,0500

Difference of means -0,0250

Use the results of Welch's test, where equal variances are not assumed, if the equality of the population variances of the two groups is in doubt.

Equal Variances Assumed (Student's t-test):

t = -0,277 with 2 degrees of freedom.

95 percent two-tailed confidence interval for difference of means: -0,413 to 0,363

Two-tailed P-value = 0,808

The difference in the mean values of the two groups is not great enough to reject the possibility that the difference is due to random sampling variability. There is not a statistically significant difference between the input groups (P = 0,808).

One-tailed P-value = 0,404

The sample mean of group Exp 3_control does not exceed the sample mean of the group Exp 3_fez2 by an amount great enough to exclude the possibility that the difference is due to random sampling variability. The hypothesis that the population mean of group Exp 3_fez2 is greater than or equal to the population mean of group Exp 3_control cannot be rejected. (P = 0,404).

Equal Variances Not Assumed (Welch's t-test):

t = -0,277 with 1,742 degrees of freedom.

95 percent two-tailed confidence interval for difference of means: -1,170 to 1,120

Two-tailed P-value = 0,811

The difference in the mean values of the two groups is not great enough to reject the possibility that the difference is due to random sampling variability. There is not a statistically significant difference between the input groups (P = 0,811).

One-tailed P-value = 0,405

The sample mean of group Exp 3_control does not exceed the sample mean of the group Exp 3_fez2 by an amount great enough to exclude the possibility that the difference is due to random sampling variability. The hypothesis that the population mean of group Exp 3_fez2 is greater than or equal to the population mean of group Exp 3_control cannot be rejected. (P = 0,405).

t-test_ Survival numbers yeast feeding assays

Normality Test (Shapiro-Wilk): Passed (P = 0,714)

Equal Variance Test (Brown-Forsythe): Failed (P < 0,050)

The result of the equal variance test indicates the likelihood that the two groups are sampled from populations with equal variances, but does not guarantee the equality or inequality of the two variances.

Group Name	N	Missing	Mean	Std Dev	SEM
Exp 4_fez2	2	0	0,500	0,0707	0,0500
Exp 4_control	2	0	0,875	0,0354	0,0250

Difference of means -0,375

Use the results of Welch's test, where equal variances are not assumed, if the equality of the population variances of the two groups is in doubt.

Equal Variances Assumed (Student's t-test):

t = -6,708 with 2 degrees of freedom.

95 percent two-tailed confidence interval for difference of means: -0,616 to -0,134

Two-tailed P-value = 0,0215

The difference in the mean values of the two groups is greater than would be expected by chance; there is a statistically significant difference between the input groups (P = 0,022).

One-tailed P-value = 0,0108

The sample mean of group Exp 4_control exceeds the sample mean of group Exp 4_fez2 by an amount that is greater than would be expected by chance, rejecting the hypothesis that the population mean of group Exp 4_fez2 is greater than or equal to the population mean of group Exp 4_control. (P = 0,011).

Equal Variances Not Assumed (Welch's t-test):

t = -6,708 with 1,471 degrees of freedom.

95 percent two-tailed confidence interval for difference of means: -1,085 to 0,335

Two-tailed P-value = 0,0445

The difference in the mean values of the two groups is greater than would be expected by chance; there is a statistically significant difference between the input groups (P = 0,045).

One-tailed P-value = 0,0223

The sample mean of group Exp 4_control exceeds the sample mean of group Exp 4_fez2 by an amount that is greater than would be expected by chance, rejecting the hypothesis that the population mean of group Exp 4_fez2 is greater than or equal to the population mean of group Exp 4_control. (P = 0,022).

t-test_ Survival numbers yeast feeding assays

Normality Test (Shapiro-Wilk): Passed (P = 0,078)

Equal Variance Test (Brown-Forsythe): Passed (P = 1,000)

The result of the equal variance test indicates the likelihood that the two groups are sampled from populations with equal variances, but does not guarantee the equality or inequality of the two variances.

**Group Name**	**N**	**Missing**	**Mean**	**Std Dev**	**SEM**
Exp 6_fez2	3	0	0,767	0,000	0,000
Exp 6_control	3	0	0,556	0,0770	0,0444

Difference of means 0,211

Use the results of Welch's test, where equal variances are not assumed, if the equality of the population variances of the two groups is in doubt.

Equal Variances Assumed (Student's t-test):

t = 4,750 with 4 degrees of freedom.

95 percent two-tailed confidence interval for difference of means: 0,0877 to 0,335

Two-tailed P-value = 0,00897

The difference in the mean values of the two groups is greater than would be expected by chance; there is a statistically significant difference between the input groups (P = 0,009).

One-tailed P-value = 0,00449

The sample mean of group Exp 6_fez2 exceeds the sample mean of group Exp 6_control by an amount that is greater than would be expected by chance, rejecting the hypothesis that the population mean of group Exp 6_control is greater than or equal to the population mean of group Exp 6_fez2. (P = 0,004).

Equal Variances Not Assumed (Welch's t-test):

t = 4,750 with 2,000 degrees of freedom.

95 percent two-tailed confidence interval for difference of means: 0,0199 to 0,402

Two-tailed P-value = 0,0416

The difference in the mean values of the two groups is greater than would be expected by chance; there is a statistically significant difference between the input groups (P = 0,042).

One-tailed P-value = 0,0208

The sample mean of group Exp 6_fez2 exceeds the sample mean of group Exp 6_control by an amount that is greater than would be expected by chance, rejecting the hypothesis that the population mean of group Exp 6_control is greater than or equal to the population mean of group Exp 6_fez2. (P = 0,021).

t-test_ Survival numbers yeast feeding assays

Normality Test (Shapiro-Wilk): Passed (P = 0,091)

Equal Variance Test (Brown-Forsythe): Passed (P = 1,000)

The result of the equal variance test indicates the likelihood that the two groups are sampled from populations with equal variances, but does not guarantee the equality or inequality of the two variances.

Group Name	N	Missing	Mean	Std Dev	SEM
Exp 7_fez2	3	0	0,833	0,0577	0,0333
Exp 7_control	3	0	0,767	0,115	0,0667

Difference of means 0,0667

Use the results of Welch's test, where equal variances are not assumed, if the equality of the population variances of the two groups is in doubt.

Equal Variances Assumed (Student's t-test):

t = 0,894 with 4 degrees of freedom.

95 percent two-tailed confidence interval for difference of means: -0,140 to 0,274

Two-tailed P-value = 0,422

The difference in the mean values of the two groups is not great enough to reject the possibility that the difference is due to random sampling variability. There is not a statistically significant difference between the input groups (P = 0,422).

One-tailed P-value = 0,211

The sample mean of group Exp 7_fez2 does not exceed the sample mean of the group Exp 7_control by an amount great enough to exclude the possibility that the difference is due to random sampling variability. The hypothesis that the population mean of group Exp 7_control is greater than or equal to the population mean of group Exp 7_fez2 cannot be rejected. (P = 0,211).

Equal Variances Not Assumed (Welch's t-test):

t = 0,894 with 2,941 degrees of freedom.

95 percent two-tailed confidence interval for difference of means: -0,254 to 0,387

Two-tailed P-value = 0,438

The difference in the mean values of the two groups is not great enough to reject the possibility that the difference is due to random sampling variability. There is not a statistically significant difference between the input groups (P = 0,438).

One-tailed P-value = 0,219

The sample mean of group Exp 7_fez2 does not exceed the sample mean of the group Exp 7_control by an amount great enough to exclude the possibility that the difference is due to random sampling variability. The hypothesis that the population mean of group Exp 7_control is greater than or equal to the population mean of group Exp 7_fez2 cannot be rejected. (P = 0,219).
