# Supplementary material for: Challenges of Robust RNAi-Mediated Gene Silencing in Aedes Mosquitoes
Source: Int J Mol Sci. 2024 May 10;25(10):5218. doi: 10.3390/ijms25105218 (PMC11121262; doi:10.3390/ijms25105218)
Supplement: Supplementary file 1 [file ijms-25-05218-s001.zip › Table S5.pdf]

Table S5. Survival rates of *Ae. albopictus* and *Ae. aegypti* following feeding with bacterially produced shRNAs targeting *fez2, lrc* and nuclease-encoding genes. Each replicate contained n = 40 neonate larvae at the start. In some of the replicates (treatment and control), the agar pellets used for dsRNA feeding were hardly consumed over time and larvae died from starvation. In some replicates, the target-specific shRNA was combined with an shRNA against a gut-specific nuclease. Two types of negative controls were used: « none » means that no bacterially expressed shRNA was added to the agar pellet, « scramble » means that unspecific bacterial-expressed shRNA was added to the agar pellet.

Species	Strain	Treatment	Replicate no.	Total L1	Pupae + adults	Larvae	Dead	Total alive (% survival)
Ae. albopictus	Montpellier	**nuclease 1**	1	40	22	18	0	40 (100%)
		**nuclease 1**	2	40	18	22	0	40 (100%)
		fez2	1	40	6	34	0	40 (100%)
		***fez2* + nuclease 1**	1	40	18	21	1	39 (97.5%)
		**none**	1	40	0	40	0	40 (100%)
		**scramble**	1	40	20	20	0	40 (100%)
		**scramble**	2	40	18	22	0	40 (100%)
Ae. aegypti	La Réunion	***fez2* v1**	1	40	23	2	15	25 (62.5%)
		***fez2* v1 + nuclease**	1	40	33	2	5	35 (87.5%)
		**none**	1	40	30	5	5	35 (87.5%)
		**scramble**	1	40	23	1	16	24 (55%)
		**scramble**	2	40	24	3	13	27 (67.5%)
	La Réunion	***lrc***	1	40	14	19	7	33 (82.5%)
		lrc	2	40	19	8	13	27 (67.5%)
		***fez2* v2**	1	40	12	21	7	33 (82.5%)
		***fez2* v2**	2	40	14	18	8	32 (80%)
		**none**	1	40	19	20	1	39 (97.5%)
		**scramble**	1	40	20	19	1	39 (97.5%)
	Liverpool	***lrc***	1	40	6	32	2	38 (95%)
		lrc	2	40	0	27	13	27 (67.5%)
		***fez2* v2**	1	45	1	44	0	45 (100%)
		***fez2* v2**	2	40	0	12	28	12 (30%)
		**none**	1	44	3	41	0	44 (100%)
		**scramble**	1	40	1	30	9	31 (77.5%)
