# Supplementary material for: Challenges of Robust RNAi-Mediated Gene Silencing in Aedes Mosquitoes
Source: Int J Mol Sci. 2024 May 10;25(10):5218. doi: 10.3390/ijms25105218 (PMC11121262; doi:10.3390/ijms25105218)
Supplement: Supplementary file 1 [file ijms-25-05218-s001.zip › Table S6.pdf]

Figueiredo Prates et al. (2024) IJMS

Table S6. Sex-ratio and survival rates in *Ae. albopictus* (Montpellier strain) fed with bacterially produced shRNAs targeted against two female-specific isoforms of *doublesex (dsxF1 and dsxF2)*. Two rounds of experiments were carried out. Replicates annotated with an asterisk were performed on a different day than replicates without. Each replicate contained n = 50 neonate *Ae. albopictus* larvae at the start. In some of the replicates (treatment and control), the agar pellets used for dsRNA feeding were hardly consumed over time. Larval development being much slower in such replicates, counting was stopped at day 30 even though there remained young larvae whose sex could not be determined. *dsx*-shRNAs were combined with shRNAs against three different gut nucleases of *Ae. albopictus*. Two types of negative controls were used: « none » means that no bacterially expressed shRNA was added to the agar pellet, « scramble » means that an unspecific bacterially expressed shRNA was added to the agar pellet.

Treatment	Replicate	No. (%) males	No. (%) females	Could not be sexed	Reached adulthood (%)	Remaining larvae/ pupae after 1 month
***dsxF1* + *dsxF2* + 3 nucleases**	1	17 (50.0 %)	17 (50.0 %)	0	34 (68.0 %)	5
***dsxF1* + *dsxF2* + 3 nucleases**	2	19 (54.3 %)	16 (45.7 %)	0	35 (70.0 %)	1
***dsxF1* + *dsxF2* + 3 nucleases**	3*	12 (38.7 %)	19 (61.3 %)	0	31 (62 %)	0
***dsxF1* + *dsxF2* + 3 nucleases**	4*	11 (45.7%)	19 (54.3%)	5	35 (70%)	0
***dsxF1* + *dsxF2* + 3 nucleases**	5*	13 (40.0%)	21 (60.0%)	1	35 (70%)	0
**none**	1	12 (60.0 %)	8 (40.0%)	0	20 (40.0 %)	15
**none**	2*	14 (50.0%)	14 (50.0%)	2	30 (60%)	0
**scramble**	1	18 (56.3 %)	14 (43.8%)	0	32 (64.0 %)	2
**scramble**	2	14 (48.3 %)	15 (51.7%)	0	29 (58.0 %)	2
**scramble**	3*	13 (35.1%)	24 (64.9 %)	0	37 (74.0 %)	0
